# Overcoming Biopotency Barriers: Advanced Oral Delivery Strategies for Enhancing the Efficacy of Bioactive Food Ingredients

**DOI:** 10.1002/advs.202401172

**Published:** 2024-10-03

**Authors:** Ling Liu, David Julian McClements, Xuebo Liu, Fuguo Liu

**Affiliations:** ^1^ College of Food Science and Engineering Northwest A&F University Yangling Shaanxi 712100 China; ^2^ Department of Food Science University of Massachusetts Amherst Amherst MA 01003 USA

**Keywords:** biopotency barriers, functional foods, oral delivery systems, physiological benefits, targeted release

## Abstract

Bioactive food ingredients contribute to the promotion and maintenance of human health and wellbeing. However, these functional ingredients often exhibit low biopotency after food processing or gastrointestinal transit. Well‐designed oral delivery systems can increase the ability of bioactive food ingredients to resist harsh environments inside and outside the human body, as well as allow for controlled or triggered release of bioactives to specific sites in the gastrointestinal tract or other tissues and organs. This review presents the characteristics of common bioactive food ingredients and then highlights the barriers to their biopotency. It also discusses various oral delivery strategies and carrier types that can be used to overcome these biopotency barriers, with a focus on recent advances in the field. Additionally, the advantages and disadvantages of different delivery strategies are highlighted. Finally, the current challenges facing the development of food‐grade oral delivery systems are addressed, and areas where future research can lead to new advances and industrial applications of these systems are proposed.

## Graphical abstract

This review mainly includes the following parts: 1) Categories and properties of staple bioactive food ingredients; 2) Biopotency barriers faced by common food bioactive substances; 3) Current progress, advantages and disadvantages of diverse oral delivery strategies for overcoming multiple in vitro and in vivo biopotency barriers; 4) Present challenges and future perspectives for food‐grade materials and oral delivery systems.

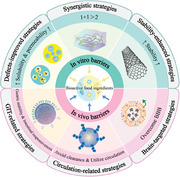

## 1 Introduction

Foodstuffs contain a variety of nutrients and other bioactive ingredients, including polysaccharides, proteins, lipids, polyphenols, terpenes, vitamins, minerals, alkaloids, prebiotics, and probiotics. These ingredients may have beneficial biological activities that can prevent or alleviate certain chronic diseases.^[^ ^1^, ^2^ ^]^ In response to consumer demands for healthier diets, there has been a growing interest in the development of personalized nutrition interventions through functional foods.^[^ ^3^ ^]^ These functional foods are often enriched with bioactive agents that exhibit beneficial biological activities, such as anticancer, antioxidant, anti‐inflammatory, or anti‐diabetic activities.^[^ ^4^ ^]^ However, the molecular, physicochemical, and physiological attributes of these bioactive agents depend on their composition and structural organization. It is critical to understand the nature and behavior of bioactive ingredients in functional foods to enhance their performance. For example, many bioactive agents, including unsaturated fatty acids, polyphenols, and carotenoids, are susceptible to chemical degradation when exposed to environmental stressors, such as light, oxygen, and heat, during processing, distribution, and storage. This chemical instability is due to the presence of unsaturated bonds or phenolic hydroxyl groups.^[^ ^5^ ^]^ Furthermore, these bioactive agents also experience harsh conditions during their passage through the human gastrointestinal tract (GIT), including chewing, swallowing, corrosive gastric acids, bile salts, enzyme digestion, and metabolism. Consequently, the biopotency of these bioactive agents is reduced. Therefore, improving the stability of these ingredients in foods and the GIT is an important challenge.

**Figure 1:**
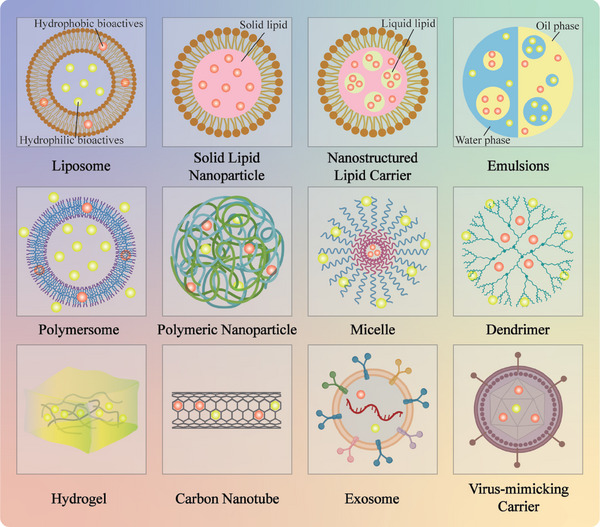
Common systems developed for oral delivery of bioactives.

Bioactive food ingredients can be protected from environmental stressors using appropriate encapsulation technologies, such as nanoparticles or microparticles, thereby enhancing their biopotency. Oral delivery carriers constructed from food‐grade components (such as proteins, polysaccharides, and lipids) are capable of protecting bioactive food ingredients from harsh in vitro processing conditions and in vivo gastrointestinal environments.^[^ ^6^ ^]^ As shown in **Figure** **1**, a variety of carriers have been successfully used to encapsulate, protect, and deliver bioactive food ingredients, including emulsions, solid lipid nanoparticles, liposomes, micelles, polymeric nanoparticles, dendrimers, hydrogels, nanotubes, and exosomes. Each type of carrier exhibits distinctive particle characteristics, including composition, size, shape, structure, charge, and environmental responsiveness. These characteristics determine the physicochemical and functional attributes of the delivery carriers. The properties of carriers are typically tailored based on the characteristics of the bioactive food ingredients, the nature of the food matrix it will be incorporated into, and the desired physiological effects. Delivery carriers should be designed to protect the encapsulated bioactives during food processing, distribution, and storage, as well as within the human GIT. Additionally, they should be designed to enhance the compatibility of the bioactive ingredients with the food matrix. For example, lipophilic bioactives, including essential oils, carotenoids, curcuminoids, and polyunsaturated fatty acids, have anti‐inflammatory, antimicrobial, and/or antioxidant effects. However, their water solubility and chemical stability are poor.^[^ ^7^ ^]^ Consequently, delivery systems should be designed to overcome these deficiencies.^[^ ^8^ ^]^ Although many researchers have developed oral delivery systems for precision nutrition and targeted therapy applications, there are still many challenges that need to be overcome. For instance, some of the delivery carriers constructed in some studies contain potentially toxic materials, and their long‐term safety profile has not been assessed.^[^ ^9^ ^]^ Moreover, the controlled release and targeting characteristics of many of the current generation of delivery systems are inadequate.^[^ ^10^ ^]^ These challenges therefore impede the advancement of precision nutrition and targeted delivery.

This review begins by introducing the different types of bioactive food ingredients that need to be delivered. It then discusses the various biopotency barriers currently limiting the efficacy of these ingredients during in vitro food processing, storage, and transport, as well as in vivo gastrointestinal transit and systemic circulation. Subsequently, the different oral delivery strategies and systems that have been developed to overcome these biopotency barriers are summarized. Finally, challenges in the development and application of oral delivery systems are highlighted, and future research directions are discussed. The objective of this review is to stimulate further research on the design and development of new and improved oral delivery systems, which may increase their practical applications in the food, nutritional, and medical fields.

## 2 Bioactivities of Food Ingredients

### 2.1 Bioactive Macromolecular Compounds

Foods contain a variety of bioactive macromolecules, including polysaccharides, proteins, and lipids, which have beneficial physiological activities and may serve as construction materials for bioactive delivery systems (Table S1, Supporting Information).^[^ ^11^ ^]^

- Bioactive polysaccharides are widely distributed in animals, plants, and microorganisms.^[^ ^12^ ^]^ In general, polysaccharides consist of ten or more monosaccharides (usually aldoses or ketoses) linked by glycosidic bonds, such as α‐1,4, β‐1,4, α‐1,6, β‐1,6 bonds.^[^ ^13^ ^]^ Polysaccharides may be composed of similar or different monosaccharides, and may be linear or branched. Furthermore, they may exhibit differences in molecular weight, polarity, and charge. These diverse molecular features provide the foundation for a multitude of biological functions in nature, including energy storage, structural support, cell adhesion, signaling, metabolism, immune defense, and molecular recognition. In foods, polysaccharides may exhibit a variety of biological activities depending on their molecular characteristics, including antioxidant, antimicrobial, anti‐inflammatory, antiviral, anticoagulant, anti‐fatigue, anti‐diabetic, anti‐cancer, anti‐tumor, immunoregulatory, and multi‐targeting capabilities.^[^ ^14^, ^15^, ^16^ ^]^ Many food polysaccharides are abundant, inexpensive, safe, and biocompatible. Consequently, many researchers have focused on the utilization of polysaccharides as functional ingredients for the construction of bioactive delivery systems in recent years.^[^ ^16^ ^]^

For example, chitosan, sodium alginate, and hyaluronic acid have been extensively used in the construction of food‐grade delivery systems for the oral administration of nutraceuticals or pharmaceuticals. Chitosan is a natural linear cationic polysaccharide derived from deacetylated chitin, which is commonly obtained from the exoskeletons of arthropods, crustaceans, and insects, as well as from fungal cell walls.^[^ ^17^ ^]^ Its structural composition consists of a series of glucosamine structural units (N‐acetyl‐D‐glucosamine and D‐glucosamine) linked by β‐1, 4‐glycosidic bonds. Alginate is a natural anionic polysaccharide extracted from brown seaweeds. It is composed of β‐D‐1, 4‐mannuronic, and α‐L‐1, 4‐guluronic acids.^[^ ^18^ ^]^ Hyaluronic acid is a natural linear acidic mucopolysaccharide found in animal tissues and microorganisms, which consists of alternating N‐acetylglucosamine and D‐glucuronic acid units.^[^ ^19^ ^]^ Polysaccharides and their functionalized derivatives have been used to treat skin wounds, achieve targeted delivery and controlled release, and prevent neurodegenerative, cardiovascular, and intestinal diseases through the formation of hydrogels, aerogels, microgels, microcapsules, microneedles, nanoparticles, vesicles, micelles, and other delivery systems.^[^ ^20^ ^]^

(b) Bioactive proteins are derived from diverse sources, including animals, plants, and microorganisms.^[^ ^21^ ^]^ The physicochemical, functional, and biological activities of proteins depend on the conformation of peptide chains and the number, type, and sequence of amino acids they contain. Proteins found in nature or produced by microbial fermentation can be hydrolyzed to produce peptides and amino acids, which have different biological activities than the parent molecules.^[^ ^22^ ^]^ Furthermore, the biological activities of polypeptides depend on their precise 3D conformation. Consequently, factors that may promote their denaturation, such as heat, cold, sonication, pH changes, inorganic salts, organic solvents, and detergents, should be controlled.^[^ ^23^ ^]^ In nature, proteins exhibit a range of biological activities, including energy sources, tissue construction, maintenance of metabolism, hormone regulation, signaling, transport, and immune system function.^[^ ^24^ ^]^ In foods and pharmaceuticals, bioactive proteins may also exhibit biological activities that are beneficial to human health, including antioxidant, antimicrobial, antihypertensive, anti‐lipidemic, anti‐tumor, anti‐inflammatory, and anti‐diabetic properties. Bioactive proteins may also be used as construction materials to fabricate delivery vehicles.^[^ ^24^, ^25^, ^26^ ^]^

Milk is one of the most abundant sources of bioactive proteins, comprising caseins (αs_1_, αs_2_, β, κ), whey proteins, lactalbumin, lactoferrin, and lactoglobulin.^[^ ^27^ ^]^ In general, casein represents ≈80% of the total protein content of milk, which is naturally present as acid‐sensitive casein micelles.^[^ ^28^ ^]^ Lactoferrin (LF) is a globular iron‐binding glycoprotein that exhibits a variety of biological activities, including antimicrobial, antioxidant, anti‐inflammatory, anti‐carcinogenic, neuroprotective, immune responsive, and tissue regenerative capacities.^[^ ^29^ ^]^ LF‐based materials have been used to encapsulate, protect, and deliver a variety of bioactive ingredients.^[^ ^30^ ^]^ Recently, there has been a growing interest in utilizing proteins derived from plant sources, such as wheat, corn, soy, peas, or potato, due to their environmental and sustainability benefits.^[^ ^24^ ^]^ For instance, wheat proteins include albumins, globulins, gliadins, and glutenins, which have different molecular structures, physicochemical properties, and functionalities. The presence of high levels of glutamine and non‐polar amino acids in gliadin enables it to form strong hydrogen bonds and hydrophobic interactions with the mucosa, significantly enhancing the biopotency of drugs and other bioactive substances.^[^ ^24^ ^]^

(c) Bioactive lipids encompass a diverse range of substances, including triacylglycerols, diacylglycerols, monoacylglycerols, free fatty acids, phospholipids, phytosterols, and cholesterol that are soluble in organic solvents but not in water.^[^ ^31^ ^]^ The polyunsaturated fatty acids (PUFAs) derived from fish, algal, and seed oils have been identified as having beneficial health effects, including a reduction in the risk of cardiovascular diseases and the protection of brain cells.^[^ ^32^ ^]^ One of the main obstacles to incorporating PUFAs into foods is that they contain multiple C═C bonds in their carbon skeleton, making them highly susceptible to oxidation. Moreover, they are highly hydrophobic molecules with low water solubility. Consequently, they often require encapsulation to enhance their dispersibility, stability, bioactivity, and biopotency.^[^ ^33^ ^]^ Phospholipids, including glycerophospholipids and sphingomyelin, are amphiphilic substances consisting of a glycerol backbone esterified with two fatty acids (lipophilic) and a modified phosphate group (hydrophilic), which are a major component of cell membranes.^[^ ^34^ ^]^ Sterols, including cholesterol, phytosterols (β‐sitosterol, stigmasterol), and ergosterol, are derivatives of cyclopentane polyhydrophenanthrene.^[^ ^35^ ^]^ Amphipathic phospholipids and hydrophobic sterols are typically delivered in foods and drugs in the form of liposomes, which are prepared using methods such as film dispersion, ethanol injection, and reverse evaporation.^[^ ^36^ ^]^ Liposomes are composed of concentric rings of phospholipid bilayers, with the non‐polar fatty acids facing inwards and the polar head groups facing outwards.

### 2.2 Bioactive Small Molecular Compounds

Foods also contain a variety of bioactive ingredients with relatively small molecular weights.^[^ ^37^ ^]^ For example, polyphenols, terpenes, vitamins, minerals, alkaloids, essential oils, and saponins exhibit a range of biological activities, including antioxidants, antimicrobials, antidiabetics, anticancer agents, colorants, and flavors.^[^ ^38^ ^]^ Many of these bioactive substances need to be encapsulated in delivery carriers to enhance their dispersibility, stability, or efficacy (Table S1, Supporting Information).

- Plant secondary metabolites (PSMs) are secondary metabolites produced by plants, including polyphenols, terpenoids, and nitrogenous substances. These compounds are not essential for the survival of the organism but possess beneficial physiological activities, such as preventing infections or deterring consumption by pests.^[^ ^39^ ^]^ Polyphenols are a class of substances with multiple phenolic hydroxyl groups on the benzene ring. This class includes flavonoids (e.g., quercetin, anthocyanins), phenolic acids (e.g., chlorogenic, caffeic, and cinnamic acids), lignans (e.g., sesamin, syringaresinol, and medioresinol), and stilbenes (e.g., resveratrol, piceatannol, and pinostilbene), each consisting of different basic structural units.^[^ ^40^, ^41^, ^42^, ^43^, ^44^ ^]^ Terpenoids and their derivatives are compounds that contain one or more isoprene units in their basic structure. They are usually classified according to the number of isoprene units (monoterpenoids, sesquiterpenoids, diterpenoids, triterpenoids, tetraterpenoids) and the type of functional groups they contain (alkene, alcohol, aldehyde, ketone, ester, carboxylic acid).^[^ ^45^ ^]^ Terpenoids exert their biological activities and physiological effects primarily in the form of pigments (carotenoids), flavors and fragrances (some types of essential oils), antioxidants, and antimicrobials (some types of alkaloids and saponins).^[^ ^46^, ^47^, ^48^, ^49^ ^]^ Nitrogenous secondary metabolites mainly refer to alkaloids, amino acids, and cyanogenic glycosides (toxic). The development of delivery systems is being pursued with the objective of enhancing the health‐promoting effects of PSMs by overcoming limiting factors, such as poor water solubility, stability, absorption, and biopotency.^[^ ^5^ ^]^
- Vitamins and minerals are the two principal categories of essential micronutrients, which play a pivotal role in maintaining normal physiological functions in humans.^[^ ^50^ ^]^ Vitamins exhibit a diverse range of molecular structures, resulting in their diverse range of physicochemical properties and biological activities. Vitamins can be classified as either lipid‐soluble (V_A_, V_D_, V_E_, V_K_) or water‐soluble (V_C_, V_B_) depending on their solubility characteristics. A significant proportion of vitamins are unstable during food processing and storage and exhibit a relatively low biopotency. Consequently, delivery carriers are often employed to enhance the dispersibility, stability, and bioactivity of vitamins.^[^ ^51^ ^]^ For instance, Ma et al. (2022) constructed a photothermally responsive system comprising lecithin‐polyethylene glycol (PEG)‐modified poly (lactic‐co‐glycolic acid) (PLGA) nanoparticles, which were used to deliver V_C_ and indocyanine green to tumors, thereby exerting anticancer effects.^[^ ^52^ ^]^

Minerals, which are inorganic salts, are composed of both macro elements (Ca, Na, K, Mg, P, S, and Cl) and trace elements (Fe, Cu, Zn, Se, Mo, Co, Cr, I, Mn, Ni, Si, Sn, V, F, and B).^[^ ^53^ ^]^ In nature, minerals play a variety of important roles in maintaining human health and wellbeing. These include strengthening bones, maintaining cellular osmotic pressure and acid–base balance, protecting muscles, and modulating nerve function.^[^ ^53^ ^]^ Additionally, minerals can be used as crosslinkers to hold the different constituents together in delivery systems. For example, calcium is frequently employed as a crosslinker to generate calcium alginate microgels, which are used to encapsulate a variety of bioactive substances, including nutrients, nutraceuticals, and probiotics.^[^ ^54^ ^]^

### 2.3 Other Bioactive Substances

In addition to the above bioactive compounds with relatively simple composition, there is a class of other bioactive substances, probiotics with complex substance composition, which are also commonly used for the production of functional foods. Probiotics are a class of living microorganisms (usually bacteria) that are capable of colonizing the human GIT and altering its microbial composition in a manner that provides health benefits.^[^ ^55^ ^]^ Administration of sufficient quantities of viable probiotics exhibits a diverse range of health benefits, including inhibition of pathogenic or harmful bacteria, maintenance of intestinal flora balance, alleviation of intestinal diseases (acute diarrhea, irritable bowel syndrome, inflammatory bowel disease, and colon cancer), improvement of neurogenic diseases (Alzheimer's disease, Parkinson's disease, depression), prevention of cardiovascular and cerebrovascular diseases, and regulation of organ functions through the gut‐brain/liver/lung axis.^[^ ^56^, ^57^, ^58^, ^59^ ^]^ Lactic acid bacteria, including *Lactobacillus*, *Bifidobacterium*, *Coccus* (*Enterococcus, Streptococcus, Pediococcus*), *Bacillus*, and *Saccharomyces*, are the most commonly utilized probiotic species in foods and dietary supplements (Table S1, Supporting Information). Recently, the development of engineered probiotics based on synthetic biology has emerged as a promising research hotspot. For instance, the i‐ROBOT, designed by Zou and co‐workers can noninvasively monitor and record inflammatory bowel disease occurrence and progression in real‐time.^[^ ^60^ ^]^ However, the viability of most probiotics is limited in the GIT due to the presence of gastric acids, bile salts, and various digestive enzymes.^[^ ^61^ ^]^ Consequently, delivery systems have been developed to improve probiotic viability, enhance their intestinal adhesion and colonization, and increase their biological activities.^[^ ^55^ ^]^

## 3 Biopotency Barriers of Bioactive Food Ingredients

Biopotency, also known as bioavailability, is typically defined as the proportion of ingested bioactive food ingredients that reach the target organs and tissues of an organism through the circulatory systems (blood and lymph).^[^ ^5^, ^11^, ^62^ ^]^ In the pharmaceutical fields, bioavailability is divided into two categories: absolute bioavailability (administration by intravenous pathways) and relative bioavailability (administration by other pathways).^[^ ^63^ ^]^ In the food fields, the main focus is on the relative bioavailability of orally administered bioactive substances. Based on the relative bioavailability, there are currently two well‐accepted classifications of nutraceuticals and pharmaceuticals: the biopharmaceutical classification scheme (BCS) proposed by Amidon et al. in 1995, and the nutraceutical bioavailability classification scheme (NuBACS) defined by McClements et al. in 2015.^[^ ^5^, ^64^ ^]^ According to these schemes, the bioavailability of orally administered bioactives is dependent on multiple factors.^[^ ^5^, ^11^ ^]^ Typically, it is important to identify the most significant rate‐limiting factors for a specific bioactive substance, and then adopt suitable delivery strategies and develop appropriate delivery systems to overcome them. In general, the factors that limit the biopotency of food ingredients can be divided into in vitro and in vivo barriers.

### 3.1 In Vitro Barriers

**Figure 2:**
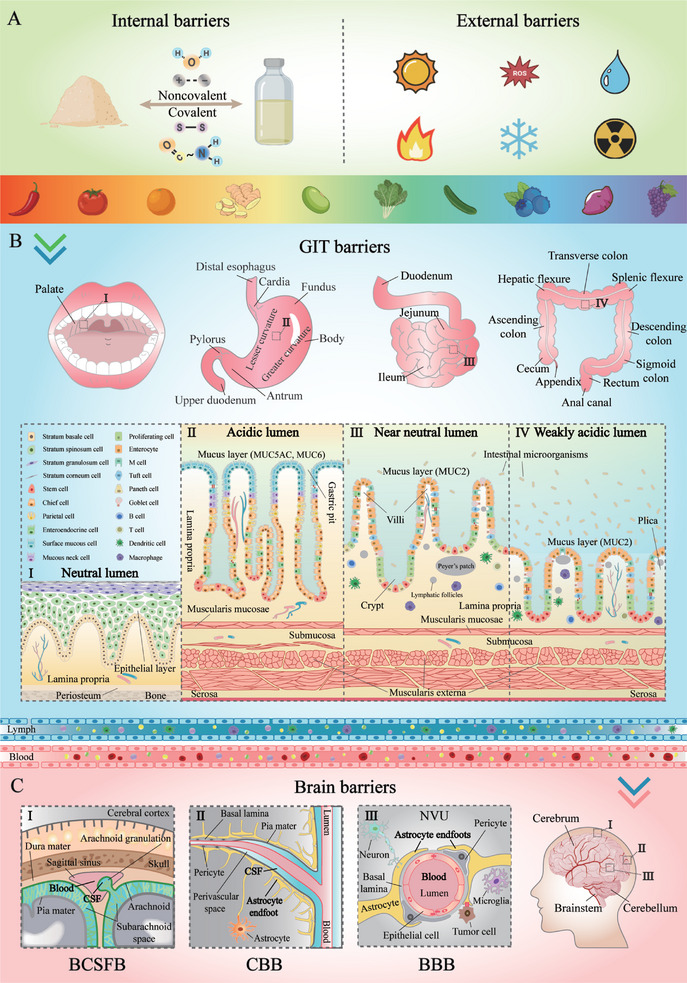
Biopotency barriers encountered by food bioactive ingredients. A) In vitro barriers. B) In vivo GIT barriers. C) In vivo brain barriers.

A number of factors can reduce the biopotency of bioactive food ingredients prior to ingestion, which are considered to be in vitro barriers (**Figure** **2**A). These factors are related to changes that occur in the ingredients during food processing, transport, and storage.^[^ ^63^ ^]^ These factors can be divided into internal and external barriers based on the observed effects.^[^ ^65^, ^66^, ^67^ ^]^

#### 3.1.1 Internal Barriers

Internal barriers depend on the nature of the bioactive food ingredient itself, rather than on its environment.

##### Inherent Properties

The inherent properties of bioactive food ingredients depend on their molecular structure, including their physical properties (e.g., physical state, rheology, and solubility), chemical properties (e.g., reactivity, acid–base properties, and redox potential), and biological properties (e.g., susceptibility to microbial growth, enzyme activity, and fermentation).^[^ ^34^, ^68^, ^69^, ^70^, ^71^, ^72^ ^]^ It is possible that the inherent properties of bioactive ingredients may impede their application or reduce their bioactivities. Many bioactive substances have low solubility in water, which presents a challenge in incorporating them into some foods and beverages.^[^ ^73^ ^]^ Additionally, some bioactive substances exhibit low permeability in the human body, which also reduces their bioactivity.^[^ ^74^ ^]^ For example, the low water solubility, low permeability, and short half‐life of andrographolide severely limit its bioactivities.^[^ ^75^ ^]^ Moreover, some bioactive substances are chemically or biologically unstable, which causes them to lose their biopotency prior to consumption. For instance, carotenoids are susceptible to chemical degradation during processing and storage due to the presence of chemically labile double bonds.^[^ ^76^ ^]^ Similarly, proteins may lose their enzymatic activity if they undergo conformational changes.^[^ ^77^ ^]^ Probiotics may also lose their health benefits if they become inactivated during storage and transport.^[^ ^78^ ^]^ However, several studies have demonstrated that inactivated probiotics may still exert some health benefits in humans, which are then known as “postbiotics”.^[^ ^79^, ^80^ ^]^

##### Component Interactions

The molecular properties of bioactive food ingredients may result in interactions between them and other substances in their environment, which may alter their applications or biological activities.^[^ ^63^, ^81^ ^]^ These interactions can be categorized into two main groups: non‐covalent and covalent. Non‐covalent interactions include hydrogen bonding, van der Waals’ forces, π–π stacking interactions, hydrophobic interactions, and electrostatic interactions. Covalent interactions are produced by chemical reactions (such as disulfide bond formation or the Maillard reaction) and enzymatic reactions (such as transglutaminase, laccase, or peroxidase catalyzed reactions).^[^ ^82^, ^83^, ^84^, ^85^, ^86^, ^87^ ^]^ For instance, two oppositely charged biopolymers may aggregate by electrostatic attraction, which adversely affects their ability to be incorporated into food or beverage matrices.^[^ ^88^ ^]^ The interaction of proteins with tannins can cause astringency, which reduces their palatability and affects the digestion and absorption of proteins, thereby decreasing the nutritional benefits.^[^ ^63^, ^83^ ^]^ The oxidation of V_C_ by transition metal ions may reduce its physiological benefits.^[^ ^89^ ^]^ Consequently, it is important to understand the potential of a bioactive ingredient to interact with other substances in its environment, as well as the impact of these interactions on its biological activities.

#### 3.1.2 External Barriers

The biopotency of bioactive food ingredients may also be adversely impacted by external factors.

##### Environmental Factors

The exposure of bioactive food ingredients to adverse external conditions during processing, transport, or storage (such as light, oxygen, or moisture) may also result in a reduction in their biopotency.^[^ ^90^ ^]^ Electromagnetic radiation, including ultraviolet (10–400 nm), visible (400–700 nm), and infrared (700–10^6^ nm) light, may cause damage to some bioactives.^[^ ^91^ ^]^ The wavelength, intensity, and duration of radiation exposure may impact the biopotency of photosensitive ingredients, such as polyphenols, terpenoids, and vitamins, through a series of light‐induced reactions.^[^ ^92^ ^]^ For instance, resveratrol naturally exists in two isomeric conformations (*trans*‐ and *cis*‐), with the *trans*‐isomer being more stable and dominant. However, *cis*‐isomers are formed from *trans*‐isomers when resveratrol is exposed to ultraviolet and visible radiation, which results in a decrease in its bioactivity.^[^ ^93^ ^]^ The presence of oxygen may promote the oxidation of many bioactive substances, including unsaturated fatty acids, carotenoids, polyphenols, terpenoids, and vitamins, by generating free radicals (e.g., •O_2_^−^, •OH, ROO•, RO•, R•, NO•, NO_2_•, or OONO^−^) or reactive oxygen species (e.g., ^1^O_2_, H_2_O_2_, or oxygen‐containing free radicals previously listed).^[^ ^94^, ^95^ ^]^ For example, lipid autoxidation occurs when oxygen initiates a series of free radical chain reactions that promote lipid oxidation, thereby reducing the biopotency of lipids.^[^ ^96^ ^]^ Similarly, high relative humidities can facilitate the physical or chemical transformations of some bioactive ingredients, as well as promote the proliferation of microorganisms, thereby altering the biological activity of these ingredients.^[^ ^92^ ^]^

##### Processing Treatments

Some processing technologies can also affect the activities of bioactive substances through physical, chemical, or biological effects.^[^ ^63^, ^97^ ^]^ The most common physical treatments include temperature changes (such as heating, chilling, or freezing), electromagnetic radiations (such as microwaves, X‐rays, gamma rays, or pulsed electric fields radiation), pressure changes (such as high‐pressure processing), sonications and so on.^[^ ^98^ ^]^ These treatments are typically divided into either thermal or non‐thermal processing technologies. Thermal processing technologies transfer heat into foods by conduction, convection, or radiation, which can result in undesirable changes in bioactive substances, such as denaturation, isomerization, or degradation.^[^ ^97^, ^98^ ^]^ Non‐thermal processing technologies can also promote the degradation of some bioactive substances, e.g., due to the application of electromagnetic radiation, pressure, or shear forces.^[^ ^99^ ^]^ Furthermore, chemical and biological processing technologies may also promote undesirable alterations in the solubility, stability, or activity of bioactives.^[^ ^83^, ^100^ ^]^ For example, extreme pH conditions and high sodium chloride (NaCl) concentrations can adversely affect the thermal stability of lactoferrin, thereby limiting its iron‐binding and brain‐targeting capabilities.^[^ ^101^ ^]^ In addition, exposure to strong alkaline conditions can cause polyphenols to oxidize, which reduces their bioactivities.^[^ ^83^ ^]^ Therefore, it is important to gain a deeper understanding of the impact of external barriers on the stability and efficacy of bioactive food ingredients, in order to design appropriate delivery systems to overcome these barriers.

### 3.2 In Vivo Barriers

Following oral administration, bioactive food ingredients enter the internal environment of the human body, where they may undergo a range of physiological processes and encounter various obstacles that limit their biological activities (in vivo barriers).^[^ ^102^ ^]^ This section highlights the various in vivo barriers that may reduce the potency of bioactives.

#### 3.2.1 Barriers in Digestive System

The ingestion, mastication, digestion, and absorption of foodstuffs depend on the digestive system of the human body and play a critical role in the bioavailability of bioactive food ingredients.^[^ ^103^ ^]^ In humans, the GIT is lined by an inner mucosal surface and has various accessory digestive glands regulated by the neural network and hormones. The GIT encompasses the upper digestive tract (oral cavity, pharynx, esophagus, stomach, duodenum, jejunum, and ileum) and the lower digestive tract (cecum, appendix, colon, rectum, anal canal, anus). GIT is a long muscular organ that mixes, disrupts, and propels foods and beverages by mechanical actions, including mastication, contraction/expansion, and peristalsis. A multitude of digestive glands exist in the GIT, including the large ones (parotid glands, submandibular glands, sublingual glands, pancreas, and liver) and the small ones (gastric glands and intestinal glands). These glands are capable of secreting digestive fluids that facilitate the physical, chemical, and enzymatic breakdown of food into smaller molecules, thereby enhancing their absorption.^[^ ^103^, ^104^, ^105^ ^]^ The various physiological and physicochemical processes that occur from the mouth to the anus impact the biopotency of bioactive food ingredients by affecting their digestion, transport, and absorption (Figure 2B).

##### Mouth

The oral cavity is the initial point of the digestive tract and, from an anatomical perspective, comprises the muscular layer, the submucosa, the lamina propria, and the stratified squamous epithelium layer. Furthermore, the oral cavity contains digestive glands, primarily salivary glands, such as the parotid, submandibular, and sublingual glands. It also contains additional organs, including the lips, teeth, palate, and tongue.^[^ ^106^, ^107^ ^]^ Following ingestion, foods (especially those in a solid or semi‐solid state) are subjected to the mechanical forces generated by teeth and tongue movements during mastication, the enzymes in saliva, the mucosa, and the flora of oral cavity.^[^ ^108^, ^109^ ^]^ The mechanical movements of teeth and tongue are coordinated by the central nervous system and various tissue‐organ receptors. These movements are commonly expressed as compressing, cutting, tearing, grinding, and crushing behaviors.^[^ ^104^, ^105^ ^]^ The mechanical forces generated by these movements play pivotal roles in the digestive process: 1) reducing the dimensions of solid and semi‐solid foodstuffs into an appropriate range (0.8–3.0 mm) that is suitable for swallowing; 2) increasing the surface area of foodstuffs to enhance their contact and reactions with digestive juices; 3) disrupting food matrices to promote bioactive release; 4) altering the rheological properties of foods to facilitate their swallowing.^[^ ^103^, ^108^, ^110^ ^]^ Saliva in the mouth is a viscous fluid secreted by salivary glands, with a pH range of 5.0–7.0. The composition of saliva is complex, including water (> 99.5%), glycoproteins (mucin), enzymes (α‐amylase, lingual lipase), carbohydrates, glycolipids, and electrolytes.^[^ ^104^, ^105^, ^106^ ^]^ The digestive enzyme α‐amylase in saliva hydrolyzes starch by breaking α‐1,4 glycosidic bonds, but may also retain volatile flavor molecules by binding them.^[^ ^109^ ^]^ The release of bioactive food ingredients in the human GIT can be modulated by controlling the breakdown of functional foods or delivery systems in the mouth, which can be achieved by altering their composition, structural organization, and mechanical properties.^[^ ^108^ ^]^

##### Stomach

Once swallowed, boluses of solid or liquid foods are transported to the stomach through the esophagus, which is lined with friction‐resistant epithelial cells. The esophagus is ≈25 cm in length and 1.5–2.0 cm in diameter.^[^ ^105^ ^]^ The bolus then passes through the esophageal sphincter and enters the stomach. The stomach is a highly deformable, crescent‐shaped muscular pouch comprising various parts: cardia, fundus, body, antrum, pylorus, and pyloric sphincter.^[^ ^104^, ^111^ ^]^ The components of the stomach are designed to prevent food reflux, preserve foods and swallowed air, and facilitate food digestion and gastric emptying.^[^ ^112^ ^]^ Figure 2B illustrates the anatomy of the stomach, which is comprised of the serosa, muscularis externa (outermost longitudinal muscle layer, middle circular muscle layer, innermost oblique muscle layer), submucosa (vessels, loose connective tissue), mucosa (muscularis mucosae, lamina propria, capillaries, simple columnar epithelium), and lumen (gastric fluids). The muscular layers of the stomach are regulated by the nervous system and various hormones, which facilitate the generation of different kinds of contractions that promote food mixing and grinding.^[^ ^104^, ^105^ ^]^ The surface of the gastric epithelium layer contains numerous pits with glands at the bottom. The epithelium layer is primarily composed of six different functional cells: 1) stem cells – basal cells with differentiation function; 2) chief cells – cells containing large numbers of rough endoplasmic reticulum, Golgi complex, and enzyme granules to secrete pepsinogen and gastric lipase; 3) enteroendocrine cells – cells that secrete hormones such as gastrin, histamine, serotonin, endorphins, and cholecystokinin into the blood; 4) parietal cells – cells containing large numbers of mitochondria, secretory tubules, and microtubule systems to secrete hydrochloric acids (gastric acids) that inhibit bacteria, activate pepsinogen, and promote vitamin B_12_ absorption by intrinsic factors; 5) surface mucous cells – cells that secrete alkaline fluids containing mucin to form a mucus layer to protect the epithelium cells from gastric acids and other substances in the stomach; 6) mucous neck cells – cells that secrete acidic fluids containing mucin to protect the mucus layer.^[^ ^105^, ^113^ ^]^ The elastic stomach lumen (25–50 mL volume in the fasting state) contains gastric juices (2.0–3.0 L d^−1^, pH 1.0–3.0), which consist of water, hydrochloric acid, electrolytes, gastric gland cell secretions, and residual oral salivary gland secretions. These juices can corrode, soften, dilute, dissolve, or disintegrate foods, leading to the formation of chyme.^[^ ^104^, ^112^ ^]^ Typically, these processes break down foods to a small size (<1.0–2.0 mm), facilitating their passage through the pylorus sphincter during gastric emptying. Although the absorption of bioactive food ingredients in the stomach is usually limited, the gastric phase is of importance for disrupting the food matrix, which facilitates the subsequent release of the bioactives in the small intestine. Furthermore, the high acidity of gastric juices, the movements of muscular layer, the enzymes, mucosa and flora in the stomach, can alter the stability and activity of some bioactive food ingredients, such as proteins and carotenoids. Consequently, it is important to consider the gastric conditions when designing delivery systems for bioactive compounds.

##### Small Intestine

The small intestine, comprising the duodenum (25–30 cm), jejunum (1.0–2.5 m), and ileum (1.5–3.5 m), is the longest section of the human digestive tract. It digests and absorbs nutrients and bioactive agents, which are regulated by accessory organs, such as the pancreas, liver, and gallbladder.^[^ ^104^, ^105^, ^108^ ^]^ The small intestine consists of several layers (from outer to inner): the mesentery, visceral membrane (serosa, subserosa), muscularis externa (exterior longitudinal muscular layer, interior annular muscular layer), submucosa (blood vessels, lymphatic vessels, loose connective tissue), mucosa (muscularis mucosae, lamina propria, capillaries, central lacteal, small intestinal villi), and intestinal lumen (diameter 3–4 cm).^[^ ^105^, ^113^ ^]^ The small intestine exhibits rhythmic contractions and peristalsis movements, which are regulated by nerves, hormones, and muscles: 1) contraction increases the contact between chyme and villi, thereby promoting absorption; 2) peristalsis propels the chyme through the intestine at a rate of ≈2–2.5 cm s^−1^.^[^ ^104^, ^105^ ^]^ In the mucosal layer, the small intestinal villi are bounded by a monolayer of columnar epithelium cells, forming “villi‐crypt” structures. The intestinal stem cells located at the base of these crypts differentiate into a variety of functional cells, including enterocytes, which are responsible for nutrient absorption; microfold cells (M cells), which present antigens from the lumen to the lymphatic follicles; tuft cells, which mediate immune responses; Paneth cells, which secrete antimicrobial compounds, ligands, and cell differentiation factors; goblet cells, which secrete mucus; and enteroendocrine cells, which secrete hormones.^[^ ^102^, ^114^, ^115^ ^]^ In addition, the mucosal layer is protected by the mucosal immune system, which comprises a variety of immune cells (B cells, T cells, dendritic cells, monocytes/macrophages, mast cells, M cells), cytokines (antimicrobial peptides, lysozymes, defensin) and cell receptors (immunoglobulins) that are present in lymph nodes, the lamina propria, and epithelial cells.^[^ ^116^ ^]^ The intestinal lumen is lined with a mucus layer (thickness 10–200 µm) that covers and protects the epithelial cells. Mucus is a negatively charged viscous fluid that is composed of mucin (mainly mucin 2, abbreviated as MUC2), sialic acids, electrolytes, water, and other components. It can be divided into an inner layer of high viscosity and an outer layer of low viscosity.^[^ ^117^, ^118^ ^]^ Furthermore, the small intestinal fluids in the lumen (pH 6.6–7.5) contain electrolytes, microorganisms, enzymes, bile acids, and hormones dissolved or dispersed in water.^[^ ^105^, ^113^ ^]^

##### Large Intestine

The large intestine is shorter (≈1.5 m) but wider (5–7 cm in diameter) compared to the small intestine. The large intestine is comprised of an M‐shaped tube, including the cecum (6–7 cm), appendix, ascending colon (15–20 cm), transverse colon (45–50 cm), descending colon (25–30 cm), sigmoid colon (40 cm), rectum (12 cm), anal canal, and anus.^[^ ^119^, ^120^ ^]^ The anatomical structure of the large intestine wall is similar to that of the small intestine, however, there are three main differences. First, the large intestine is responsible for the transportation of digested foods from the colon to the rectum. This is achieved through the use of contraction waves (“peristalsis”), as well as reverse movements in the proximal large intestine through the action of gravity or reflux waves. The velocity at which digested materials move through the large intestine is influenced by the health status of the human body, such as the presence of diarrhea.^[^ ^105^, ^120^ ^]^ Second, the epithelial cells form plicae with small crypts rather than villi, and have a reduced number of functional cells. For instance, Paneth cells are rarely present in the large intestine, and some of their secretory functions are performed by deep secretory cells.^[^ ^115^ ^]^ Third, there are numerous microorganisms (≈1.5 kg) in the large intestinal lumen.^[^ ^113^ ^]^ These microorganisms ferment the foods that reach the large intestine. The presence of *Actinobacteria, Bacteroidetes, and Firmicutes* can stimulate bowel peristalsis, promote the uptake of water, salts, and degraded cellulose, generate gases, and produce shortchain fatty acids (SCFAs).^[^ ^108^, ^113^ ^]^ The presence of SCFAs lowers the intestinal pH and impacts other organs through the gut‐brain and gut‐liver axes. Bioactive molecules that reach the large intestine may be absorbed into the intestinal capillaries and then pass through the portal vein and on to the liver. They may then be distributed to tissues and organs via the circulatory systems or be cleared through liver‐gallbladder synergetic metabolism (first‐pass elimination effect).

In conclusion, the biopotency of ingested bioactive food ingredients may be influenced by various factors within the GIT, including mechanical barriers associated with the movements of the gastrointestinal muscular layer; metabolic barriers associated with enzyme‐catalyzed reactions due to the digestive and metabolic enzymes released by the human body and microorganisms, as well as associated with circulatory systems in the human body; acid–base barriers associated with foods, digestive juices or special pathological microenvironment; mucosal barriers associated with transport across the mucus layer; physical barriers, including cellular and organelle barriers, are associated with the transport across epithelial cells, intercellular tight junctions, and organelles; immune barriers associated with the mucosal immune system; and microbiological barriers associated with the intestinal flora. It is crucial to have an understanding of the physicochemical and physiological processes occurring within the GIT in order to develop more efficacious delivery vehicles for bioactive compounds.

#### 3.2.2 Barriers in Targeted Sites

Once bioactive food ingredients or their metabolites are transported from the digestive tract into the circulation systems, they may encounter various barriers before being absorbed by tissues or organs. For example, the blood circulatory system, immune system, tissues, and organs contain a variety of metabolic enzymes that may alter the structure and activity of bioactive ingredients. The uptake of bioactive molecules is influenced by transcellular or paracellular pathways in tissues and organs, such as the liver, heart, kidneys, muscles, eyes, and brain, which may selectively exclude or absorb the bioactive molecules.^[^ ^121^, ^122^, ^123^ ^]^ As shown in Figure 2C, the brain, as the most sophisticated and fragile organ in the human body, has evolved multiple barriers to protect it, including the blood‐cerebrospinal fluid barrier (BCSFB), the cerebrospinal fluid‐brain barrier (CBB), and the blood–brain barrier (BBB).^[^ ^124^ ^]^ These barriers, especially the BBB, are the main obstacles for bioactives to alleviate some neurological disorders.

## 4 Delivery Strategies to Overcome In Vitro Barriers

The efficacy of bioactives is often limited by changes in their physicochemical properties prior to ingestion, which may be due to the internal or external barriers mentioned earlier (Section 3.1). These challenges can often be overcome by encapsulating the bioactives in well‐designed delivery carriers based on appropriate delivery strategies (Table S2, Supporting Information).

### 4.1 Strategies for Internal Barriers

Internal barriers to the efficacy of bioactive food ingredients prior to ingestion include low solubility, low permeability, poor stability, and adverse interactions with other substances. The delivery strategies for overcoming internal barriers are based on a comprehensive understanding of the physicochemical properties of the bioactive agents and the materials used to construct the delivery systems. It is important to strike a balance between the enhancement of physicochemical properties and the maintenance of bioactivities. For instance, if the delivery system is excessively stable, it may not only protect the encapsulated bioactives, but may also impede their release in the GIT.

#### 4.1.1 Enhancement of Solubility

**Figure 3:**
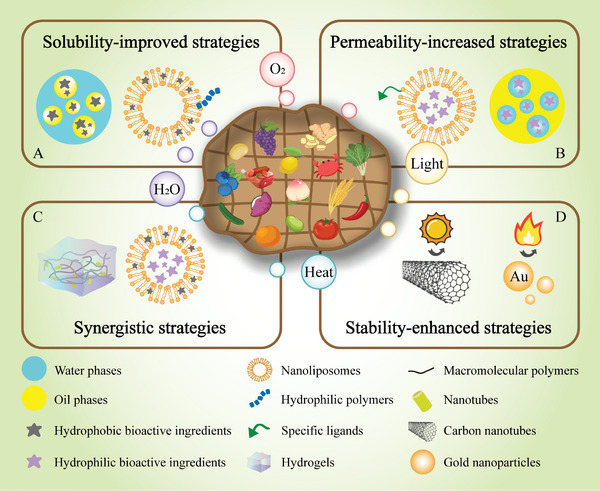
Main strategies and common delivery systems for overcoming in vitro barriers. A) Oil in water emulsion and hydrophilic polymer‐modified nanoliposome for improving the solubility of hydrophobic food bioactive ingredients. B) Water in oil emulsion and specific ligand‐modified nanoliposome for increasing the permeability of hydrophilic food bioactive ingredients. C) Nanotubes in hydrogel for achieving the synergy of different carriers, multi‐compartmental nanoliposome for achieving the synergy of different bioactive cores. D) Stability‐enhanced carriers based on inorganic and organic materials for resisting environmental and processing conditions.

Water‐soluble or water‐dispersible bioactives can be easily dissolved or dispersed into aqueous food matrices. In contrast, lipid‐soluble bioactives (such as carotenoids and some vitamins) usually need to be encapsulated before they can be dispersed in aqueous food matrices. For instance, they can be incorporated into the lipid droplets in oil‐in‐water microemulsions, nanoemulsions or emulsions, into the biopolymer particles in microgels or protein nanoparticles, or into the hydrophobic interior of cyclodextrins or surfactant micelles (**Figure** **3**A). Cyclodextrins (CDs), including α‐, β‐ and γ‐CDs, are cyclic oligosaccharides with natural hollow conical structures with internal hydrophobic and external hydrophilic regions.^[^ ^125^ ^]^ This unique structure allows hydrophobic bioactives to form host–guest complexes with CDs, thereby improving their water solubility. For instance, the encapsulation of hydrophobic cannabidiols in β‐CDs has been shown to significantly improve their water solubility.^[^ ^126^ ^]^ Similarly, cyclodextrins have been used to encapsulate ferulic acids.^[^ ^127^ ^]^

#### 4.1.2 Enhancement of Permeability

It is generally observed that lipophilic and small molecular substances have favorable cell permeability and absorption efficiencies. The bioactives with poor permeability typically require encapsulation technologies to achieve enhanced permeability. For example, they can be incorporated into delivery systems, such as water‐in‐oil emulsions, liposomes, exosomes, or biopolymer particles, whose composition, size, shape, charge, and surface property can be tailored for specific applications (Figure 3B). Liposomes are self‐assembled vesicles with high cell permeability and biocompatibility due to their bilayer membrane structure.^[^ ^36^ ^]^ Baek et al. (2023) prepared nanoliposomes with a diameter of less than 100 nm using the thin‐lipid film hydration method, which was found to be effective in improving the solubility and permeability of encapsulated resveratrol.^[^ ^128^ ^]^ The permeability of collagen peptides has also been increased by encapsulating them in alginate‐coated liposomes.^[^ ^129^ ^]^

#### 4.1.3 Enhancement of Stability

The presence of unsaturated bonds in the structure makes many bioactive substances susceptible to degradation during food processing, transport, and storage. The degradation reduces the biopotency of these substances. For instance, they may be physically or chemically degraded when exposed to heat, light, oxygen, moisture, chemicals, enzymes, or microbes. It is important to carefully select the most appropriate materials to assemble bioactive delivery systems. The selection of materials that are inert and capable of encapsulating sensitive bioactives is often of great significance. For instance, it may be effective to incorporate antioxidants or to use wall materials that form barriers to pro‐oxidants for improving the oxidative stability of bioactives.^[^ ^130^ ^]^ Other researchers have used a combination of an acid‐stable polymer (Eudragit S100) and heat‐stable liposomes to improve the acid and thermal stability of co‐encapsulated lipophilic and hydrophilic bioactives.^[^ ^131^ ^]^ The selection of an appropriate encapsulation technology is also crucial for improving the physicochemical stability of these bioactive substances under external stimuli, which will be discussed in more detail in Section 4.2.

#### 4.1.4 Suppression of Adverse Interactions

The efficacy of certain bioactive food ingredients may be adversely impacted by their interactions with other substances in their surroundings. For instance, transition metals may facilitate the oxidation of polyunsaturated lipids, proteases may degrade bioactive proteins, and alkalis may promote the degradation of polyphenols. Consequently, it is of great importance to isolate these bioactives from any reactive substances in their environment. Once more, this can be achieved by encapsulating the bioactive compounds in appropriate delivery systems, as well as using synergistic strategies (Figure 3C). For example, the bilayer structure of liposomes provides both polar and non‐polar internal domains, which can co‐deliver hydrophilic and hydrophobic bioactives, such as anthocyanidin and docosahexaenoic acid (DHA).^[^ ^132^ ^]^ Furthermore, researchers have constructed a synergistic delivery carrier by using intestinal adhesive low‐methoxy pectin and intestinal permeable α‐lactalbumin nanotubes to co‐deliver capsaicin.^[^ ^133^ ^]^

### 4.2 Strategies for External Barriers

Prior to ingestion, bioactive food ingredients are subject to a number of external barriers that can affect their potency. These barriers include exposure to changes in environmental conditions or food processing operations that cause degradation, which can greatly affect the stability of bioactive substances and the efficacy of delivery carriers. In the design of delivery strategies for external barriers, it is essential to identify the specific environmental factors and processing conditions that cause instability. This identification can then be used to optimize the environmental and processing conditions. Similarly, a balance should be struck between quality enhancement and functionality maintenance, as discussed previously.

#### 4.2.1 Protection Against Environmental Stimuli

Foods may be exposed to various kinds of environmental conditions before they are consumed, including alterations in light, oxygen, temperature, and moisture content. The resistance of bioactives to changes in these environmental conditions can frequently be increased by encapsulating them within delivery carriers (Figure 3D).^[^ ^134^ ^]^ As an example, the encapsulation of curcumin in γ‐CD metal–organic frameworks (MOFs) carriers has been demonstrated to retard its degradation when exposed to ultraviolet light.^[^ ^135^ ^]^ The use of yeast cell microcarriers has been demonstrated to protect encapsulated krill oil from oxidation during storage.^[^ ^136^ ^]^ Delivery systems formulated from a combination of wheat dietary fibers and soy proteins have been shown to reduce the loss of potassium iodide (KI) and potassium iodate (KIO_3_) in high relative humidity (90%) environments.^[^ ^137^ ^]^

The stability of delivery systems depends on the molecular and colloidal interactions between them, encompassing both attractive (mainly van der Waals forces, hydrogen bonding, and hydrophobic interactions) and repulsive (mainly electrostatic and steric interactions) interactions. When the attractive interactions predominate over the repulsive ones, the delivery systems are typically prone to precipitation and separation. Consequently, it is of great importance to regulate the molecular or colloidal interactions in delivery systems by selecting appropriate materials to construct them, controlling their size, charge, and surface hydrophobicity, and controlling solution conditions such as pH and ionic strength. Precipitation can be prevented by ensuring that the system particles are small, highly charged, and have few exposed non‐polar surface groups. Furthermore, the stability of delivery systems can be enhanced by coating them with emulsifiers or polymers that increase the steric repulsion between them. For instance, Cao et al. (2023) prepared nanoparticles comprising soy proteins, hydroxypropyl methylcelluloses, and broccoli leaf polyphenols, which they then used to stabilize Pickering emulsions, thereby achieving emulsions with high stability and dispersibility.^[^ ^138^ ^]^ Ong et al. (2020) demonstrated that the stability of iron oxide nanoparticles could be enhanced by coating them with fatty acids.^[^ ^139^ ^]^ In addition, cationic inulin coatings have been shown to improve the stability of liposomes, as well as the light and oxidative stabilities of co‐encapsulated betanin and carvone.^[^ ^140^ ^]^

#### 4.2.2 Protection Against Processing Interferences

A variety of processing operations may be applied to foodstuffs before they are consumed. These operations include mechanical stresses, thermal processing, high‐pressure processing, radiation treatments, and chemical treatments. It is therefore essential to design delivery carriers for withstanding all the harsh conditions that a bioactive‐enriched food may experience during production, storage, and transport. For example, nano‐silicate‐enhanced κ‐carrageenan hydrogels exhibit strong shear‐thinning properties, which are advantageous for achieving certain commercial applications.^[^ ^141^ ^]^ The encapsulation of Fe^2+^ within alginate‐starch beads has been shown to enhance their resistance to oxidation during thermal processing at 180°C.^[^ ^142^ ^]^ Furthermore, liposomes coated with chitosan and gold nano‐shells have been shown to improve the photothermal stability of resveratrol.^[^ ^143^ ^]^ In another study, β‐CD nano‐sponge complexes and gold nanoparticles have been shown to enhance the stability and photothermal conversion ability of curcumin.^[^ ^144^ ^]^ Other researchers have developed zein‐β‐glucan nanoparticles that demonstrated good resistance to pH changes.^[^ ^145^ ^]^ High‐pressure processing is often employed to improve the stability of delivery systems (especially emulsions) by altering the microstructure of the systems.^[^ ^146^ ^]^ However, the intensity and duration of the high‐pressure treatment must be carefully controlled.

## 5 Delivery Strategies to Overcome In Vivo Barriers

Following ingestion, bioactive food ingredients are subject to a series of barriers within the human body, which may result in a reduction in their biopotency. Consequently, it is important to adopt appropriate delivery strategies for overcoming these barriers. Some of the barriers within the GIT are similar to those encountered in foods prior to ingestion, such as low solubility, low permeability, poor stability, or adverse interactions with other components. Similar strategies can be used to overcome these barriers. However, there are also various other barriers that require the design of delivery systems to overcome them, which are considered in this section.

### 5.1 Acid‐Stable and ‐Targeted Strategies

**Figure 4:**
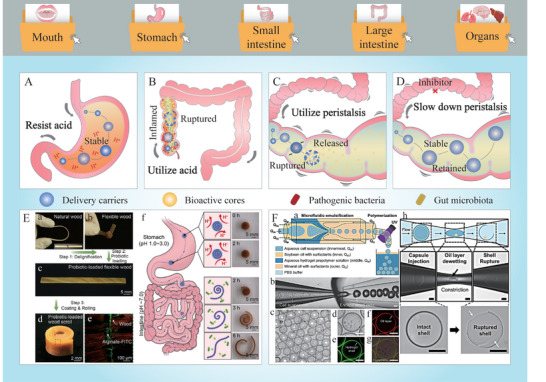
Main strategies for overcoming the acid–base barriers and the mechanical barriers in oral delivery. A) Delivery systems that can resist acidic conditions and keep stable in the stomach. B) Delivery systems that can utilize acidic conditions and responsively release in the inflamed colon. C) Delivery systems that can utilize gastrointestinal peristalsis to promote delivery carriers rupture and bioactive cores release. D) Delivery systems that can maintain stability along with slowed gastrointestinal peristalsis. E) Fabrication, structural characterization, and release performance of a gastric acid‐resistant and probiotic‐loaded wood scroll (Reproduced under terms of the CC‐BY license.^[^ ^54^ ^]^ Copyright 2022, American Chemical Society). F) Microfluidic preparation of stem cell‐loaded hydrogel microcapsules that can be segmented during peristalsis and constriction movements (Reproduced with permission.^[^ ^156^ ^]^ Copyright 2022, Elsevier).

To support a range of normal physiological activities, the human body generally maintains acid–base homeostasis (pH 7.2–7.4) under the regulation of various organs, tissues, and systems.^[^ ^147^ ^]^ However, several special regions, including the stomach (pH 1.0–3.0), the intestines (pH 5.5–7.5), the lesion areas (pH 3.0–6.8), and some organelles (pH 4.0–8.0) have different pH conditions, often being more acidic.^[^ ^147^, ^148^, ^149^, ^150^ ^]^ Some bioactives are susceptible to degradation under highly acidic conditions, necessitating the use of delivery carriers to protect them from the acidic gastric environment (**Figure** **4**A). Carriers composed of different food ingredients have been found to be acid‐resistant, including chitosan, alginate, hyaluronic acid, pectin, and casein.^[^ ^151^ ^]^ These components can form protective coatings or matrices around the bioactives, protecting them from the surrounding acids.^[^ ^92^ ^]^ For example, calcium alginate has been used to protect bioactives in the stomach but release them in the small intestine (Figure 4E).^[^ ^54^ ^]^

The acidic environments within the human body can be exploited to design delivery systems with acid‐responsive release properties (**Table** **1**). Regions of the intestinal tract where microbial activity is prevalent, particularly the colon and cecum, or regions that have been damaged by inflammation, tend to have a low pH (Figure 4B).^[^ ^152^, ^153^ ^]^ Consequently, an acid‐responsive strategy can be used to deliver bioactives to these regions. For instance, curcumin‐loaded core‐shell nanoparticles with pH‐sensitive polymer coatings (Eudragit EPO and L100) have been developed.^[^ ^149^ ^]^ These two coatings could dissolve sequentially in the small intestine and colon, thereby releasing curcumin at inflammatory sites associated with ulcerative colitis. In another study, researchers took advantage of the acidic tumor microenvironment to design pH‐triggered anti‐cancer nanoparticles.^[^ ^154^ ^]^

**Table 1:** Main strategies and common delivery systems for overcoming in vivo barriers of food bioactive ingredients.

Targeted barriers	Type of strategies	Preparation methods	Wall materials	Core materials	Refs
*Acids*					
	Utilizing acids: Nanoparticles	Self‐assembly	Eudragit EPO Eudragit L100	Curcumin	[149]
	Resisting acids: Wood scrolls	Softening Coating	Coating: sodium alginate Crosslinker: Ca^2+^	*Lactobacillus plantarum* Tea polyphenol Rapeseed oil	[54]
*Mechanical movements*					
	Utilizing movements: Hydrogel microcapsules	Microfluidic emulsification	Outer layer: mineral oil Middle layer: prepolymer Inner layer: soybean oil	Mesenchymal stem cells	[156]
	Resisting movements: Coated probiotics	Self‐assembly Coating	Tannic acid Poloxamer 188	*Escherichia coli* Nissle 1917	[162]
*Enzymes*					
	Enzyme immobilized: Porous microspheres	Modification Coating	Chitosan Crosslinker: glutaraldehyde Porogen: stearic acid	Inulinase	[165]
	Enzyme modified: Nanoparticles	Chemical modification Nanoprecipitation Self‐assembly	PLGA‐PEG Modification: rHuPH20	Doxorubicin	[166]
	Enzyme responsive: Micelles	Self‐assembly	Hydroxyethyl starch Curcumin	Dexamethasone	[171]
*Circulatory systems*					
	Short‐circulating: Nanofibers	Self‐assembly	Peptide	Doxorubicin	[174]
	Long‐circulating: Micelles	Self‐assembly	Zein PSBMA	Curcumin	[176]
*Mucus layer*					
	Mucoadhesive (noncovalent): Nanoparticles	Physically blending	Chitosan Dextran sulfate sodium salt	Coenzyme Q10	[183]
	Mucoadhesive (covalent): Micelles	Chemical modification Self‐assembly	Hyaluronic acid Modification: *N*‐acetylcysteine	Paclitaxel	[185]
	Mucus‐penetrating (nondestructive): Nanoparticles	Miscible organic solvent method	Silk fibroin Modification: Pluronic F‐127	Resveratrol	[187]
	Mucus‐penetrating (destructive): O/W emulsions	Hydrophobic ion pairing Self‐emulsifying	External phase: water Surfactants: SDS, ST, SDO, Kolliphor EL Co‐surfactant: propylene glycol Internal phase: Captex 300 Modification: trypsin	–	[189]
*Cells*					
*Cell membrane	Transmembrane (nondestructive): Nanoparticles	Chemical modification Self‐assembly	Glycogen Modifications: urocanic acid, α‐lipoic acid	Ginsenoside Rh2	[194]
	Transmembrane (nondestructive): Nanoparticles	Chemical modification Solvent evaporation	Hyaluronic acid Linker: cystamine dihydrochloride Modification: stearic acid	Procyanidins Modification: TPP	[195]
	Transmembrane (nondestructive): Nanofibers	Electrospinning Coating	PCL, poly‐D‐lysine Modification: β cells membrane derived vesicle	–	[197]
	Transmembrane (destructive): Nanoparticles	Chemical synthesis	Arginine‐rich polymer	–	[198]
	Transmembrane (destructive): Nanoparticles	–	Oxidized carbon nanoparticle	Nucleic acids	[199]
	Paracellular (irreversible): Nanocarriers	Chemical modification	Pluronic‐based polymer Modifications: chitosan, ZOT‐derived tight junction opening peptide	Insulin	[205]
	Paracellular (reversible): Nanotubes	Chemical modulation Self‐assembly	α‐lactalbumin peptide	Mangiferin	[206]
*Cell transport	Transferred by enterocytes: Liposomes	Thin‐film hydration Chemical modification Coating	Liposome: soybean phospholipid, DSPE‐mPEG2000, cholesterol Modification: quercetin Coating: glycocholic acid‐chitosan oligosaccharide conjugate	Paclitaxel	[208]
	Presented by immune cells: Nanoparticles	Chemical modification Self‐assembly	β‐glucan Linker: 2,2’‐dithiolethanol	Doxorubicin Temozolomide	[209]
*Organelles	Organelles escape: Nanoparticles	Chemical synthesis Electrostatic complexation Self‐assembly	Polylactic acid, polyethyleneimine Modifications: hyaluronic acid, inulin	Paclitaxel	[9]
	Cytoplasmic escape: Nanoparticles	Chemical modification Solvent evaporation	Hyaluronic acid Linker: cystamine dihydrochloride Modification: stearic acid	Procyanidins Modification: TPP	[195]
*Gut flora*					
*Gut environment	Utilizing gut flora (pH): Nanotubes	Self‐assembly Chemical crosslinking	α‐lactalbumin peptide Modification: low‐methoxy pectin	Capsaicin	[133]
	Utilizing gut flora (Enzymes): Cavity carriers	Physical entrapment	*Spirulina platensis*	Curcumin	[218]
*Flora composition	Modulating gut flora (Probiotics): Wood scrolls	Simple co‐incubation	Flexible wood membrane	*Lactobacillus plantarum*	[54]
	Modulating gut flora (Prebiotics): Nanoparticles	Chemical synthesis Electrostatic complexation Self‐assembly	Polylactic acid, polyethyleneimine Modifications: hyaluronic acid, inulin	Paclitaxel	[9]
	Modulating gut flora (Postbiotics): Microcapsules	Microfluidic electrospray	Sodium alginate, resistant starch, chitosan	Indole‐3‐propionic acid	[224]
	Modulating gut flora (Synbiotics): Coated probiotics	Chemical crosslinking	Carboxymethylated β‐glucan Linker: tannic acid, Fe^3+^	*Escherichia coli* Nissle 1917	[223]
*Organs*					
*Receptive organs	Oral administration: Nanocomplexes	Ionic gelation	Chitosan, sodium alginate, oleic acid	Lutein	[10]
*Visceral organs	Oral administration: Nanoconjugates	Chemical modification	β‐glucan Modification: taurocholic acid	eGFP encoded plasmid	[233]
*Brain	Through targeting core: Solid dispersions	Solvent evaporation	Ethylcellulose, hydroxy‐propyl methylcellulose	Gastrodin Borneol	[234]
	Through targeting wall: Nanoclusters	Chemical modification Spontaneous aggregation	Selenium nanoparticle Modification: brain‐targeting peptide	Chlorogenic acid	[235]
	Through immune system: Coated nanoparticles	Spontaneous deposition	Quantum dot, iron oxide nanoparticle, assembled organic fluorescent nanoparticle Coating: yeast capsule	Indomethacin Paclitaxel	[236]
	Through gut‐brain axis: Microspheres	Simple co‐incubation	Sodium alginate	Three engineered *L. lactis* strains probiotics	[237]
	Through external physical stimuli: Magnetic nanoparticles	Hydrothermal synthesis	(carboxymethyl)‐stevioside FeCl_2_·4H_2_O	–	[238]
	Through pathological environment: Nanoparticles	Chemical synthesis Self‐assembly	PLGA‐PEG Mannose	Fingolimod	[239]

PLGA‐PEG: poly(lactic‐co‐glycolic acid)‐b‐poly (ethylene) glycol; rHuPH20: recombinant human hyaluronidase PH20; PSBMA: poly(sulfobetaine methacrylate); Pluronic F‐127: poloxamer 407 or polyethylene‐polypropylene glycol; SDS: sodium dodecyl sulfate; ST: sodium taurocholate; SDO: sodium deoxycholate; Kolliphor EL:polyoxyl‐35 castor oil; Captex 300: glyceryl tricaprylate/tricaprate; TPP: (5‐carboxypentyl) (triphenyl) phosphonium bromide; PCL: polycaprolactone; ZOT: zonula occludins toxin; DSPE‐mPEG2000: 1,2‐distearoyl‐sn‐glycero‐3‐phosphoethanolamine‐N‐[methoxy(polyethylene glycol)‐2000]; eGFP: enhanced green fluorescent protein.

Therefore, it can be concluded that an acidic environment is both a critical barrier to overcome and a necessary condition for the pH‐responsive release of oral delivery systems. The selection of appropriate delivery strategies should be based on an assessment of the actual requirements.

### 5.2 Mechanical‐Stable and ‐Targeted Strategies

Once within the digestive tract, bioactives and their delivery carriers are subjected to a range of mechanical stresses, including chewing, swallowing, and gastrointestinal motility (Table 1). In certain instances, it is crucial to develop bioactive‐loaded delivery vehicles that are resistant to these mechanical stresses. Conversely, in other scenarios, the delivery systems can be designed to release their bioactives in response to specific mechanical stresses. The processes of chewing and swallowing are of primary importance in the design of oral delivery systems. If a bioactive is to be released in the mouth, such as a flavor substance or a nutraceutical agent for oral diseases, then the delivery system should be designed to degrade and/or release the bioactive within the oral cavity during mastication.^[^ ^54^, ^155^ ^]^ Gastrointestinal motility plays a vital role in the digestion of foods and the release of bioactive agents (Figure 4C,D). The mechanical forces generated by GIT motility can be used to disrupt carriers, thereby facilitating the release of their bioactive agents. For example, Kim et al. (2022) encapsulated mesenchymal stem cells within hydrogel microcapsules, which were ruptured by gastrointestinal peristalsis, thereby releasing the loaded cells to inflamed tissues and repairing the intestinal barriers (Figure 4F).^[^ ^156^ ^]^ Alternatively, substances that inhibit GIT motility can be incorporated into foods to increase their retention time, thereby increasing the bioavailability of encapsulated bioactives.^[^ ^157^, ^158^, ^159^, ^160^ ^]^ For example, some surfactants can slow down the gastrointestinal peristalsis, thereby prolonging the retention time of encapsulated bioactive ingredients within the GIT and improving their absorption.^[^ ^161^ ^]^ Other researchers have demonstrated that the gastrointestinal stability of model probiotics (*Escherichia coli* Nissle 1917) can be improved by coating them with tannic acid and surfactants, which facilitate their intestinal colonization.^[^ ^162^ ^]^ It should be noted that the use of gastrointestinal motility enhancers and inhibitors should be carefully controlled to avoid adverse side effects.

### 5.3 Enzyme‐Related Strategies

**Figure 5:**
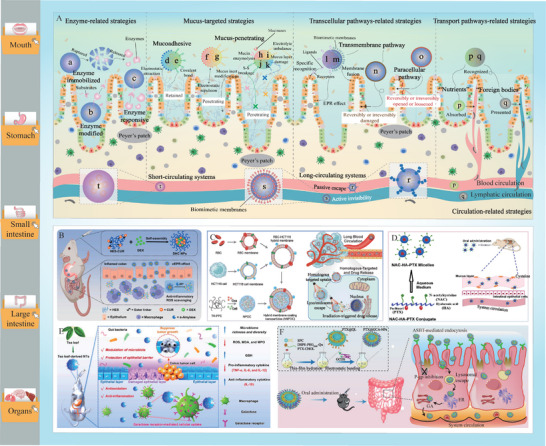
Main strategies for overcoming the metabolic barriers, the mucosal barriers, and the cellular barriers in oral delivery. A) Delivery systems that associated with enzymes (a–c), mucus layer (d–k), transcellular pathways (l–o), transport pathways (p–q), and circulatory systems (r–t). B) Schematic illustration of the fabrication and oral delivery process of dexamethasone‐loaded hydroxyethyl starch‐curcumin nanoparticles with α‐amylase responsive ability (Reproduced with permission.^[^ ^171^ ^]^ Copyright 2022, Elsevier). C) Schematic illustration of the preparation and inner destiny of hybrid membrane‐coated nanoparticles with prolonged circulation time and preferential cancer targeting ability for chemophotodynamic synergistic therapy under irradiation (Reproduced with permission.^[^ ^175^ ^]^ Copyright 2023, American Chemical Society). D) Schematic illustration of the micelles based on a conjugate of *N*‐acetylcysteine‐modified hyaluronic acid and paclitaxel for efficient oral delivery of chemotherapy through mucosal bioadhesion (Reproduced with permission.^[^ ^185^ ^]^ Copyright 2018, Elsevier). E) Schematic illustration of the preventive and therapeutic effects of oral tea leaf‐derived natural nanotherapeutics with galactose receptor‐mediated endogenous pathway on the inflammatory bowel disease and colon cancer (Reproduced with permission.^[^ ^196^ ^]^ Copyright 2021, Elsevier). F) Schematic illustration of the paclitaxel‐loaded and glycocholic acid‐functionalized nanoliposomes with apical sodium‐dependent bile acid transporter (ASBT)‐mediated endogenous pathway and circulating ability (Reproduced with permission.^[^ ^208^ ^]^ Copyright 2022, Springer Nature).

Upon ingestion of bioactive food ingredients and their delivery carriers, the delivery systems are subjected to a multitude of enzymatic reactions. These reactions may influence the biopotency of the ingested substances. An understanding of these enzymatic reactions can be employed to develop bioactive delivery systems with enhanced resistance, controlled release, or tissue‐targeting capabilities (Table 1). The enzymatic activities and specificities of the enzymatic reactions must be of particular concern in the selection of this strategy. In certain instances, delivery vehicles are employed to encapsulate and protect bioactive enzymes (**Figure** **5**A,a). For instance, lipase and laccase have been encapsulated within antacid‐loaded alginate beads to protect them from deactivation within the gastric environment.^[^ ^163^, ^164^, ^165^ ^]^ In other cases, enzymes can be incorporated into delivery carriers to endow them with unique functions (Figure 5A,b). For instance, nanocarriers containing enzymes (hyaluronidase or collagenase) have been demonstrated to enhance the penetration of encapsulated substances into solid tumors, thereby improving treatment efficiency. Delivery vehicles decorated with other enzymes (papain or bromelain) have been shown to more readily cross the mucus barrier by cleaving mucin.^[^ ^166^, ^167^, ^168^ ^]^ Furthermore, delivery vehicles may be designed to resist enzyme hydrolysis or to release their cargoes in response to specific enzymatic triggers (Figure 5A,c). For instance, vehicles constructed from dietary fibers are resistant to enzymatic digestion within the upper GIT, whereas those constructed from proteins, starches, or lipids may be digested by proteases, amylases, or lipases in the mouth, stomach, or small intestine.^[^ ^169^, ^170^ ^]^ For example, Xu et al. (2022) fabricated self‐assembled polymeric micelles consisting of hydroxyethyl starch and curcumin, which could be triggered by α‐amylase to release the loaded dexamethasone (Figure 5B).^[^ ^171^ ^]^

### 5.4 Circulation‐Related Strategies

The circulatory systems, comprising the cardiovascular and lymphatic systems, play a pivotal role in transporting and distributing nutrients and bioactive agents throughout the body. Additionally, it is responsible for removing undesirable foreign matter from the body. Normally, ingested components are digested, absorbed, and then may be eliminated by the circulatory systems in a relatively short period (half‐life).^[^ ^172^ ^]^ A multitude of delivery systems is recognized as “foreign matter”, consequently attracting the attention of serum proteins in the bloodstream. The serum proteins then facilitate the ingestion of the delivery systems by macrophages, which are regulated by the reticuloendothelial system (RES) of the liver, spleen, and other organs.^[^ ^172^ ^]^ This phenomenon can potentially be exploited to design short‐circulating delivery systems with limited circulation time in the body, which may be advantageous for certain special applications (Figure 5A,t).^[^ ^173^, ^174^ ^]^ However, in most cases, long‐circulating delivery systems are required to extend the in vivo retention time of bioactive agents. It is therefore essential that these delivery systems are designed to resist blood clearance. For instance, these delivery systems may have biomimetic cell membrane structures or hydrophilic polymer‐modified surfaces. These structures can prevent recognition by proteins and macrophages in the bloodstream (Figure 5A,r,s; Figure 5C).^[^ ^172^, ^175^, ^176^ ^]^ For example, micelles made of super‐hydrophilic polymethylsulfobetaine conjugated with zein have been shown to have prolonged circulation time, which can increase the half‐life of curcumin by 22‐fold.^[^ ^176^ ^]^ This strategy requires care to avoid excessively long circulation time, which suggests that the delivery system may lack specific targeting ability.

### 5.5 Mucus‐Targeted Strategies

Mucosa are membranous structures composed of epithelial and connective tissues (such as lamina propria and muscularis mucosae), which are found in various organs, including the eyes, nose, mouth, respiratory tract, gastrointestinal tract, and reproductive tract.^[^ ^107^ ^]^ The outer mucosa is covered by a mucus layer secreted by goblet cells and specific glands under the regulation of mucin genes, which can reduce the risk of pathogen infection in the underlying epithelium and is the first line of immune defense.^[^ ^177^ ^]^ For oral delivery systems, the GIT mucus layer influences the absorption and bioavailability of encapsulated bioactive substances. The GIT mucus layer is the thickest in the stomach and colon and the thinnest in the small intestine. It can be divided into an inner layer with high viscosity and an outer layer with low viscosity.^[^ ^178^ ^]^ Mucus is a viscoelastic hydrogel with a highly crosslinked network structure, containing strongly negatively charged glycosylated fragments and numerous hydrophobic domains. The mucus layer provides lubrication and protection for the GIT, while also limiting the transport of large, cationic, and hydrophobic substances.^[^ ^177^, ^179^ ^]^ To enhance the cell penetration and uptake of these substances, two strategies are commonly adopted: 1) utilizing mucus layer features to design mucoadhesive carriers, or 2) overcoming mucus layer obstructions to develop mucus‐penetrating carriers (Table 1).

#### 5.5.1 Mucoadhesive Strategies

Mucosal adhesion is the process of establishing a connection between adherent polymers and mucus, as well as maintaining the adhesion state. Mucosal adhesion involves two distinct stages: contact and consolidation. A number of physicochemical mechanisms have been proposed to describe the mucosal adhesion, which has been reviewed in detail elsewhere.^[^ ^118^, ^180^ ^]^ Based on the properties of the polymer materials (size, shape, surface charge, hydrophilicity) and the characteristics of the mucus layer (composition, pH, viscosity, and thickness), mucoadhesive strategies can be designed based on physical interactions (such as entanglement, electrostatic interactions, hydrophobic interactions, and hydrogen bonding) or covalent interactions (such as disulfide bonding), or their combinations (Figure 5A,d,e).^[^ ^181^ ^]^

Natural polymers, such as chitosan, alginate, carrageenan, hyaluronic acid, dextran, cyclodextrin, gliadin, and collagen, as well as synthetic polymers such as polyacrylic acid and PEG, have been extensively employed in the food and pharmaceutical industries. Many of these polymers have been approved by the Food and Drug Administration (FDA).^[^ ^118^ ^]^ These polymers possess functional groups (such as amino, carboxyl, sulfate, and hydroxyl groups) that can physically crosslink with mucin through electrostatic and hydrogen bonding interactions, thereby prolonging the gastrointestinal retention time of oral delivery systems.^[^ ^177^, ^182^ ^]^ For instance, the electrostatic interactions between chitosan and mucus, as well as the hydrogen bonding interactions between dextran sulfate and mucin, have been used to prepare mucoadhesive nanoparticles, which could enhance the cellular uptake of coenzyme Q10.^[^ ^183^ ^]^ Furthermore, some polymers possess sulfhydryl groups that are capable of forming disulfide bonds with cysteine residues of mucin, thereby establishing a covalent attachment to the mucosal surfaces.^[^ ^184^ ^]^ For instance, researchers have constructed mucoadhesive micelles loaded with a hydrophobic drug (paclitaxel) by conjugating *N*‐acetylcysteine (NAC) to hyaluronic acid, which were covalently bound to mucin through disulfide bonds, thereby enhancing the bioavailability of the paclitaxel (Figure 5D).^[^ ^185^ ^]^ However, it should be noted that the mucus layer is regularly renewed, which may lead to the detachment and removal of some adherent delivery systems. Consequently, it is essential to avoid excessive adhesion when designing mucoadhesive delivery systems.^[^ ^179^, ^182^ ^]^

#### 5.5.2 Mucus‐Penetrating Strategies

Mucus penetration describes the process that oral delivery systems traverse the mucus layer and gain access to the epithelial layer of the gastrointestinal mucosa. Non‐destructive mucus‐penetrating strategies involve designing delivery systems with specific characteristics: an appropriate size (100–500 nm), shape (spherical or rod‐like), and surface charge (neutral or negative). Typically, these systems should be highly hydrophilic, so that they do not strongly bind to the mucus layer. This can be achieved using materials such as PEG, Pluronic F‐127 (PF‐127), polydopamine (PDA), polyvinyl alcohol (PVA), and polyacrylamide (PAM) (Figure 5A,f,g).^[^ ^177^, ^178^, ^186^ ^]^ As an example, the mucus penetration properties and anti‐inflammatory activity of resveratrol were enhanced by coating resveratrol‐loaded silk fibroin nanoparticles with hydrophilic non‐ionic long‐chain polymers (PF‐127).^[^ ^187^ ^]^ It was found that the administration of PF‐127‐coated nanoparticles could significantly inhibit the secretion of pro‐inflammatory tumor necrosis factor‐α (TNF‐α) and the generation of reactive oxygen species (ROS). Nevertheless, the modification of delivery systems using polymers with excessive molecular weights or packing densities may result in a reduction in mucus penetration ability.^[^ ^182^ ^]^

Destructive mucus‐penetrating strategies are frequently designed to disrupt the mucus layer. These strategies enhance the capacity of delivery systems to traverse the mucus layer, but they also increase the risk of pathogenic infection.^[^ ^118^ ^]^ Consequently, these strategies are seldom implemented in clinical practice. Due to the complexity of the mucus composition and structure, various strategies have been proposed to disrupt the mucus layer (Figure 5A,h–k): 1) enzymatic degradation of mucus components, e.g., using protease, neuraminidase, and deoxyribonuclease; 2) disturbance of mucus electrolytes, e.g., using chelating agents to bind Fe^3+^, Fe^2+^, and Ca^2+^, or hypertonic saline solutions to reduce mucin viscosity and disperse mucin bundles; 3) breakdown of mucus network crosslinking, e.g., using sulfides such as NAC and dithiothreitol (DTT) to break intermolecular and intramolecular disulfide bonds; 4) hindrance of mucus layer formation and stability, e.g., using *S*‐carboxymethlycysteine to inhibit the sialyltransferase activity of goblet cells, or using phospholipids to disrupt the junction between the mucus layer and the epithelial layer, thereby removing the mucus.^[^ ^168^, ^181^, ^188^ ^]^ Shahzadi et al. (2018) created self‐emulsifying oil‐in‐water (O/W) nanoemulsions with trypsin anchored to the surface, which could increase the mucus permeability of the nanoemulsions by disrupting the mucus layer.^[^ ^189^ ^]^

### 5.6 Cell‐Related Strategies

The most crucial prerequisite for oral delivery systems to be fully absorbed and utilized by the human body is to cross the epithelial or endothelial cell layer of various tissues and organs. Cell layers are highly selective and permeable structures that not only provide protection for the underlying tissues but also act as obstacles to the absorption and transport of oral delivery systems from the intestine into the bloodstream and then into the targeted tissues. Furthermore, the diversity of cell species and the complexity of cellular structures contribute to challenges in overcoming these natural barriers (Table 1).

#### 5.6.1 Cell‐Targeted Strategies

##### Transcellular Pathways‐Related Strategies

The selective permeability of epithelium layers depends on the compact membrane structures of individual cells, as well as the tight junctions between adjacent cells. Therefore, oral delivery carriers containing nutraceuticals or pharmaceuticals that are digested in the GIT may cross the intestinal epithelial cell layer and the endothelial cells of other tissues through two pathways: the transmembrane pathway and the paracellular pathway.^[^ ^190^ ^]^ These pathways can be further subdivided into active, passive (simple diffusion, assisted diffusion), and endocytosis/exocytosis transport according to the mechanisms involved.^[^ ^191^ ^]^

The phospholipid bilayers of cell membranes contain cholesterol, glycoproteins, and glycolipids, which modulate their fluidity and recognition capabilities.^[^ ^2^ ^]^ The bilayer membrane modulates the cell uptake of small molecules (such as glucose, vitamins, minerals, amino acids, and oligopeptides) through active or passive transport, and facilitates the transmembrane transport of macromolecular nutrients and colloidal particles via cell phagocytosis and intracellular vesicular transport.^[^ ^192^ ^]^ As with nutrients, the size, shape, charge, polarity, and surface chemistry of oral delivery systems determines the mechanism and efficiency of transmembrane transport.^[^ ^193^ ^]^ In general, delivery systems with larger dimensions either utilize cell membrane features to non‐destructively cross normal cell membranes or pass through damaged cell membranes destructively by additional physical or chemical means (Figure 5A,l–n). The non‐destructive strategies may be broadly categorized into three main groups: 1) EPR effect mediated nonspecific endocytosis, which can be utilized by modulating the surface properties of the delivery systems;^[^ ^194^ ^]^ 2) ligand‐receptor complex mediated specific endocytosis, which can be employed by chemically modifying the surfaces of the delivery systems (Figure 5E);^[^ ^195^, ^196^ ^]^ 3) cell membrane fluidity and high affinity mediated membrane fusion, which can be utilized by using natural or biomimetic cell membranes as the coatings of the delivery systems (also known as the cell membrane concealment strategy).^[^ ^197^ ^]^ As an example, the specific binding of hyaluronic acid to the “cluster of differentiation 44” (CD44) receptor on the surface of macrophages in colitis tissue has been successfully used to achieve cellular targeting and uptake of fabricated nanoparticles.^[^ ^195^ ^]^ Some of the most common strategies for the destructive permeation of cell membranes include: 1) irreversible perforation of cell membranes through the application of electric fields, magnetic fields, or microinjection, which are typically expensive and difficult to use;^[^ ^198^ ^]^ 2) reversible formation of transient pores or temporary channels on cell membranes through the construction of delivery systems with specific modifications, such as anionic oxidized carbon nanoparticles or phosphorothioate‐modified biomimetic DNA nanochannels;^[^ ^199^, ^200^ ^]^ 3) reversible or irreversible alteration of cell membranes through the use of absorption accelerators or osmotic enhancers.^[^ ^193^ ^]^ Typically, the exocytosis of delivery systems is less efficient than their endocytosis, because this process involves the fusion of lysosomes, multivesicular bodies, late endosomes, or caveolae with the plasma membrane.^[^ ^192^ ^]^

Intercellular tight junctions (TJs) are composed of integral transmembrane proteins (claudins, occludin, junctional adhesion molecule, tricelluin) and intracellular cytosolic scaffold proteins (zonula occludens), which are situated at the apical ends of the cell lateral membrane.^[^ ^201^ ^]^ The integrity and dynamic regulation of TJs are essential for maintaining the permeability and balance of ions, water, and small nutrients across the intercellular space. The functionality of TJs is influenced by numerous cytokines, growth factors, and nutrients.^[^ ^190^, ^202^ ^]^ TJs regulate the selective permeability of intercellular spaces, which not only maintains the permeability and equilibrium of ions, water, and small nutrients, but also prevents the diffusion of large nutrients and delivery systems across the intercellular space. Oral delivery systems may employ either irreversible or reversible strategies to open or loosen the TJs, thus facilitating their transport across cell layers via a paracellular pathway (Figure 5A,o). TJs can be damaged by the application of disruptive physical forces, toxic chemicals, or penetration enhancers (such as chelators, cationic polymers, nanotubes, toxins, phytochemicals, and phosphatase inhibitors).^[^ ^203^, ^204^ ^]^ For example, cationic chitosan and zonula occludins toxin (ZOT)‐derived tight junction opening peptides have been used to fabricate functionalized insulin‐loaded nanocarriers, which were observed to cross the small intestinal epithelial cells via the paracellular pathway.^[^ ^205^ ^]^ In another study, α‐lactalbumin nanotubes were shown to transiently and reversibly open TJs and promote blood circulation.^[^ ^206^ ^]^

It is important to note that the damaged cell barriers permit the penetration of harmful substances from the intestinal lumen or circulatory system into the mucosal tissues or targeted organs, which can promote inflammation and tissue damage.^[^ ^192^ ^]^ Consequently, delivery systems that rely on non‐destructive strategies to increase penetration are more commonly used.

##### Transport Pathways‐Related Strategies

As illustrated in Figure 2B, cells within the intestinal epithelium and basement membrane can be classified into three main categories: secretory cells (goblet cells, Paneth cells, enteroendocrine cells), absorptive cells (enterocytes, M cells, tuft cells), and intraepithelial lymphocytes (macrophages, dendritic cells, T cells, B cells). The intestinal mucosal immune system (IMIS) is composed of scattered immune cells, the intestinal‐associated lymphoid tissues, and the mesenteric lymph nodes distributed beneath the intestinal epithelium layer.^[^ ^207^ ^]^ Therefore, when the administrated delivery system encounters cellular barriers, it will either be regarded as a “nutrient” and then selectively absorbed and transported via enterocytes into the systemic circulation through transcellular or paracellular pathways, or it will be recognized as a “foreign body”. It may then be presented or ingested by intestinal immune cells within the IMIS. Subsequently, it enters the blood circulation through the lymphatic circulation or reaches the target organs (Figure 5A,p,q). For instance, paclitaxel‐loaded modified liposomes were internalized by enterocytes via endocytosis and subsequently released into the systemic circulation after lysosomal escape (Figure 5F).^[^ ^208^ ^]^ In another study, self‐assembled nanoparticles consisting of doxorubicin, disulfide‐containing linkers, and β‐glucans were shown to specifically target intestinal M cells, be phagocytosed by local macrophages, and finally cross the BBB of glioma‐bearing mice via the lymphatic circulatory system.^[^ ^209^ ^]^ Currently, the lymphatic system is one of the most widely used targeting sites for nutraceutical and pharmaceutical delivery to improve the bioavailability of bioactive components by avoiding the first‐pass elimination effect.^[^ ^210^ ^]^ Nevertheless, the lymphatic system may also reduce the specific targeting capacity of certain delivery systems because it is widespread in multiple tissues and organs, which precludes the precise targeting of a specific tissue or organ using this strategy.

#### 5.6.2 Organelle‐Targeted Strategies

**Figure 6:**
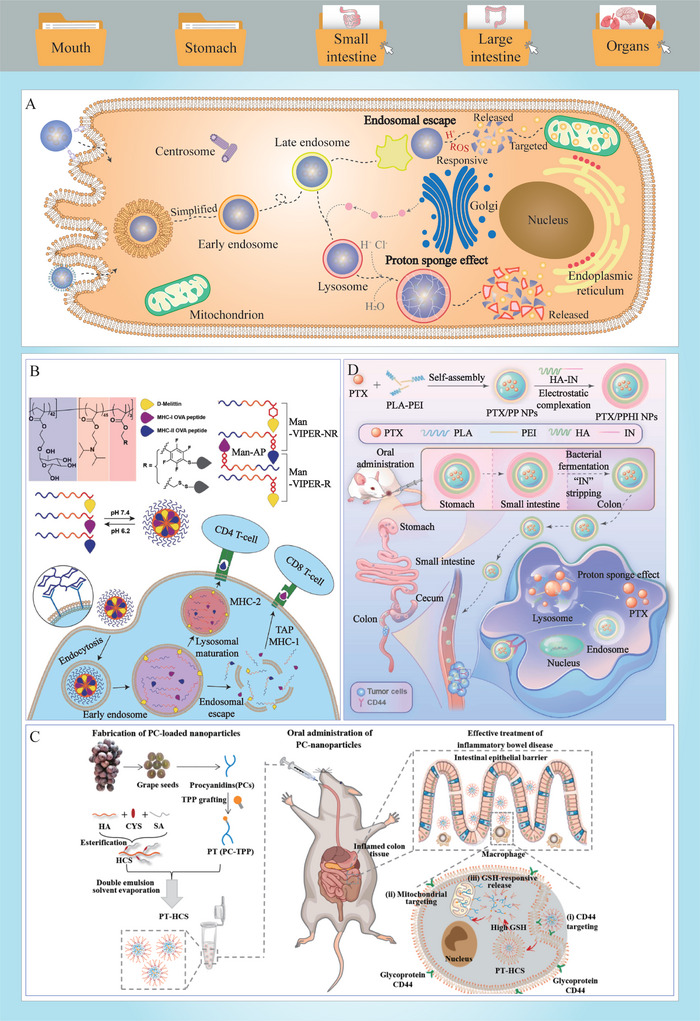
Main strategies for overcoming the organelle barriers in intestines and targeted organs. A) Two responsive release modes of the delivery systems taken by cells. B) Schematic illustration of the self‐assembling, pH‐sensitive, mannosylated polymeric micelles with different organelle responsiveness in dendritic cells (Reproduced with permission.^[^ ^213^ ^]^ Copyright 2023, Elsevier). C) Schematic illustration of the fabrication and treatment mechanism of procyanidin‐loaded composite nanoparticles with glutathione responsiveness, as well as CD44 and mitochondria targeting abilities (Reproduced with permission.^[^ ^195^ ^]^ Copyright 2022, Elsevier). D) Schematic illustration of the preparation and intracellular therapeutic mechanism of paclitaxel‐loaded inulin‐hyaluronic acid‐modified double‐layer polymeric nanoparticles with the ability to trigger proton sponge effect (Reproduced with permission.^[^ ^9^ ^]^ Copyright 2022, Elsevier).

Organelles are micro‐organs or subcellular structures with specific morphologies and functions that are scattered throughout the cytoplasmic matrix. In human cells, organelles include nuclear endosomes, mitochondria, lysosomes, endoplasmic reticulum, Golgi apparatus, centrosome, and the nucleus (**Figure** **6**A). Organelles maintain cellular homeostasis and basic life activities, and impact the fate of oral delivery systems after cellular uptake.^[^ ^211^ ^]^ Recently, organelle and subcellular targeting strategies have become the focus of research aimed at improving the efficacy of delivery systems.^[^ ^81^ ^]^ Typically, delivery systems taken up by cells are encapsulated in vesicles formed by the invaginated plasma membrane to produce endosomes. Some of the encapsulated delivery systems may then be released from the endosomes into the surrounding cytoplasmic matrix (“endosomal escape”), which may be triggered by specific cytoplasmic conditions or following cytoplasmic transport to targeted organelles (Figure 6B).^[^ ^212^, ^213^ ^]^ For example, Tie et al. (2022) constructed a composite nanoparticle that could respond to the high glutathione environment in the macrophages of colon‐inflamed mice and target to mitochondria with negative membrane potentials (Figure 6C).^[^ ^195^ ^]^ However, most early endosomes mature into late endosomes and eventually into lysosomes. These processes can lead to the release of delivery systems either by the proton sponge effect or by their expulsion after enzymatic degradation of the lysosomes (“lysosomal escape”).^[^ ^192^, ^211^, ^213^ ^]^ Hou et al. (2022) prepared paclitaxel‐loaded composite nanoparticles that could promote lysosome swelling and rupture, thereby releasing the anticancer drug into the tumor cells (Figure 6D).^[^ ^9^ ^]^ Furthermore, it has been demonstrated that particles or liposomes can be developed for targeting the nucleus or endoplasmic reticulum. These particles or liposomes utilize nuclear pore traversing, specific binding, or membrane‐fusing approaches to achieve targeting capacity.^[^ ^211^ ^]^ Therefore, accurately designing organelle‐targeted delivery systems is of great significance for achieving precision nutrition or drug therapy. This strategy requires extra attention to the physicochemical properties of different organelles and the prevention of off‐target effects.

#### 5.6.3 Off‐Target Effects

The off‐target effect is defined as the release of the delivered nutrients or drugs at a site other than that for which the strategy was designed, resulting in certain unintended effects. In the design of oral delivery systems, it is important to ensure that any undesirable or unintended off‐target effects are minimized. Ideally, a delivery system should only be absorbed by the target cells or organelles. In the event that a delivery system is incorporated by non‐target cells, it is of great importance to ensure that it does not cause damage to these cells. Since it is possible that undesirable interactions between delivery systems and organelles, as well as unintended core leakage, could cause local inflammation and cell apoptosis. Once delivery systems are absorbed by target cells, it is essential to identify suitable conditions that will facilitate the release of the bioactives to the desired targets. This can be achieved by utilizing specific intracellular microenvironments to induce delivery system lysis, or by employing specific interactions between the delivery systems and target organelles to facilitate carrier destruction or bioactive release.

### 5.7 Gut Flora‐Targeted Strategies

The gut microbiota is a critical ecosystem within the digestive tract that interacts with other tissues and organs.^[^ ^214^ ^]^ These microorganisms are primarily located in the large intestine, where they form three distinct layers: 1) the deep membrane flora layer dominated by commensal bacteria; 2) the middle flora layer dominated by anaerobic bacteria; and 3) the surface luminal flora layer dominated by aerobic and facultative anaerobic bacteria.^[^ ^215^ ^]^ The maintenance of intestinal homeostasis is typically the result of interactions between the different bacteria (Table 1). A disturbance of the intestinal flora can result in an imbalance of the intestinal environment, which may in turn lead to the development of various intestinal diseases, including diarrhea, colitis, and rectal cancer.^[^ ^216^ ^]^ In addition, intestinal microorganisms may indirectly regulate other tissues or organs in the human body through the IMIS, the enteric nervous system, and the gut‐brain or gut‐liver axes supported by the intestinal circulatory system.^[^ ^217^ ^]^

**Figure 7:**
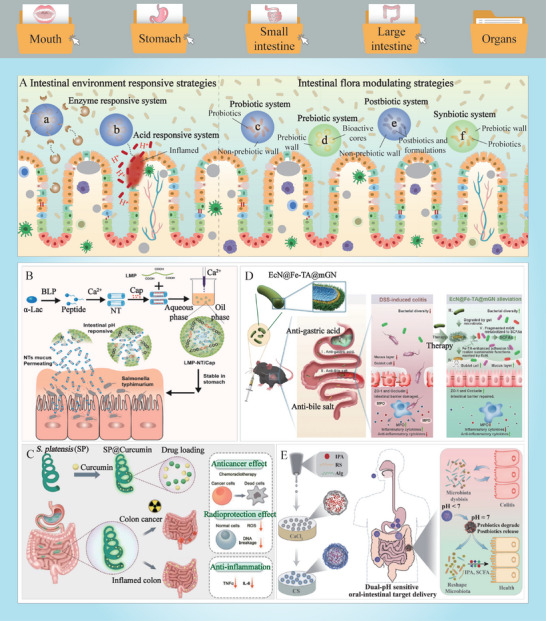
Main strategies for overcoming the microbiological barriers in oral cavity and GIT. A) Delivery systems that can respond to intestinal environment (a, b) and modulate intestinal flora (c–f). B) Schematic illustration of the preparation and therapeutic effect of capsaicin‐loaded α‐lactalbumin nanotubes @ low‐methoxy pectin microgels composite delivery systems with intestinal pH‐responsive ability on enteritis caused by *Salmonella typhimurium* infection (Reproduced with permission.^[^ ^133^ ^]^ Copyright 2022, Elsevier). C) Schematic illustration of the preparation, chemoradiotherapeutic, and anti‐inflammatory effects of curcumin‐loaded *Spirulina platensis* with intestinal enzyme‐responsive ability and prebiotic function on colon cancer and intestinal inflammation (Reproduced with permission.^[^ ^218^ ^]^ Copyright 2021, American Association for the Advancement of Science). D) Schematic illustration of the fabrication of *Escherichia coli* Nissle 1917 @ Fe^3+^‐tannic acid @ carboxymethylated β‐glucan synbiotic system and its alleviation mechanism for dextran sulfate sodium (DSS)‐induced colitis in mice (Reproduced with permission.^[^ ^223^ ^]^ Copyright 2023, American Chemical Society). E) Schematic illustration of the microcapsules that consist of chitosan‐coated prebiotics (resistant starch, alginate) and postbiotic (indole‐3‐propionic acid) for preventing and treating colitis via responding to intestinal environment and modulating gut microbiota (Reproduced with permission.^[^ ^224^ ^]^ Copyright 2022, Wiley‐VCH GmbH).

The intestinal environment is characterized by a high degree of biological and chemical complexity, which is largely attributable to the diverse microorganisms and their metabolites. The specific conditions, including pathological microenvironments and special metabolites, within the intestines have been employed to construct delivery systems with intestinal responsive or targeting capabilities (**Figure** **7**A,a,b). For example, regions with active gut microorganisms or inflammation frequently exhibit a lower pH environment due to the production of acidic metabolites.^[^ ^133^ ^]^ Consequently, pH‐responsive delivery systems can be employed to deliver bioactives to these regions (Figure 7B). Moreover, interactions between delivery systems with inulin coating and gut microbes that produce inulinase have been used to construct composite nanoparticles with colon‐targeting ability.^[^ ^9^ ^]^ Other researchers have utilized the enzymes produced by intestinal flora to degrade *Spirulina platensis* and release curcumin for the treatment of colitis and colon cancer (Figure 7C).^[^ ^218^ ^]^ It is of paramount importance that the aforementioned strategies should pay attention to the composition and specificity of intestinal environments.

It is possible to establish beneficial intestinal flora through a number of different delivery strategies: 1) the administration of probiotics (living bacteria) is an effective method of increasing beneficial gut bacteria and preventing intestinal diseases by resisting pathogen adhesion and colonization (Figure 7A,c); 2) the administration of prebiotics, such as cellulose, resistant starch, oligofructose, and inulin, which are food components that can stimulate the growth of beneficial bacteria in intestines (Figure 7A,d); 3) the administration of postbiotics (non‐living bacteria) is also an effective approach for exerting beneficial effects on intestinal health due to the constituents they contain (Figure 7A,e); 4) the administration of synbiotic systems that contain prebiotics, probiotics, and/or postbiotics can also be used to modulate the gut flora (Figure 7A,f).^[^ ^219^, ^220^, ^221^, ^222^ ^]^ For example, Xie et al. (2023) developed a synbiotic delivery system comprising modified β‐glucan as prebiotics and *Escherichia coli* Nissle 1917 as probiotics (Figure 7D).^[^ ^223^ ^]^ Furthermore, Yang et al. (2022) developed a prebiotic‐postbiotic synergistic microcapsule for preventing and alleviating colitis (Figure 7E).^[^ ^224^ ^]^ It is important to consider the probiotic activity and prebiotic potential of certain natural substances when employing these strategies.

### 5.8 Organ‐Targeted Strategies

The human body and its normal life activities are supported by a variety of complex organs, including receptive organs (eyes, nose, ears, mouth, skin), visceral organs (brain, heart, liver, spleen, lung, kidney, stomach, intestine, reproductive organs, etc.), and muscular organs.^[^ ^225^ ^]^ To improve the bioavailability of dietary bioactives, it is necessary to develop nutraceutical‐loaded delivery systems with organ‐targeting capabilities. Consequently, a variety of organ‐targeted delivery strategies have been proposed for the oral, transcutaneous, nasal, and intravenous administration of bioactives. These strategies can be designed according to the following two pathways:

1. *Direct Pathways*: delivery to the targeted organs by infiltration, smearing, or oral administration.
2. *Indirect Pathways*: entry into the circulatory systems via destructive routes (e.g., intravenous injection) or nondestructive routes (e.g., receptive organs‐mediated), and then arrive at the targeted organs.

**Figure 8:**
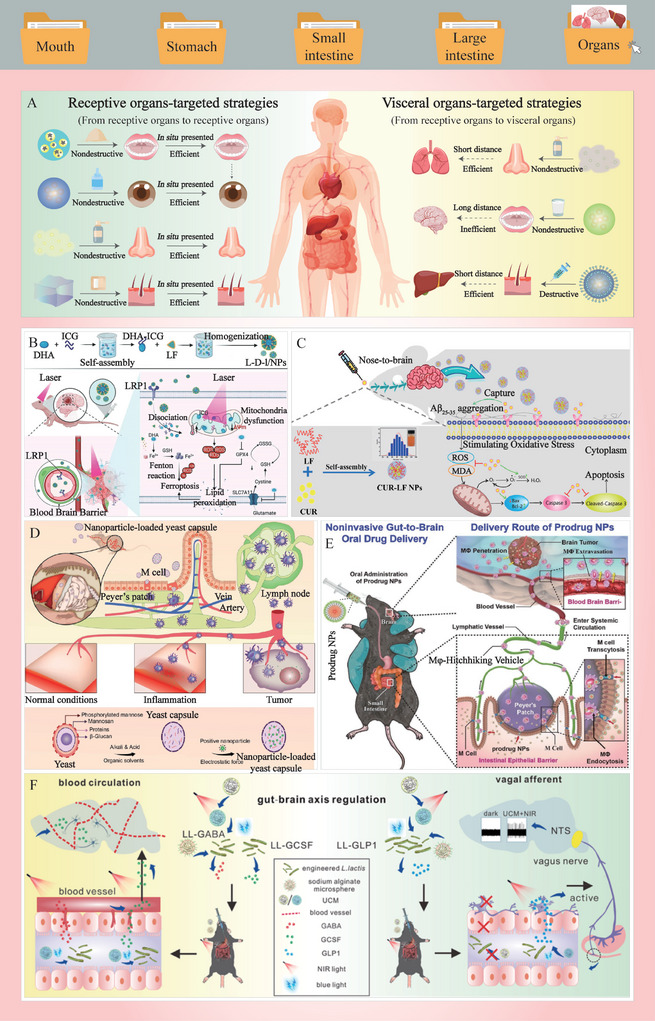
Main strategies for achieving organ‐targeted delivery. A) Graphical illustration of approaches for receptive organs and visceral organs targeting. B) Schematic illustration of the preparation of lactoferrin‐dihydroartemisinin‐indocyanine green biomimetic nanoplatforms and the underlying mechanism for treating glioblastoma multiforme (Reproduced with permission.^[^ ^229^ ^]^ Copyright 2023, Wiley‐VCH GmbH). C) Schematic illustration of the preparation of curcumin‐lactoferrin nanoparticles and the mechanism for achieving neuroprotection via intranasal administration (Reproduced with permission.^[^ ^232^ ^]^ Copyright 2023, Frontiers Media S.A.). D) Schematic diagram of the translocation and preparation of yeast capsule‐mediated nanoparticles for oral targeting the inflammation‐associated disease sites that distant from GIT through the immune system (Reproduced with permission.^[^ ^236^ ^]^ Copyright 2017, American Chemical Society). E) A noninvasive gut‐to‐brain oral delivery system constructed by the self‐assembling conjugates consist of hydrophobic agents, disulfide‐containing linkers (2,2′‐dithiolethanol), and β‐glucans, as well as its mechanism based on the immune system (Reproduced with permission.^[^ ^209^ ^]^ Copyright 2021, Wiley‐VCH GmbH). F) Schematic illustration of gut‐brain axis regulation via an up‐conversion optogenetic micro‐nano system including three blue light‐responsive probiotics (Reproduced with permission.^[^ ^237^ ^]^ Copyright 2022, American Chemical Society).

For easily accessing to the receptive organs, such as the eyes, nose, mouth, and skin, delivery systems can be administered nondestructively by in situ presentation (form receptive organs to receptive organs). These delivery systems mainly include suspensions, foams, sprays, and gels, and are usually formulated as eye drops, aerosols, or wound dressings for targeting the receptive organs (**Figure** **8**A).^[^ ^226^, ^227^, ^228^ ^]^ Therefore, receptive organs‐targeted strategies generally represent high efficiency, convenience, and acceptance. However, these strategies are more commonly used to treat superficial diseases rather than internal organic lesions. In contrast, visceral organs‐targeted strategies have lower efficacy due to the difficulty of reaching those organs that located inside the human body. The majority of visceral organs‐targeted delivery systems use intravenous injection to achieve low gastrointestinal toxicity, high bioavailability, and dose‐controlled property. However, as shown in Figure 8B, intravenous injection delivery may lead to physiological damage and psychological resistance of the treated subjects.^[^ ^229^, ^230^ ^]^ Consequently, nondestructive visceral organs‐targeted delivery systems are needing to be developed, which can be categorized into the relatively efficient “short‐distance receptive organs‐visceral organs” delivery (e.g., lung‐targeting via the nasal cavity‐respiratory tract, brain‐targeting via the nasal cavity‐mucosa) and the relatively inefficient “long‐distance receptive organs‐visceral organs” delivery (e.g., liver‐or brain‐targeting via the mouth‐GIT‐circulatory systems) (Figure 8A,C,E).^[^ ^209^, ^231^, ^232^, ^233^ ^]^ Methods for oral delivering and targeting bioactives to different organs (especially the brain) are briefly summarized in Table 1 and partially illustrated in Figure 8D–F.^[^ ^234^, ^235^, ^236^, ^237^, ^238^, ^239^ ^]^ For example, oral brain‐targeting delivery can be achieved mainly through the strategies that taking advantages of the brain‐targeting core or wall materials, the immune system, the gut‐brain axis, the external physical stimuli, and the pathological microenvironments of brain. However, the specific mechanisms and efficacy of these strategies need to be further explored and investigated.

## 6 Challenges and Future Perspectives

### 6.1 Challenges to the Development of Biopotency Enhanced Oral Delivery Systems

#### 6.1.1 The Properties of Oral Delivery Systems Need to Be Improved

There are still several hurdles that must be overcome when constructing effective oral delivery systems with required properties. These delivery systems are typically composed of diverse materials, which are held together through covalent and/or noncovalent interactions. Based on these interactions, if the delivery systems are too stable, they may prevent the release of bioactive substances at target sites. In contrast, if the delivery systems are too unstable, they may release the encapsulated bioactive substances before reach their intended destination. Both of these situations have the potential to negatively impact the efficacy of encapsulated bioactive substances. Moreover, many delivery systems may be ineffective in overcoming the various barriers inside and outside the human body. Following ingestion, these delivery systems may be degraded by enzymes or phagocytized by macrophages.^[^ ^240^ ^]^ Consequently, the concentration of bioactive substances that ultimately reach the bloodstream or the target organs/tissues may be insufficient to achieve the desired physiological effects.^[^ ^241^ ^]^ Furthermore, certain materials utilized in the preparation of delivery systems may exhibit toxicity. For instance, Hou et al. (2022) used polylactic acid (PLA) and polyethyleneimine (PEI) to fabricate nanoparticles and encapsulate a hydrophobic chemotherapeutic drug (paclitaxel).^[^ ^9^ ^]^ The PLA was found to be non‐toxic, whereas the PEI exhibited some toxicity. Wang et al. (2023) prepared polymer‐based particles composed of hyaluronic acid and bilirubin for encapsulating and delivering probiotics (*Bifidobacterium longum*).^[^ ^242^ ^]^ However, bilirubin may cause damage to the brain and nervous system. Several researchers have demonstrated the low cytotoxicity of some delivery systems using cell and/or animal experiments. However, the potential toxicity of these delivery systems to humans with long‐term usage has not been investigated in most studies.

#### 6.1.2 The Performances of Delivery Systems Require Standardized Evaluation Methods

A multitude of different delivery systems are currently being developed to encapsulate, protect, and release a variety of bioactive substances with different therapeutic goals. However, the fate of delivery systems in the human body is highly complex and difficult to assess. In order to accurately evaluate the efficacy of these delivery systems for specific applications, it will be necessary to develop standardized protocols for their evaluation. This will enable the optimal delivery system to be identified for each bioactive substance and therapeutic application. Over the years, researchers have employed a variety of methods to evaluate the performance of delivery systems, including numerous in vitro and in vivo methods that measure their properties, interactions, stabilities, release/retention profiles, absorption mechanisms, bioavailability, transport behavior, and potential toxicity. Carbonell–Capella et al. (2014) provided a comprehensive review of the commonly used test methods.^[^ ^243^ ^]^ For example, the efficacy of delivery systems can be assessed using physicochemical analysis, simulated gastrointestinal models, cell models, animal feeding studies, and human feeding studies. In comparison to the in vivo methods, in vitro methods are typically faster, cheaper, simpler, more reproducible, and have fewer ethical limitations. However, they are less representative and less accurate. Consequently, an increasing number of researchers are integrating in vitro and in vivo methodologies to enhance their understanding of the oral delivery processes. For instance, Miao et al. (2021) validated the immune cell uptake, tumor homing capacity, and transport pathway of prodrugs using both an in vitro macrophage model and an in vivo brain tumor‐bearing mice model.^[^ ^209^ ^]^ In summary, there is still a pressing need to develop precise, reliable, and reproducible standardized evaluation models for comparing the results from different studies.

#### 6.1.3 The Practical Applications of Oral Delivery Systems Are Currently Limited

The transition of oral delivery systems from laboratory scale to practical utilization represents a pivotal step in the transformation of their potential health benefits from theory to practice in the food industry. However, several practical issues need to be addressed, including the impacts on food quality, affordability, robustness, labeling, consumer acceptance, sustainability, environmental friendliness, regulation, and safety. To date, the majority of oral delivery systems developed in laboratory settings have been proven unsuitable for industrial applications, as they are unable to fully meet the above criteria. For example, it is crucial to guarantee that the incorporation of delivery systems does not adversely affect the desired appearance, mouthfeel, aroma, and taste of functional food and beverage products. Luan et al. (2022) constructed “wooden rolls” that could deliver probiotics and bioactive substances. However, the relatively large size of these rolls may result in unpleasant mouthfeel, difficulty in swallowing, and psychological resistance from consumers.^[^ ^54^ ^]^ Furthermore, the production of delivery systems is often costly due to the need for purified bioactive substances, complex production methods, and expensive manufacturing equipments, which may render them unaffordable for many applications. Consequently, it is of great importance to ensure that the delivery systems developed in research laboratories are relatively inexpensive and can be produced on a large scale, which are often ignored in the initial research and development stages.

### 6.2 Future Perspectives of Health Promoting Oral Delivery Systems

The majority of oral delivery systems are based on single encapsulation technology, such as microemulsions, nanoemulsions, emulsions, nanovesicles, or microgels. Consequently, they exhibit limited functional attributes. In the future, it may be advantageous to integrate several encapsulation technologies into a delivery system to enhance its functional performances. For instance, nanoemulsions or nanoliposomes can be encapsulated within microgels to protect the bioactive‐loaded nanocarriers and to regulate the bioactive release profiles. Similarly, gelling agents can be added to the aqueous phase of oil‐in‐water emulsions to form emulsion gels, which exhibit novel functional attributes, such as semi‐solid textures and prolonged release profiles.^[^ ^244^ ^]^

At present, the majority of oral delivery systems are designed to contain single bioactive substance. However, in the future, it may be more desirable to incorporate a variety of different bioactive agents into a delivery system for personalized nutrition applications. For instance, vitamins, minerals, and phytochemicals can be incorporated into a delivery system to meet the specific nutritional requirements of individuals. The delivery of two or more bioactive ingredients with different physiological mechanisms may lead to the emergence of synergistic health benefits. For instance, Jiao et al. (2018) reported that the delivery of both V_C_ and folic acid by chitosan‐coated liposomes resulted in synergistic antioxidant effects.^[^ ^245^ ^]^ In principle, multi‐bioactive‐loaded delivery systems can be designed to release the co‐encapsulated bioactive agents either simultaneously or sequentially, depending on the requirements of specific applications.

A significant proportion of oral delivery systems are constructed from synthetic ingredients, especially synthetic surfactants and polymers. These materials may be associated with health concerns, such as the dysregulation of microbiome or gut inflammation. To address the potential toxicity concerns in the long‐term utilization of oral delivery systems, there has been a growing interest in the use of plant‐based ingredients for their construction. Furthermore, plant‐based ingredients offer advantages in terms of improved sustainability, reduced environmental impact, and label friendliness. Consequently, there is a clear need for further researches on the development of plant‐based oral delivery systems that are stable and effective.

As previously stated, there is an urgent need to develop uniform in vitro and in vivo testing protocols for the assessment and comparison of different oral delivery systems under identical conditions. It is recommended that standardized methods should be developed to provide information about the properties of the delivery systems, the behavior and potential mechanisms of these systems as they pass through the human GIT, and the evaluation of their biopotency. Finally, the successful construction and comprehensive evaluation of oral delivery systems typically require the integration of multiple research fields and techniques. In the future, it is possible that various oral delivery strategies will be combined to meet the differentiated nutritional needs of consumers.

## 7 Conclusion and Prospects

A wide variety of bioactive ingredients can be incorporated into functional foods and beverages to improve human health and well‐being, such as vitamins, minerals, polyphenols, prebiotics, and probiotics. However, many of these bioactive ingredients are unable to exert their beneficial health effects due to poor solubility, poor stability, or low bioavailability. These deficiencies can be overcome through the use of encapsulation technologies, which can enhance the dispersibility, stability, and bioavailability of bioactive ingredients. A plethora of encapsulation technologies are available for the creation of oral delivery systems, each with distinct characteristics. These characteristics include particle properties (e.g., composition, size, shape, charge, and responsiveness) and physicochemical attributes (e.g., clear/opaque, liquid/solid, stable/unstable), which allow for the selection of the most appropriate system for each specific application. However, there is still a relatively poor understanding of which system is the most suitable for each application. Moreover, there is a need to gain a deeper understanding of how each type of delivery system responds to the various barriers encountered along the human gastrointestinal tract and other sites. This review has demonstrated that there are multiple strategies for overcoming specific biopotency barriers. Consequently, it is important to design delivery systems that overcome the specific barriers associated with each specific application. Many challenges remain for the successful development and application of oral delivery systems in the food industry and other sectors. Ideally, the delivery systems should be assembled by safe, affordable, sustainable, and consumer‐friendly ingredients using manufacturing processes that are economically viable and scalable. Moreover, standardized methods are needed to test and compare the performance of different oral delivery systems. This will facilitate the identification of the most suitable delivery system for each specific application. Finally, it is necessary to conduct in vivo studies to evaluate the efficacy and safety of different oral delivery systems. These studies will provide evidence for delivery systems of the ability to improve human health and wellbeing, without causing any adverse effects.

## Conflict of Interest

The authors declare no conflict of interest.

## Author Contributions

L.L. performed conceptualization, methodology, software, and visualization, wrote the original draft, and reviewed and edited the final manuscript, D.J.M. performed conceptualization, methodology, reviewed and edited the final manuscript, X.L. performed supervision, acquired resources, and wrote, reviewed, and edited the final manuscript. F.L. performed conceptualization, methodology, supervision, funding and resources acquisition, and wrote, reviewed, and edited the final manuscript.

## Supporting information



Supporting Information

## References

[1] Q. Lu , R. Li , Y. Yang , Y. Zhang , Q. Zhao , J. Li , Food Chem. 2022, 368, 130610. 34419798 10.1016/j.foodchem.2021.130610

[2] Y. Pan , H. Y. Li , F. Shahidi , T. Luo , Z. Y. Deng , Trends Food Sci. Technol. 2022, 124, 38.

[3] W. Chen , H. Su , J Zhejiang Univ Sci B 2023, 24, 549. 37455133 10.1631/jzus.B2310001 PMC10350371

[4] T. Liu , Y. Zhao , N. Wu , S. Chen , M. Xu , H. Du , Y. Yao , Y. Tu , Crit. Rev. Food Sci. Nutr. 2022, 64, 617. 35930299 10.1080/10408398.2022.2107612

[5] D. J. McClements , F. Li , H. Xiao , Annu. Rev. Food Sci. Technol. 2015, 6, 299. 25705933 10.1146/annurev-food-032814-014043

[6] Y. Guo , D. Qiao , S. Zhao , B. Zhang , F. Xie , Carbohyd. Polym. 2021, 270, 118358. 10.1016/j.carbpol.2021.118358 34364603

[7] M.‐J. Teng , Y.‐S. Wei , T.‐G. Hu , Y. Zhang , K. Feng , M.‐H. Zong , H. Wu , J. Food Eng. 2020, 281, 109993.

[8] H. D. Silva , J. Poejo , A. C. Pinheiro , F. Donsì , A. T. Serra , C. M. M. Duarte , G. Ferrari , M. A. Cerqueira , A. A. Vicente , J Funct Foods 2018, 48, 605.

[9] Y. Hou , J. Jin , H. Duan , C. Liu , L. Chen , W. Huang , Z. Gao , M. Jin , Biomaterials 2022, 283, 121440. 35245731 10.1016/j.biomaterials.2022.121440

[10] V. Toragall , N. Jayapala , S. P. Muthukumar , B. Vallikanan , Food Chem. 2020, 330, 127195. 32585586 10.1016/j.foodchem.2020.127195

[11] C. Dima , E. Assadpour , S. Dima , S. M. Jafari , Compr Rev Food Sci Food Saf 2020, 19, 2862. 33337033 10.1111/1541-4337.12623

[12] C‐L. Jiang , X.‐Y. Li , W‐D. Shen , L.‐H Pan , Q.‐M. Li , J.‐P. Luo , X.‐Q. Zha , J Food Biochem 2022, 46, e14337. 35945814 10.1111/jfbc.14337

[13] L. Münster , M. Fojtu , Z. Capáková , M. Muchová , L. Musilová , T. S. Vaculovic , J. Balvan , I. Kuritka , M. Masařík , J. Vícha , Carbohyd. Polym. 2021, 257, 117562. 10.1016/j.carbpol.2020.117562 33541627

[14] A. Muxika , A. Etxabide , J. Uranga , P. Guerrero , K. de la Caba , Int. J. Biol. Macromol. 2017, 105, 1358. 28735006 10.1016/j.ijbiomac.2017.07.087

[15] H. Yildiz , N. Karatas , Process Biochemistry 2018, 72, 41.

[16] X.‐L. Xu , S. Li , R. Zhang , W.‐D. Le , Neural Regen Res 2022, 17, 1907. 35142666 10.4103/1673-5374.335142 PMC8848587

[17] J. Li , C. Cai , J. Li , J. Li , J. Li , T. Sun , L. Wang , H. Wu , G. Yu , Molecules 2018, 23, 2661. 30332830 10.3390/molecules23102661 PMC6222903

[18] F. Abasalizadeh , S. V. Moghaddam , E. Alizadeh , E. akbari , E. Kashani , S. M. B. Fazljou , M. Torbati , A. Akbarzadeh , J. Biol. Eng. 2020, 14, 8. 32190110 10.1186/s13036-020-0227-7 PMC7069202

[19] G. L. Huang , H. L. Huang , Drug Delivery 2018, 25, 766. 29536778 10.1080/10717544.2018.1450910 PMC6058522

[20] M. Luo , X. Zhang , J. Wu , J. Zhao , Carbohyd. Polym. 2021, 266, 118097. 10.1016/j.carbpol.2021.118097 34044964

[21] M. Akbarian , A. Khani , S. Eghbalpour , V. N. Uversky , Int. J. Mol. Sci. 2022, 23, 1445. 35163367 10.3390/ijms23031445 PMC8836030

[22] M. Duarte Villas Mishima , H. Stampini Duarte Martino , T. Silva Meneguelli , E. Tako , Crit. Rev. Food Sci. Nutr. 2023, 1. 10.1080/10408398.2023.2245469 37574588

[23] R. H. Pain , Nature 1979, 279, 824.

[24] T. Guan , Z. Zhang , X. Li , S. Cui , D. J. McClements , X. Wu , L. Chen , J. Long , A. Jiao , C. Qiu , Z. Jin , Foods 2022, 11, 1562. 35681312 10.3390/foods11111562 PMC9180007

[25] H. Chen , X. Tan , X. Han , L. Ma , H. Dai , Y. Fu , Y. Zhang , Biotechnol. Adv. 2022, 61, 108037. 36152892 10.1016/j.biotechadv.2022.108037

[26] T. B. Ng , R. C. F. Cheung , J. H. Wong , Y. S. Chan , X. Dan , W. Pan , H. Wang , S. Guan , K. Chan , X. Ye , F. Liu , L. Xia , W. Y. Chan , Appl. Microbiol. Biotechnol. 2016, 100, 6601. 27338574 10.1007/s00253-016-7671-9

[27] N. Auestad , D. K. Layman , Nutr Rev 2021, 79, 36. 34879145 10.1093/nutrit/nuab097 PMC8653944

[28] F. Casanova , L. G. L. Nascimento , N. F. N. Silva , A. F. de Carvalho , F. Gaucheron , Food Chem 2021, 359, 129820. 33962195 10.1016/j.foodchem.2021.129820

[29] Y.‐S. Wei , K. Feng , S.‐F. Li , T.‐G. Hu , R. J. Linhardt , M.‐H. Zong , H. Wu , Crit. Rev. Food Sci. Nutr. 2021, 62, 6341. 33749401 10.1080/10408398.2021.1900774

[30] F. G. Liu , S. H. Zhang , J. Y. Li , D. J. McClements , X. B. Liu , Trends Food Sci. Technol. 2018, 79, 67.

[31] Y. Zhang , T. Zhang , Y. Liang , L. Jiang , X. Sui , J. Agric. Food Chem. 2021, 69, 8929. 34161727 10.1021/acs.jafc.1c01369

[32] S. D. Angelo , M. L. Motti , R. Meccariello , Nutrients 2020, 12, 2751. 32927614 10.3390/nu12092751 PMC7551151

[33] C. Wang , C. Sun , W. Lu , K. Gul , A. Mata , Y. Fang , Compr Rev Food Sci Food Saf 2020, 19, 2955. 33337053 10.1111/1541-4337.12621

[34] F. Bot , D. Cossuta , J. A. O'Mahony , Trends Food Sci. Technol. 2021, 111, 261.

[35] X. Li , Y. Xin , Y. Mo , P. Marozik , T. He , H. Guo , Molecules 2022, 27, 523. 35056839 10.3390/molecules27020523 PMC8781140

[36] N. Filipczak , J. Pan , S. S. K. Yalamarty , V. P. Torchilin , Adv Drug Deliv Rev 2020, 156, 4. 32593642 10.1016/j.addr.2020.06.022

[37] Z. F. Yang , R. Xiao , F. J. Luo , Innov. Food Sci. Emerg. Technol. 2020, 66, 102499.

[38] E. K. Lim , D. Bowles , Curr. Opin. Biotechnol. 2012, 23, 271. 22221831 10.1016/j.copbio.2011.12.008

[39] L. Yang , K. S. Wen , X. Ruan , Molecules 2018, 23, 762. 29584636 10.3390/molecules23040762 PMC6017249

[40] G. L. Hostetler , R. A. Ralston , S. J. Schwartz , Adv Nutr 2017, 8, 423. 28507008 10.3945/an.116.012948 PMC5421117

[41] S. A. Heleno , A. Martins , M. J. R. P. Queiroz , I. C. F. R. Ferreira , Food Chem 2015, 173, 501. 25466052 10.1016/j.foodchem.2014.10.057

[42] C. G. Fraga , K. D. Croft , D. O. Kennedy , F. A. Tomás‐Barberán , Food Funct. 2019, 10, 514. 30746536 10.1039/c8fo01997e

[43] C. Rodríguez‐García , C. Sánchez‐Quesada , E. Toledo , M. Delgado‐Rodríguez , J. J. Gaforio , Molecules 2019, 24, 917. 30845651 10.3390/molecules24050917 PMC6429205

[44] V. Jarosova , O. Vesely , P. Marsik , J. D. Jaimes , K. Smejkal , P. Kloucek , J. Havlik , Molecules 2019, 24, 1155. 30909544 10.3390/molecules24061155 PMC6471231

[45] L. González‐Cofrade , B. de las Heras , L. Apaza Ticona , O. M. Palomino , Planta Med. 2019, 85, 1304. 31234214 10.1055/a-0953-6738

[46] E. R. Soto , F. Rus , H. Li , C. Garceau , J. Chicca , M. Elfawal , D. Gazzola , M. K. Nielsen , J. F. Urban , R. V. Aroian , G. R. Ostroff , Foods 2021, 10, 1207. 34071798 10.3390/foods10061207 PMC8228553

[47] X. Zheng , F. Wu , X. Lin , L. Shen , Y. Feng , Drug Delivery 2018, 25, 398. 29378456 10.1080/10717544.2018.1431980 PMC6058676

[48] J. Navarro del Hierro , T. Herrera , T. Fornari , G. Reglero , D. Martin , J Funct Foods 2018, 40, 484.

[49] J. Sharifi‐Rad , A. Sureda , G. Tenore , M. Daglia , M. Sharifi‐Rad , M. Valussi , R. Tundis , M. Sharifi‐Rad , M. Loizzo , A. Ademiluyi , R. Sharifi‐Rad , S. Ayatollahi , M. Iriti , Molecules 2017, 22, 70. 28045446 10.3390/molecules22010070 PMC6155610

[50] S. Mitra , S. Paul , S. Roy , H. Sutradhar , T. Bin Emran , F. Nainu , M. U. Khandaker , M. Almalki , P. Wilairatana , M. S. Mubarak , Molecules 2022, 27, 555. 35056870 10.3390/molecules27020555 PMC8779769

[51] A. M. Macan , T. G. Kraljević , S. Raić‐Malić , Antioxidants 2019, 8, 247. 31357509 10.3390/antiox8080247 PMC6721080

[52] Z. Ma , M. Yang , M. F. Foda , K. Zhang , S. Li , H. Liang , Y. Zhao , H. Han , ACS Nano 2022, 16, 17389. 36166666 10.1021/acsnano.2c08446

[53] S. M. T. Gharibzahedi , S. M. Jafari , Trends Food Sci. Technol. 2017, 62, 119.

[54] Q. Luan , H. Zhang , C. Chen , F. Jiang , Y. Yao , Q. Deng , K. Zeng , H. Tang , F. Huang , ACS Nano 2022, 16, 2198. 35142211 10.1021/acsnano.1c08244

[55] S. Asgari , A. Pourjavadi , T. R. Licht , A. Boisen , F. Ajalloueian , Adv Drug Deliv Rev 2020, 161, 1. 32702378 10.1016/j.addr.2020.07.014

[56] C. Liu , J. Zheng , X. Ou , Y. Han , Front Microbiol 2021, 12, 722052. 34721321 10.3389/fmicb.2021.722052 PMC8548880

[57] P. M. Reque , A. Brandelli , Trends Food Sci. Technol. 2021, 114, 1.

[58] Y. Luo , C. De Souza , M. Ramachandran , S. Wang , H. Yi , Z. Ma , L. Zhang , K. Lin , J. Control. Release 2022, 352, 371. 36309096 10.1016/j.jconrel.2022.10.030

[59] R. Abid , H. Waseem , J. Ali , S. Ghazanfar , G. Muhammad Ali , A. M. Elasbali , S. H. Alharethi , J. Fungi 2022, 8, 444. 10.3390/jof8050444 PMC9147304 35628700

[60] Z. P. Zou , Y. Du , T. T. Fang , Y. Zhou , B. C. Ye , Cell Host Microbe 2023, 31, 199. 36758520 10.1016/j.chom.2022.12.004

[61] M. R. Kathiriya , Y. V. Vekariya , S. Hati , Probiotics Antimicrob Proteins 2023, 15, 1032. 37347421 10.1007/s12602-023-10104-3

[62] C. G. Awuchi , S. Morya , T. A. Dendegh , C. O. R. Okpala , M. Korzeniowska , Bioresour. Technol. Rep. 2022, 19, 101088.

[63] C. Dima , E. Assadpour , S. Dima , S. M. Jafari , Compr Rev Food Sci Food Saf 2020, 19, 954. 33331687 10.1111/1541-4337.12547

[64] G. L. Amidon , H. Lennernäs , V. P. Shah , J. R. Crison , Pharm. Res. 1995, 12, 413. 7617530 10.1023/a:1016212804288

[65] D. J. McClements , L. Zou , R. Zhang , L. Salvia‐Trujillo , T. Kumosani , H. Xiao , Compr Rev Food Sci Food Saf 2015, 14, 824.

[66] X. Li , X. Jiang , J. Sun , C. Zhu , W. Bai , Food Chem Toxicol 2018, 119, 342. 29452191 10.1016/j.fct.2018.02.024

[67] M. Alongi , M. Anese , J Funct Foods 2021, 81, 104466.

[68] M. Potier , L. Tea , L. Benyahia , T. Nicolai , F. Renou , Macromolecules 2020, 53, 10514.

[69] F. Seidi , M. K. Yazdi , M. Jouyandeh , S. Habibzadeh , M. T. Munir , H. Vahabi , B. Bagheri , N. Rabiee , P. Zarrintaj , M. R. Saeb , Carbohyd. Polym. 2022, 275, 118624. 10.1016/j.carbpol.2021.118624 34742405

[70] Y. Yang , L. Xu , J. Wang , Q. Meng , S. Zhong , Y. Gao , X. Cui , Carbohyd. Polym. 2022, 283, 119161. 10.1016/j.carbpol.2022.119161 35153030

[71] S. Xiang , B. Hammer , K. Kremer , K. Müllen , T. Weil , Prog Polym Sci 2022, 124, 101489.

[72] C. N. Pace , G. R. Grimsley , J. M. Scholtz , J. Biol. Chem. 2009, 284, 13285. 19164280 10.1074/jbc.R800080200 PMC2679426

[73] T. A. Wani , A. G. Shah , S. M. Wani , I. A. Wani , F. A. Masoodi , N. Nissar , M. A. Shagoo , Crit. Rev. Food Sci. Nutr. 2015, 56, 2431. 10.1080/10408398.2013.845814 25603446

[74] J. J. Chai , P. Jiang , P. J. Wang , Y. M. Jiang , D. Li , W. E. Bao , B. X. Liu , B. Liu , L. Y. Zhao , W. Norde , Q. P. Yuan , F. Z. Ren , Y. Li , Trends Food Sci. Technol. 2018, 78, 144.

[75] S. Shrivastava , C. D. Kaur , Drug Delivery Transl. Res. 2022, 13, 658. 10.1007/s13346-022-01230-6 35978260

[76] A. J. Meléndez‐Martínez , P. Esquivel , D. B. Rodriguez‐Amaya , Food Res Int 2023, 169, 112773. 37254377 10.1016/j.foodres.2023.112773

[77] Q. Zhang , Z. Cheng , Y. Wang , L. Fu , Crit. Rev. Food Sci. Nutr. 2020, 61, 3589. 32814438 10.1080/10408398.2020.1803199

[78] C. Sun , S. Wang , L. Yang , H. Song , Food Biosci. 2023, 52, 102476.

[79] S. Wang , S. Ahmadi , R. Nagpal , S. Jain , S. P. Mishra , K. Kavanagh , X. Zhu , Z. Wang , D. A. McClain , S. B. Kritchevsky , D. W. Kitzman , H. Yadav , GeroScience 2020, 42, 333. 31814084 10.1007/s11357-019-00137-4 PMC7031475

[80] M. Molaee Parvarei , M. R. Fazeli , A. M. Mortazavian , S. Sarem Nezhad , S. A. Mortazavi , A. A. Golabchifar , N. Khorshidian , Food Res Int 2021, 140, 110030. 33648258 10.1016/j.foodres.2020.110030

[81] K. Rajendran , A. Karthikeyan , U. M. Krishnan , Int. J. Biol. Macromol. 2022, 208, 627. 35341885 10.1016/j.ijbiomac.2022.03.121

[82] W. H. Wijaya , A. R. Patel , A. D. Setiowati , P. Van der Meeren , Trends Food Sci. Technol. 2017, 68, 56.

[83] F. Liu , D. J. McClements , C. Ma , X. Liu , Annu. Rev. Food Sci. Technol. 2023, 14, 35. 36972160 10.1146/annurev-food-060721-023522

[84] Y. Zhou , S. P. Petrova , K. J. Edgar , Carbohyd. Polym. 2021, 274, 118662. 10.1016/j.carbpol.2021.118662 34702481

[85] T. F. Wu , C. M. Liu , X. T. Hu , Food Chem. 2022, 372, 131332. 34818742 10.1016/j.foodchem.2021.131332

[86] M. Gaber , W. Medhat , M. Hany , N. Saher , J.‐Y. Fang , A. Elzoghby , J. Control. Release 2017, 254, 75. 28365294 10.1016/j.jconrel.2017.03.392

[87] L. Bugnicourt , C. Ladaviere , J. Control. Release 2017, 256, 121. 28414148 10.1016/j.jconrel.2017.04.018

[88] X. T. Le , L. E. Rioux , S. L. Turgeon , Adv. Colloid Interface Sci. 2017, 239, 127. 27318757 10.1016/j.cis.2016.04.006

[89] N. Bezssonoff , H. Leroux , Nature 1945, 156, 474.

[90] N. Mehta , J. Priya , P. Kumar , A. K. Verma , P. Umaraw , S. K. Khatkar , A. B. Khatkar , D. Pathak , U. Kaka , A. Q. Sazili , Foods 2022, 11, 2973. 36230050 10.3390/foods11192973 PMC9564298

[91] E. Austin , A. N. Geisler , J. Nguyen , I. Kohli , I. Hamzavi , H. W. Lim , J. Jagdeo , J Am Acad Dermatol 2021, 84, 1219. 33640508 10.1016/j.jaad.2021.02.048 PMC8887026

[92] A. Rezaei , F. Rafieian , S. Akbari‐Alavijeh , M. S. Kharazmi , S. M. Jafari , Adv. Colloid Interface Sci. 2022, 307, 102728. 35843031 10.1016/j.cis.2022.102728

[93] E. Pinilla‐Peñalver , B. García‐Béjar , A. M. Contento , Á. Ríos , Food Chem 2022, 386, 132766. 35349896 10.1016/j.foodchem.2022.132766

[94] C. Pedizzi , L. Regueiro , I. Rodriguez‐Verde , J. M. Lema , M. Carballa , Bioresour. Technol. 2016, 211, 765. 27020398 10.1016/j.biortech.2016.03.085

[95] J. Lee , N. Koo , D. B. Min , Compr Rev Food Sci Food Saf 2004, 3, 21. 33430557 10.1111/j.1541-4337.2004.tb00058.x

[96] I. G. Radzievska , O. P. Melnyk , Sci. Innov. 2015, 11, 30.

[97] H. Debelo , M. Li , M. G. Ferruzzi , Curr Opin Food Sci 2020, 32, 90.

[98] A. Rawson , A. Patras , B. K. Tiwari , F. Noci , T. Koutchma , N. Brunton , Food Res Int 2011, 44, 1875.

[99] G. V. Barbosa‐Cánovas , F. Donsì , S. Yildiz , K. Candogan , P. R. Pokhrel , A. Y. Guadarrama‐Lezama , Food Eng. Rev. 2022, 14, 63.

[100] W. Leonard , P. Zhang , D. Ying , B. Adhikari , Z. Fang , Biotechnol. Adv. 2021, 49, 107763. 33961978 10.1016/j.biotechadv.2021.107763

[101] H. Kawakami , M. Tanaka , K. Tatsumi , S. '. Dosako , Int. Dairy J. 1992, 2, 287.

[102] Y. Xu , N. Shrestha , V. Préat , A. Beloqui , J. Control. Release 2020, 322, 486. 32276004 10.1016/j.jconrel.2020.04.006

[103] I. Sensoy , Crit. Rev. Food Sci. Nutr. 2014, 54, 902. 24499069 10.1080/10408398.2011.619016

[104] C. Li , W. W. Yu , P. Wu , X. D. Chen , Trends Food Sci. Technol. 2020, 96, 114.

[105] I. Sensoy , Curr. Res. Food Sci. 2021, 4, 308. 34027433 10.1016/j.crfs.2021.04.004 PMC8134715

[106] R. J. Mu , J. S. Chen , Trends Food Sci. Technol. 2023, 132, 121. 10.1016/j.tifs.2022.12.012 PMC9796359 36594074

[107] P. Subramanian , Foods 2021, 10, 1362. 34208328 10.3390/foods10061362 PMC8231213

[108] C. Li , Y. Hu , S. Li , X. Yi , S. Shao , W. Yu , E. Li , Food Sci Hum Wellness 2023, 12, 351. 10.1016/j.fshw.2022.10.036 PMC9671704 38620800

[109] Y. F. Zhang , Y. Chen , J. S. Chen , Curr Opin Food Sci 2022, 43, 237.

[110] J. S. Chen , Trends Food Sci. Technol. 2015, 45, 222.

[111] X. Ni , Z. Tan , C. Ding , C. Zhang , L. Song , S. Yang , M. Liu , R. Jia , C. Zhao , L. Song , W. Liu , Q. Zhou , T. Gong , X. Li , Y. Tai , W. Zhu , T. Shi , Y. Wang , J. Xu , B. Zhen , J. Qin , Nat. Commun. 2019, 10, 39. 30604760 10.1038/s41467-018-07960-x PMC6318339

[112] F. Kong , R. P. Singh , J. Food Sci. 2008, 73, R67. 18577009 10.1111/j.1750-3841.2008.00766.x

[113] K. Kalantar‐zadeh , N. Ha , J. Z. Ou , K. J. Berean , ACS Sens. 2017, 2, 468. 28723186 10.1021/acssensors.7b00045

[114] T. Stalder , T. Zaiter , W. El‐Basset , R. Cornu , H. Martin , M. Diab‐Assaf , A. Béduneau , Toxicology. 2022, 481, 153353. 36257551 10.1016/j.tox.2022.153353

[115] J. Beumer , H. Clevers , Nat. Rev. Mol. Cell Biol. 2021, 22, 39. 32958874 10.1038/s41580-020-0278-0

[116] N. Shi , N. Li , X. Duan , H. Niu , Mil Med Res 2017, 4, 1. 28465831 10.1186/s40779-017-0122-9 PMC5408367

[117] H. S. Kim , D. Y. Lee , J Ind Eng Chem 2021, 102, 122.

[118] D. A. Subramanian , R. Langer , G. Traverso , J. Nanobiotechnol. 2022, 20, 1. 10.1186/s12951-022-01539-x PMC9356434 35933341

[119] S. F. Phillips , A. M. Stephen , Nutrition Today 1981, 16, 4.

[120] K. Feng , Y. S. Wei , T. G. Hu , R. J. Linhardt , M. H. Zong , H. Wu , Trends Food Sci. Technol. 2020, 102, 203.

[121] M. L. Occhiutto , F. R. Freitas , R. C. Maranhao , V. P. Costa , Pharmaceutics 2012, 4, 252. 24300231 10.3390/pharmaceutics4020252 PMC3834913

[122] K. L. Leiby , M. S. B. Raredon , L. E. Niklason , Compr Physiol 2020, 10, 415. 32163210 10.1002/cphy.c190026 PMC7366783

[123] T. Pradhan‐Sundd , R. Vats , J. O. Russell , S. Singh , A. A. Michael , L. Molina , S. Kakar , P. Cornuet , M. Poddar , S. C. Watkins , K. N. Nejak‐Bowen , S. P. Monga , P. Sundd , Gastroenterology 2018, 155, 1218. 29964040 10.1053/j.gastro.2018.06.048 PMC6174089

[124] S. Zhang , L. Gan , F. Cao , H. Wang , P. Gong , C. Ma , L. Ren , Y. Lin , X. Lin , Brain Res. Bull. 2022, 190, 69. 36162603 10.1016/j.brainresbull.2022.09.017

[125] N. N. Bhuyan , A. Joardar , B. P. Bag , H. Chakraborty , A. Mishra , J. Mol. Liq. 2021, 344, 117752.

[126] H. Li , S.‐L. Chang , T.‐R. Chang , Y. You , X.‐D. Wang , L.‐W Wang , X.‐F. Yuan , M.‐H. Tan , P.‐D. Wang , P.‐W. Xu , W‐B. Gao , Q.‐S. Zhao , B. Zhao , J. Mol. Liq. 2021, 334, 116070.

[127] F. Amani , A. Rezaei , M. S. Kharazmi , S. M. Jafari , Colloids Surf. A 2022, 649, 129454.

[128] Y. J. Baek , E. W. Jeong , H. G. Lee , Colloids Surf., B 2023, 224, 113205. 10.1016/j.colsurfb.2023.113205 36801525

[129] P. Wu , L. Chen , M. Chen , B. S. Chiou , F. Xu , F. Liu , F. Zhong , Food Chem. 2023, 414, 135685. 36809726 10.1016/j.foodchem.2023.135685

[130] I. Khalil , W. A. Yehye , A. E. Etxeberria , A. A. Alhadi , S. M. Dezfooli , N. B. M. Julkapli , W. J. Basirun , A. Seyfoddin , Antioxidants 2019, 9, 24. 31888023 10.3390/antiox9010024 PMC7022483

[131] A. Jash , S. S. Rizvi , Innovative Food Sci. Emerging Technol. 2022, 79, 103030. 10.1016/j.ifset.2022.103031 PMC9574788 36276609

[132] X. Xu , W. Zhao , Y. Ye , W. Cui , L. Dong , Y. Yao , K. Li , J. Han , W. Liu , J. Agric. Food Chem. 2021, 69, 9395. 34344151 10.1021/acs.jafc.1c02817

[133] Y. Yuan , Y. Liu , Y. He , B. Zhang , L. Zhao , S. Tian , Q. Wang , S. Chen , Z. Li , S. Liang , G. Hou , B. Liu , Y. Li , Biomaterials 2022, 287, 121613. 35700621 10.1016/j.biomaterials.2022.121613

[134] I. Frosi , L. Ferron , R. Colombo , A. Papetti , Crit. Rev. Food Sci. Nutr. 2022, 64, 5700. 36533404 10.1080/10408398.2022.2157371

[135] Y. Chen , K. Tai , P. Ma , J. Su , W. Dong , Y. Gao , L. Mao , J. Liu , F. Yuan , Food Chem 2021, 347, 128978. 33444890 10.1016/j.foodchem.2020.128978

[136] J. Fu , L. Song , J. Guan , C. Sun , D. Zhou , B. Zhu , Food Chem 2021, 338, 128089. 33091980 10.1016/j.foodchem.2020.128089

[137] K. Szymandera‐Buszka , K. Waszkowiak , A. Kaczmarek , A. Zaremba , LWT – Food Sci. Technol. 2021, 137, 110424.

[138] Y. Cao , Z. Zang , L. Zhang , G. Han , Q. Yu , L. Han , Int. J. Biol. Macromol. 2023, 250, 126269. 37567542 10.1016/j.ijbiomac.2023.126269

[139] H. T. Ong , D. D. Suppiah , N. M. Julkapli , Colloids Surf. A 2020, 606, 125371.

[140] S. Amjadi , H. Almasi , H. Hamishehkar , M. Alizadeh Khaledabad , L.‐T. Lim , Food Chem 2022, 373, 131403. 34710692 10.1016/j.foodchem.2021.131403

[141] A. Thakur , M. K. Jaiswal , C. W. Peak , J. K. Carrow , J. Gentry , A. Dolatshahi‐Pirouz , A. K. Gaharwar , Nanoscale 2016, 8, 12362. 27270567 10.1039/c6nr02299e

[142] A. Mihaly Cozmuta , M. A. K. Purbayanto , A. Jastrzebska , A. Peter , C. Nicula , A. Uivarasan , L. Mihaly Cozmuta , Food Hydrocolloid. 2023, 142, 108808. 10.1016/j.fochx.2024.101758 PMC11639332 39679380

[143] M. Wang , Y. Liu , X. Zhang , L. Luo , L. Li , S. Xing , Y. He , W. Cao , R. Zhu , D. Gao , J. Mater. Chem. B 2017, 5, 2161. 32263689 10.1039/c7tb00258k

[144] S. Salazar Sandoval , E. Cortés‐Adasme , E. Gallardo‐Toledo , I. Araya , F. Celis , N. Yutronic , P. Jara , M. J. Kogan , Pharmaceutics 2206, 14, 2206. 10.3390/pharmaceutics14102206 PMC9611720 36297642

[145] L. Tang , Y. Sun , P. Ge , L. Chen , P. C. K. Cheung , Z. Ding , J. Fang , Int. J. Biol. Macromol. 2022, 209, 1771. 35472365 10.1016/j.ijbiomac.2022.04.147

[146] S. M. T. Gharibzahedi , C. Hernández‐Ortega , J. Welti‐Chanes , P. Putnik , F. J. Barba , K. Mallikarjunan , Z. Escobedo‐Avellaneda , S. Roohinejad , Food Hydrocolloid. 2019, 87, 307.

[147] H. Ding , P. Tan , S. Fu , X. Tian , H. Zhang , X. Ma , Z. Gu , K. Luo , J. Control. Release 2022, 348, 206. 35660634 10.1016/j.jconrel.2022.05.056

[148] S. G. Nugent , Gut 2001, 48, 571.11247905

[149] G. Zhang , W. Han , P. Zhao , Z. Wang , M. Li , X. Sui , Y. Liu , B. Tian , Z. He , Q. Fu , Nanoscale 2023, 15, 1937. 36625215 10.1039/d2nr04968f

[150] Y. Dai , C. Xu , X. Sun , X. Chen , Chem. Soc. Rev. 2017, 46, 3830. 28516983 10.1039/c6cs00592f PMC5521825

[151] K. Xiong , L. Y. Zhou , J. Y. Wang , A. G. Ma , D. Fang , L. Xiong , Q. J. Sun , Trends Food Sci. Technol. 2020, 96, 102.

[152] K. Omori , H. Miyakawa , A. Watanabe , Y. Nakayama , Y. Lyu , N. Ichikawa , H. Sasaki , S. Shibata , Nutrients 2021, 13, 152. 33466274 10.3390/nu13010152 PMC7824761

[153] A. Libánská , E. Randárová , F. Lager , G. Renault , D. Scherman , T. S. Etrych , Pharmaceutics 2020, 12, 700. 32722403 10.3390/pharmaceutics12080700 PMC7465548

[154] W.‐L. Chen , F. Li , Y. Tang , S‐D. Yang , J.‐Z Li , Z.‐Q. Yuan , Y. Liu , X.‐F. Zhou , C. Liu , X.‐N. Zhang , Int. J. Nanomed. 2017, 12, 4241. 10.2147/IJN.S129748 PMC5473598 28652730

[155] A. M. Leon , W. T. Medina , D. J. Park , J. M. Aguilera , J. Food Eng. 2016, 188, 1.

[156] D.‐W Kim , H.‐S. Jeong , E. Kim , H. Lee , C.‐H. Choi , S.‐J. Lee , J. Control. Release 2022, 347, 508. 35597403 10.1016/j.jconrel.2022.05.028

[157] M. Ezzat , R. Latif , S. S. Badawy , F. A. Torad , J Drug Deliv Sci Technol 2021, 65, 102657.

[158] M. Jimenez , V. Gil , M. Martinez‐Cutillas , N. Mañé , D. Gallego , Br. J. Pharmacol. 2017, 174, 2805. 28631296 10.1111/bph.13918 PMC5554320

[159] M. K. Herbert , R. Weis , P. Holzer , N. Roewer , Anesth. Analg. 2005, 100, 120. 15616065 10.1213/01.ANE.0000139352.54676.18

[160] X. Chen , D. Liang , W. Sun , X. Shou , L. Shang , X. Shen , Chem. Eng. J. 2023, 458, 141428.

[161] X. Shi , N. Fan , G. Zhang , J. Sun , Z. He , J. Li , Pharm. Dev. Technol. 2020, 25, 472. 31909684 10.1080/10837450.2019.1709502

[162] J. Yang , G. Zhang , X. Yang , M. Peng , S. Ge , S. Tan , Z. Wen , Y. Wang , S. Wu , Y. Liang , J. An , K. Zhang , J. Liu , J. Shi , Z. Zhang , Chem. Eng. J. 2022, 446, 137204.

[163] Z. Zhang , F. Chen , R. Zhang , Z. Deng , D. J. McClements , J. Agric. Food Chem. 2016, 64, 9616. 27966930 10.1021/acs.jafc.6b04644

[164] Z. Zhang , R. Zhang , D. J. McClements , Food Hydrocolloid. 2017, 67, 85.

[165] Y. Yang , H. Yu , X. Zhou , Z. Zhou , Mol. Catal. 2020, 486, 110871.

[166] H. Zhou , Z. Fan , J. Deng , P. K. Lemons , D. C. Arhontoulis , W. B. Bowne , H. Cheng , Nano Lett. 2016, 16, 3268. 27057591 10.1021/acs.nanolett.6b00820

[167] M. K. Shukla , C. Behera , S. Chakraborty , K. K. Sandha , A. Goswami , P. N. Gupta , J Drug Deliv Sci Technol 2022, 71, 103366.

[168] C. Menzel , A. Bernkop‐Schnürch , Adv Drug Deliv Rev 2018, 124, 164. 29079537 10.1016/j.addr.2017.10.004

[169] Z. Yang , L. Chen , D. J. McClements , C. Qiu , C. Li , Z. Zhang , M. Miao , Y. Tian , K. Zhu , Z. Jin , Food Hydrocolloid. 2022, 124, 107218.

[170] Z. Zhang , R. Zhang , L. Chen , Q. Tong , D. J. McClements , Eur. Polym. J. 2015, 72, 698.

[171] C. Xu , S. Chen , C. Chen , Y. Ming , J. Du , J. Mu , F. Luo , D. Huang , N. Wang , Z. Lin , Z. Weng , Int. J. Pharm. 2022, 623, 121884. 35661797 10.1016/j.ijpharm.2022.121884

[172] R. Wang , Z. Zhang , B. Liu , J. Xue , F. Liu , T. Tang , W. Liu , F. Feng , W. Qu , Biomater. Sci. 2021, 9, 3621. 34008587 10.1039/d0bm02221g

[173] J. C. Leroux , Nat. Nanotechnol. 2007, 2, 679. 18654405 10.1038/nnano.2007.339

[174] A. Wagh , J. Singh , S. Qian , B. Law , Nanomedicine 2013, 9, 449. 23178287 10.1016/j.nano.2012.10.009

[175] Y. S. Zheng , W. F. Guo , L. W. Hu , Z. K. Xiao , X. Z. Yang , Z. Y. Cao , J. Cao , ACS Appl. Mater. Interfaces 2023, 15, 22843. 37133278 10.1021/acsami.3c00469

[176] S. S. Chen , Q. M. Li , H. L. Li , L. Q. Yang , J. Z. Yi , M. Q. Xie , L. M. Zhang , Mater. Sci. Eng. C 2020, 109, 110636. 10.1016/j.msec.2020.110636 32228909

[177] K. Zhao , Y. Xie , X. Lin , W. Xu , Int. J. Nanomed. 2022, 17, 4579. 10.2147/IJN.S359118 PMC9527817 36199476

[178] C. Bao , B. Liu , B. Li , J. Chai , L. Zhang , L. Jiao , D. Li , Z. Yu , F. Ren , X. Shi , Y. Li , Nano Lett. 2020, 20, 1352. 31904988 10.1021/acs.nanolett.9b04841

[179] P. Paone , P. D. Cani , Gut 2020, 69, 2232. 32917747 10.1136/gutjnl-2020-322260 PMC7677487

[180] S. P. Bandi , S. Bhatnagar , V. V. K. Venuganti , Acta Biomater. 2021, 119, 13. 33141051 10.1016/j.actbio.2020.10.031

[181] T. L. Carlson , J. Y. Lock , R. L. Carrier , Annu. Rev. Biomed. Eng. 2018, 20, 197. 29865871 10.1146/annurev-bioeng-062117-121156 PMC6463277

[182] Y. Zhang , M. Li , G. Du , X. Chen , X. Sun , Adv Drug Deliv Rev 2021, 177, 113928. 34411689 10.1016/j.addr.2021.113928

[183] J.‐S Lee , J. W Suh , E. S. Kim , H. G. Lee , J. Agric. Food Chem. 2017, 65, 8930. 28933847 10.1021/acs.jafc.7b03300

[184] E. S. Armengol , F. Laffleur , Int. J. Pharm. 2021, 592, 120016. 33176200 10.1016/j.ijpharm.2020.120016

[185] X. Jin , S. Asghar , M. Zhang , Z. P. Chen , L. Huang , Q. N. Ping , Y. Y. Xiao , Colloids Surf., B 2018, 172, 655. 10.1016/j.colsurfb.2018.09.025 30243219

[186] Y. Zaiki , L. Y. Lim , T. W. Wong , Int. Mater. Rev. 2023, 68, 121.

[187] D. Xie , X. Zhou , B. Xiao , L. Duan , Z. Zhu , Biomolecules 2022, 12, 1263. 36139101 10.3390/biom12091263 PMC9496219

[188] N. Ijssennagger , R. V. D. Meer , S. W. C. V. Mil , Trends Mol. Med. 2016, 22, 190. 26852376 10.1016/j.molmed.2016.01.002

[189] I. Shahzadi , A. Dizdarevic , N. A. Efiana , B. Matuszczak , A. Bernkop‐Schnürch , J. Colloid Interface Sci. 2018, 531, 253. 30036849 10.1016/j.jcis.2018.07.057

[190] F. Barreau , J. P. Hugot , Curr. Opin. Microbiol. 2014, 17, 91. 24440560 10.1016/j.mib.2013.12.003

[191] E. Cocucci , J. Y. Kim , Y. Bai , N. Pabla , Clin. Pharmacol. Ther. 2016, 101, 121. 27804130 10.1002/cpt.545

[192] J. Reinholz , K. Landfester , V. Mailänder , Drug Delivery 2018, 25, 1694. 30394120 10.1080/10717544.2018.1501119 PMC6225504

[193] S. Majumdar , A. K. Mitra , Expert Opin. Drug Del. 2006, 3, 511. 10.1517/17425247.3.4.511 16822226

[194] Y. Xu , B.‐W. Zhu , X. Li , Y.‐F. Li , X.‐M Ye , J.‐N. Hu , Biomaterials 2022, 280, 121077. 34890974 10.1016/j.biomaterials.2021.121077

[195] S. Tie , W. Su , Y. Chen , S. Wu , H. Wu , Y. Song , S. Fei , M. Tan , Chem. Eng. J. 2022, 441, 136095.

[196] M. Zu , D. Xie , B. S. B. Canup , N. Chen , Y. Wang , R. Sun , Z. Zhang , Y. Fu , F. Dai , B. Xiao , Biomaterials 2021, 279, 121178. 34656857 10.1016/j.biomaterials.2021.121178

[197] W. Chen , Q. Zhang , B. T. Luk , R. H. Fang , Y. Liu , W. Gao , L. Zhang , Nanoscale 2016, 8, 10364. 27139582 10.1039/c6nr00535g PMC4866884

[198] Z. Kang , Q. Liu , Z. Zhang , Y. Zheng , C. Wang , Z. Pan , Q. Li , Y. Liu , L. Shi , Adv. Healthcare Mater. 2022, 11, 2200371. 10.1002/adhm.202200371 35460333

[199] S. Arayachukiat , J. Seemork , P. Pan‐In , K. Amornwachirabodee , N. Sangphech , T. Sansureerungsikul , K. Sathornsantikun , C. Vilaivan , K. Shigyou , P. Pienpinijtham , T. Vilaivan , T. Palaga , W. Banlunara , T. Hamada , S. Wanichwecharungruang , Nano Lett. 2015, 15, 3370. 25849219 10.1021/acs.nanolett.5b00696

[200] C. Lv , X. Gu , H. Li , Y. Zhao , D. Yang , W. Yu , D. Han , J. Li , W. Tan , ACS Nano 2020, 14, 14616. 32897687 10.1021/acsnano.0c03105

[201] S. H. Lee , Intest Res 2015, 13, 11. 25691850 10.5217/ir.2015.13.1.95 PMC4316230

[202] Z. M. Slifer , A. T. Blikslager , Int. J. Mol. Sci. 2020, 21, 972.32024112

[203] A. Banerjee , R. Chen , S. Arafin , S. Mitragotri , J. Control. Release 2019, 297, 71. 30707901 10.1016/j.jconrel.2019.01.037

[204] H. J. R. Lemmer , J. H. Hamman , Expert Opin. Drug Del. 2012, 10, 103. 10.1517/17425247.2013.745509 23163247

[205] J. H. Lee , A. Sahu , W. I. Choi , J. Y. Lee , G. Tae , Biomaterials 2016, 103, 160. 27380442 10.1016/j.biomaterials.2016.06.059

[206] X. Li , S. M. Jafari , F. Zhou , H. Hong , X. Jia , X. Mei , G. Hou , Y. Yuan , B. Liu , S. Chen , Y. Gong , H. Yan , R. Chang , J. Zhang , F. Ren , Y. Li , Biomaterials 2023, 294, 121995. 36641813 10.1016/j.biomaterials.2023.121995

[207] J. McCright , A. Ramirez , M. Amosu , A. Sinha , A. Bogseth , K. Maisel , Pharmaceutics 2021, 13, 1755. 34834170 10.3390/pharmaceutics13111755 PMC8619927

[208] W. Liu , Y. Han , X. Xin , L. Chen , Y. Liu , C. Liu , X. Zhang , M. Jin , J. Jin , Z. Gao , W. Huang , J. Nanobiotechnol. 2022, 20, 281. 10.1186/s12951-022-01460-3 PMC9199201 35705976

[209] Y.‐B. Miao , K.‐H. Chen , C.‐T. Chen , F.‐L. Mi , Y.‐J Lin , Y. Chang , C.‐S. Chiang , J.‐T. Wang , K.‐J. Lin , H.‐W. Sung , Adv. Mater. 2021, 33, 2100701. 10.1002/adma.202100701 34270814

[210] T. A. Doan , T. Forward , B. A. J Tamburini , Cell. Mol. Life Sci. 2022, 79, 275. 35505125 10.1007/s00018-022-04303-4 PMC9063628

[211] X. Ma , N. Gong , L. Zhong , J. Sun , X.‐J. Liang , Biomaterials 2016, 97, 10. 27155363 10.1016/j.biomaterials.2016.04.026

[212] A. Jhaveri , V. Torchilin , Expert Opin. Drug Del. 2015, 13, 49. 10.1517/17425247.2015.1086745 26358656

[213] K. Song , D. C. Nguyen , T. Luu , O. Yazdani , D. Roy , P. S. Stayton , S. H. Pun , J. Control. Release 2023, 356, 232. 36878319 10.1016/j.jconrel.2023.03.004 PMC10693254

[214] Y. Xie , F. Hu , D. Xiang , H. Lu , W. Li , A. Zhao , L. Huang , R. Wang , Drug Metab Rev 2020, 52, 139. 32116054 10.1080/03602532.2020.1718691

[215] M.‐J. Liu , J‐Y. Yang , Z.‐H. Yan , S. Hu , J‐Q. Li , Z.‐X. Xu , Y.‐P. Jian , Clin. Nutr. 2022, 41, 2333. 36113229 10.1016/j.clnu.2022.08.029

[216] H. Nagao‐Kitamoto , S. Kitamoto , P. Kuffa , N. Kamada , Intest Res 2016, 14, 127. 27175113 10.5217/ir.2016.14.2.127 PMC4863046

[217] Y. Wei , J. Xu , S. Miao , K. Wei , L. Peng , Y. Wang , X. Wei , Crit. Rev. Food Sci. Nutr. 2022, 63, 7598. 35266837 10.1080/10408398.2022.2048291

[218] D. Zhong , D. Zhang , W. Chen , J. He , C. Ren , X. Zhang , N. Kong , W. Tao , M. Zhou , Sci. Adv. 2021, 7, eabi9265. 34818040 10.1126/sciadv.abi9265 PMC8612690

[219] Q. Gu , Y. Yin , X. Yan , X. Liu , F. Liu , D. J. McClements , Adv. Colloid Interface Sci. 2022, 309, 102781. 36209686 10.1016/j.cis.2022.102781

[220] A. Abbasi , N. Hajipour , P. Hasannezhad , A. Baghbanzadeh , L. Aghebati‐Maleki , Crit. Rev. Food Sci. Nutr. 2020, 62, 3345. 33356449 10.1080/10408398.2020.1865260

[221] Y. Ren , L. Nie , C. Luo , S. Zhu , X. Zhang , Int. J. Nanomed. 2022, 17, 6639. 10.2147/IJN.S390102 PMC9793785 36582460

[222] R. Khursheed , M. Gulati , S. Wadhwa , S. Vishwas , D. S. Sharma , L. Corrie , A. Alam , S. M. Alnasser , F. F. Aba Alkhayl , Z. Parveen , S. Nammi , D. K. Chellappan , G. Gupta , F. Zacconi , A. Steel , J. Adams , N. K. Jha , K. Dua , S. K. Singh , Chem. Biol. Interact. 2022, 368, 110223. 36283466 10.1016/j.cbi.2022.110223

[223] A. Xie , H. Ji , Z. Liu , Y. Wan , X. Zhang , H. Xiong , S.‐P. Nie , H. Wan , ACS Nano 2023, 17, 14775. 37477584 10.1021/acsnano.3c02914

[224] K. Yang , X. Wang , R. Huang , H. Wang , P. Lan , Y. Zhao , Adv. Sci. 2022, 9, 2104089. 10.1002/advs.202104089 PMC9165482 35403829

[225] Y. Sun , W. Z. Lou , H. Z. Feng , W. T. Su , S. N. Lv , Microsys. Nanoeng. 2022, 8, 106. 10.1038/s41378-022-00441-8 PMC9508092 36164485

[226] S. Kianersi , A. Solouk , S. Saber‐Samandari , S. H. Keshel , P. Pasbakhsh , J Drug Deliv Sci Technol 2021, 66, 102889.

[227] A. Francioso , R. Cossi , S. Fanelli , P. Mastromarino , L. Mosca , Int. J. Mol. Sci. 2017, 18, 967. 28467361 10.3390/ijms18050967 PMC5454880

[228] F. Xie , L. Zou , Z. Xu , X. Ou , W. Guo , Y. Gao , G. Gao , Int. J. Biol. Macromol. 2022, 223, 391. 36356865 10.1016/j.ijbiomac.2022.11.013

[229] J. Liang , L. Li , H. Tian , Z. Wang , G. Liu , X. Duan , M. Guo , J. Liu , W. Zhang , E. C. Nice , C. Huang , W. He , H. Zhang , Q. Li , Small 2023, 19, 2303073. 10.1002/smll.202303073 37460404

[230] P. Wang , H. Liu , X. Pan , Q. Feng , J. Yang , J Drug Deliv Sci Technol 2022, 68, 103024.

[231] J. Ren , Y. Liu , Y. Yao , L. Feng , X. Zhao , Z. Li , L. Yang , Int. Immunopharmacol. 2021, 91, 107288. 33360827 10.1016/j.intimp.2020.107288

[232] L. Li , L. Tan , Q. Zhang , Y. Cheng , Y. Liu , R. Li , S. Hou , Front. Bioeng. Biotechnol. 2023, 11, 2023. 10.3389/fbioe.2023.1168408 PMC10084992 37051277

[233] A. S. Chowdhury , R. Geetha Bai , T. Islam , M. Abir , M. Narayan , Z. Khatun , Md. Nurunnabi , Biomater. Sci. 2022, 10, 2929. 35471198 10.1039/d2bm00316c PMC9949325

[234] Z. Cai , X. Lei , Z. Lin , J. Zhao , F. Wu , Z. Yang , J. Pu , Z. Liu , Acta Pharm. Sin. B 2014, 4, 86. 26579369 10.1016/j.apsb.2013.12.012 PMC4590720

[235] Z. Li , G. Zheng , N. Wang , H. Liang , C. Li , Y. Wang , Y. Cui , L. Yang , J. Agric. Food Chem. 2023, 71, 2883. 36722770 10.1021/acs.jafc.2c06809

[236] X. Zhou , X. Zhang , S. Han , Y. Dou , M. Liu , L. Zhang , J. Guo , Q. Shi , G. Gong , R. Wang , J. Hu , X. Li , J. Zhang , Nano Lett. 2017, 17, 1056. 28075596 10.1021/acs.nanolett.6b04523

[237] H. Pan , T. Sun , M. Cui , N. Ma , C. Yang , J. Liu , G. Pang , B. Liu , L. Li , X. Zhang , W. Zhang , J. Chang , H. Wang , ACS Nano 2022, 16, 6049. 35362965 10.1021/acsnano.1c11536

[238] R. Gupta , A. Chauhan , T. Kaur , B. K. Kuanr , D. Sharma , Nanoscale 2022, 14, 17589. 36409463 10.1039/d2nr02210a

[239] T. Lei , Z. Yang , C. Jiang , X. Wang , W. Yang , X. Yang , R. Xie , F. Tong , X. Xia , Q. Huang , Y. Du , Y. Huang , H. Gao , ACS Nano 2024, 18, 3234. 38214975 10.1021/acsnano.3c09715

[240] S. Kumar , J. Dutta , P. K. Dutta , J. Koh , Int. J. Biol. Macromol. 2020, 160, 470. 32464212 10.1016/j.ijbiomac.2020.05.192

[241] C. Wen , L. Cao , Z. Yu , G. Liu , J. Zhang , X. Xu , Crit. Rev. Food Sci. Nutr. 2023, 1. 10.1080/10408398.2023.2229433 37410019

[242] K. Wang , Q. Chen , L. Ding , Y. Zhu , X. Wang , M. Zhou , M. Chang , M. Pei , Y. Zhang , Y. Zhang , Y. Chen , H. Qin , Nano Today. 2023, 50, 101876.

[243] J. M. Carbonell‐Capella , M. Buniowska , F. J. Barba , M. J. Esteve , A. Frígola , Compr Rev Food Sci Food Saf 2014, 13, 155. 33412647 10.1111/1541-4337.12049

[244] D. Lin , A. L. Kelly , V. Maidannyk , S. Miao , Food Hydrocolloid. 2020, 108, 105998.

[245] Z. Jiao , X. Wang , Y. Yin , J. Xia , Y. Mei , J. Microencapsulation 2018, 35, 272. 29671362 10.1080/02652048.2018.1467509

