# Supplementary material for: Overcoming Biopotency Barriers: Advanced Oral Delivery Strategies for Enhancing the Efficacy of Bioactive Food Ingredients
Source: Adv Sci (Weinh). 2024 Oct 3;11(44):2401172. doi: 10.1002/advs.202401172 (PMC11600209; doi:10.1002/advs.202401172)
Supplement: Supplementary file 1 — Supporting Information [file ADVS-11-2401172-s001.docx]

**Supporting Information**

**Overcoming Biopotency Barriers: Advanced Oral Delivery Strategies for Enhancing the Efficacy of Bioactive Food Ingredients**

*Ling Liu*, *David Julian McClements*, *Xuebo Liu*, and *Fuguo Liu**

Table S1 The sources, physicochemical properties and physiological efficacies of main food bioactive ingredients.

| **Categories** | **Ingredients** | **Sources** | **Solubility** | **Stability** | **Bioactivities** | **Refs** |
| --- | --- | --- | --- | --- | --- | --- |
| ***Bioactive macromolecules*** | | | | | | |
| Polysaccharides | **Animal derived**:  Chitosan  Hyaluronic acids  Chondroitin sulfate  Glycogen | Liver, cartilage, crustacean | Water | Relatively stable | 1. Provide structural material and energy for human body  2. Antioxidant, antiviral, antitumor, anti-inflammation  3. Possess immunomodulatory activity, good biocompatibility, high stability, low toxicity, ease of modification  4. Protect skin, restore skin physiological function  5. Treat neurodegenerative diseases | [S1] |
|  | **Plant derived**:  Sodium alginate  Pectin  Acemannan  Starch  Cellulose  Cyclodextrin | Cereal, legumes, aloe, fruits, some terrestrial and marine plants | Water | Relatively stable | 1. Provide structural material and energy for plant cells  2. Antioxidant, antiviral, anti-inflammation  3. Possess neuroprotective immunomodulation, good biocompatibility, low toxicity  4. Chelate with metal ions  5. Promote wound healing | [S2] |
|  | **Microbial derived**:  Intracellular  Cell wall  Extracellular (dextran, xanthan gum) | Microorganisms | Water | Relatively stable | 1. Antioxidant, antiviral, anti-tumor, anti-obesity, anti-diabetic, anti-inflammation  2. Possess super-hydrophilicity, serum stability, good biomimetic properties and biodegradability  3. Modulate immune system, avoid rapid recognition and removal by immune system  4. Protect liver and kidney  5. Regulate cell division, growth and aging | [S3] |
| Proteins | **Animal derived**:  Globulin  Ferritin  Transferrin  Albumin  Whey  Casein  Collagen  Gelatin | Milk, meat, human/bovine serum, eggs | Water and lipid | Unstable:  heat, ions, pH changes | 1. High nutritional quality, decomposed as bioactive peptides or essential amino acids  2. Antimicrobial, antiviral, anti-fungal, anticancer, anti-lipidemic, antioxidant, anti-inflammation  3. Possess mineral-binding capacity  4. Treat iron deficiency anemia and magnesium deficiency neuritis  5. Prevent caries, osteoporosis and rickets  6. Modulate blood pressure and immune system  7. Produce satiety  8. Protect skin | [S4]  [S5] |
|  | **Plant derived**:  Zein  Gluten  Gliadin  Soy/pea isolated proteins | Wheat, maize, legumes | Water and lipid | Unstable:  heat, ions, pH changes | 1. Antioxidant, antimicrobial, anti-hypertension, anti-tumor, anti-inflammation, anti-diabetic  2. Possess good biocompatibility, biodegradability, self-assembly and environmental-friendly characteristics, film formation, amphiphilicity  3. Possess mucus adhesion and controlled release capacities  4. Induce new tissues formation, reduce inflammatory response of host cells, promote wound healing | [S6] |
|  | **Microbial derived**:  Lactococcal protein | Mushroom, yeast, molds, bacteria | Water and lipid | Unstable:  heat, ions, pH changes | 1. Antibacterial, antifungal, antiviral, anti-cancer  2. Enhance immune function | [S7] |
| Lipids | **Unsaturated fatty acids**:  Oleic acids  Linoleic acids  α-linolenic acids  Arachidonic acids  Docosahexaenoic acids  Eicosapentaenoic acids  Docosapentaenoic acids | Seeds, fish oil, yogurt, some vegetables and fruits | Lipid | Unstable:  heat, light, oxygen | 1. Maintain the relative fluidity of cell membranes to ensure normal physiological functions of cells  2. Lower blood cholesterol and triglycerides  3. Promote saturated fatty acids metabolism, reduce blood viscosity, improve blood microcirculation  4. Control blood lipids concentration,  prevent platelet adhesion and cohesion, prevent thrombosis and stroke  5. Modulate immune system  6. Improve brain cells activity, promote the full development of brain cells, enhance memory and thinking ability  7. Protect retina and improve eyesight  8. Improve arthritis symptoms to reduce pain | [S8] |
|  | **Phospholipids**:  Yolk phospholipids  Krill oil phospholipids  Soybean phospholipids  Perilla seed phospholipids  Phosphatidylserine | Human body;  Egg yolks, milk, brain, liver, kidney, muscle, seeds of oil species | Water and lipid | Relatively stable | 1. Possess emulsifying ability, decompose excessive blood lipids and cholesterol, lower serum cholesterol, relieve wall pressure on the heart and brain vessels, improve blood circulation and prevent cardiovascular diseases  2. Maintain basal metabolism and balance hormonal secretion  3. Maintain the physiological vitality of normal cells  4. Enhance immunity and regeneration of human body  5. Accelerate the information transmission between nerve cells and brain cells, enhance memory and prevent dementia | [S9] |
|  | **Sterols**:  Cholesterol  Phytosterol (β-Sitosterol)  Ergosterol | Cell membrane of animals, plants and fungus | Lipid | Relatively stable | 1. Involved in blood lipids transport  2. Prevent cardiovascular diseases, inhibit cholesterol synthesis in liver  3. Anti-tumor (colorectal cancer, skin cancer, cervical cancer)  4. Promote metabolism  5. Promote renal tubular reabsorption  6. Maintain secondary sexual characteristics  7. Promote calcium and phosphorus absorption  8. Regulate hormone levels | [S10] |
| ***Bioactive small molecules*** | | | | | | |
| Peptides | **Natural endogenous**:  Peptide hormones  Peptide enzyme activators  Kinins (bradykinin, angiotensin) | Human body | Water and lipid | Relatively stable | 1. Regulate water and electrolyte balance in human body  2. Antibacterial, anti-infection, anti-cancer, anti-inflammation, anti-diabetic  3. Antioxidant, anti-aging, eliminate excess free radicals in human body  4. Anti-hypertension, anti-thrombosis, anti-high cholesterol, prevent cardiovascular and cerebrovascular diseases  5. Modulate immune activity  6. Promote wound healing  7. Treat osteoporosis  8. Repair cells and improve cell metabolism  9. Regulate endocrine and nervous system  10. Improve digestive system and treat chronic gastrointestinal disorders  11. Improve diabetes, rheumatism and rheumatoid diseases  12. Promote hematopoietic function, prevent platelet aggregation, improve the oxygen-carrying capacity of red blood cells  13. Antiviral, directly against DNA viruses, targeting viral bacteria  14. Bind minerals, promote mineral absorption | [S11] |
|  | **Natural exogenous**:  Casein phosphopeptides  Carnosine  Collagen peptides  Wheat oligopeptides  Glutathione  Nisin | Animals (milk, cheese, meat, egg); Plants (legumes, cereal, seeds); Microorganisms (mushrooms, ganoderma lucidum, agrocybe cylindracea, some probiotics, yeast) | Water and lipid | Relatively stable |  |  |
|  | **Artificial synthetic**:  Chemosynthetic peptides  Recombinant peptides | Biosynthesis | Water and lipid | Relatively stable |  |  |
| Polyphenols | **Flavonoids**:  Quercetin  EGCG  Anthocyanins | Vegetables, fruits, tea leaves, herbs, wine, olive oil, honey, cereals, legumes | Water or lipid | Unstable:  heat, light, oxygen | 1. Antioxidant, antimicrobial, anti-virus, anti-tumor, anti-inflammation, antalgic  2. Inhibit apoptosis and protect liver  3. Prevent cardiovascular diseases  4. Modulate animal hormone levels  5. Improve immune system function | [S12] |
|  | **Phenolic acids**:  Chlorogenic acids  Caffeic acids  Cinnamic acids | Berries, spices, cereals, tea leaves, | Water or lipid | Unstable:  heat, light, oxygen | 1. Antioxidant, antineoplastic, bacteriostatic, anti-inflammation, anti-melanogenic, antirheumatic, antimutation, analgesic, antipyretic  2. Anti-cancer (prostate cancer, colon cancer, cervical cancer)  3. Inhibit apoptosis and protect liver  4. Possess anthelmintic activity  5. Possess neuroprotective activity  6. Suppress hepatic fibrosis in chronic liver injury  7. Possess convergence and hemostatic functions | [S13, S14] |
|  | **Lignans**:  Sesamin  Syringaresinol  Medioresinol | Whole bran cereals, sesames, flaxseeds | Lipid | Unstable:  heat, light, oxygen | 1. Antioxidant, antimicrobial, antiviral, anti-carcinogenic, anti-tumor  2. Anti-inflammation, inhibit NF-κB activity on human mast cells, reduced pro-inflammatory cytokines production, suppress nitric oxide generation and decrease inflammatory cell infiltration  3. Anti-estrogenic, ameliorate menopausal symptoms and consequences of post-menopausal women | [S15] |
|  | **Stilbenes**:  Resveratrol  Piceatannol  Pinostilbene | Wine, grapes, peanuts, sorghum, some tree species | Lipid | Unstable:  heat, light, oxygen | 1. Antioxidant, antibacterial, anti-allergic, anti-tumor, anti-hypertensive  2. Prevent degenerative diseases (cancer, cardiovascular diseases, neurodegenerative diseases)  3. Possess hypolipidemic and hepatoprotective effects  4. Inhibit platelet aggregation  5. Improve the microcirculation of capillary and coronary vessels | [S16] |
|  | Tyrosol | Olive oil, tea leaves, wine | Lipid | Unstable:  heat, oxygen | 1. Antioxidant, antibacterial, anti-cancer, anti-inflammation  2. Prevent cardiovascular diseases  3. Maintain normal metabolism  4. Protect nervous system, prevent neurodegenerative diseases | [S17] |
| Terpenoids | **Carotenoids**:  Carotenes (α、β、γ)  Lycopene  Capsaicin  Astaxanthin  Lutein | Carrots, tomatoes, watermelons, chili, etc. | Lipid | Unstable:  heat, light, oxygen, pH changes, moisture | 1. Antioxidant, antimicrobial, anti-cancer, antineoplastic, anti-allergic, anti-inflammation, antipruritic, antipyretic, analgesic  2. Subside swelling, promote blood circulation to dissipate blood stasis  3. Regulate blood sugar and blood lipid  4. Possess parasite expelling ability  5. Prevent phlegm forming and stop coughing  6. Regulate immune system  7. Retard aging process  8. Invigorate stomach  9. Local anesthetic  10. Reduce drug toxicity  11. Possess strong cell penetration ability  12. Neuroprotective capability, block the function of nicotinic acetylcholine receptors | [S14]  [S18] |
|  | **Flavors**:  Limonene  Menthone  Geraniol | Lemon, orange, mint, camphor, pine, etc. | Lipid | Unstable (volatile):  heat, light, oxygen, moisture |  | [S14]  [S18] |
| Vitamins | VA_1_/Retinol  VA_2_/ 3-Dehydroretinol | Animal origin (Vitamin A): liver, eggs, fish, milk and dairy products etc.  Plant origin (Provitamin A): reddish, yellow and dark green fruits and vegetables, *e.g.,* carrots, pumpkin, spinach | Lipid | Stable:  heat, base  Unstable  light, oxygen, acid, metal ions | 1. Visual function  2. Maintain skin mucosal integrity  3. Hormone-like effects on the cell nucleus  4. Promote immune function  5. Promote growth and reproductive function  6. Antagonism to VD and promote bone metabolism  7. Anti-cellular proliferative  8. Promotes hemoglobin production and increases dietary iron intake | [S19] |
|  | VD_2_/Ergocalciferol  VD_3_/Cholecalciferol | Liver, some species of fish, eggs, cream, some species of mushroom | Lipid | Stable:  heat, base, oxygen  Unstable:  acid, light | 1. Regulate calcium and phosphorus metabolism  2. Promote bone growth  3. Regulate cell growth and differentiation  4. Regulate immune function | [S20] |
|  | VE/Tocopherols | Several fruits, vegetables, nuts, cereals, seeds, seed oils | Lipid | Stable:  heat, acid  Unstable:  oxygen, light, base, metal ions | 1. Anti-oxidation  2. Delays body aging  3. Maintain normal reproductive function  4. Stimulate immune system  5. Maintain tissue elasticity  6. Protect skin mucous membranes  7. Avoid coronary arteriosclerosis  8. Delay premature senile dementia | [S21] |
|  | VK_1_/Phylloquinone  VK_2_/Menaquinones | Leafy green vegetables, liver, egg yolks | Lipid | Stable:  heat, acid  Unstable:  light, base | 1. Regulate blood coagulation  2. Prevent vascular calcification  3. Involved in bone metabolism  4. Involved in γ-carboxyglutamate synthesis  5. Modulate cell proliferation | [S22] |
|  | VC/Ascorbic acid | Several fruits, liver, many vegetables | Water | Stable:  acid  Unstable:  base, heat, light, oxygen, metal ions | 1. Anti-oxidation  2. Boost immunity  3. Anti-ageing effect  4. Enhance immunity  5. Promote skin whitening  6. Treat iron deficiency anemia  7. Reduce the incidence of stomach and esophageal cancers | [S23] |
|  | VB_1_/Thiamin | Cereals, legumes, nuts, dried yeast, animal offal (liver, kidneys, lean meat, egg yolk), some vegetables (celery, purple cabbage) | Water | Stable:  acid  Unstable:  base, heat, oxygen | 1. Promote growth and digestion  2. Improve mental state  3. Maintain normal nerve, muscle and heart activities  4. Improve seasickness and motion sickness reactions | [S24] |
|  | VB_2_/Riboflavin | Liver, fish, eggs, milk and dairy products, several vegetables, several fruits | Water | Stable:  heat, acid, oxygen  Unstable:  base, light | 1. Anti-oxidation  2. Involved in energy metabolism  3. Promote growth and development  4. Maintain the integrity of the skin and cell membranes  5. Associated with the absorption and storage of iron |  |
|  | VB_3_/VPP/Niacin or Nicotinic acid | Liver, meat, eggs yolk, fish, milk, several vegetables, fruits, tea | Water | Stable:  heat, base, light, oxygen | 1. Promote normal body growth and development  2. Promote iron absorption and blood cell production, influencing the hematopoietic process  3. Maintain normal skin function and secretion of digestive glands  4. Increase central nervous excitability  5. Treat various skin diseases, hypertension and coronary heart diseases |  |
|  | VB_4_/Adenine | Animal offal, meat, soy products, shrimp, sardines, black fungus, squid, mushrooms | Water | Relatively stable | 1. Regulate heart rate  2. Relieve fatigue  3. Strengthen immune function  4. Prevent the free radical formation  5. Participate in the regulation of blood sugar balance |  |
|  | VB_5_/Pantothenic acid | Meat, unrefined cereals, animal offal, green vegetables | Water | Stable:  acid, oxygen  Unstable:  heat, base, light | 1. Involved in energy metabolic reactions  2. Involved in melatonin and ferrous hemoglobin synthesis  3. Improve pathogen resistance  4. Reduce apoptosis and cell damage |  |
|  | VB_6_/Pyridoxine | Yeast, liver, grains, meat, fish, eggs, legumes, peanuts, several vegetables | Water | Stable:  acid, light  Unstable:  heat, base | 1. Involved in protein synthesis and catabolism  2. Involved in the synthesis of certain neurotransmitters (5-hydroxytryptamine, taurine, dopamine, norepinephrine and γ-aminobutyric acid)  3. Maintain immune function  4. Reduce the incidence of chronic and cardiovascular diseases |  |
|  | VB_7_/VH/Biotin/coenzyme R | Liver, meat, whole grain products (especially wheat), vegetables and nuts, yeast, egg yolk | Water | Stable:  acid, base, light  Unstable:  heat, oxygen | 1. Enhance the immune response and immunity  2. Maintain body growth and development |  |
|  | VB_8_/Inositol | Yeast, liver, whole grains, cantaloupe, citrus fruits (except lemon), nuts, legumes, cabbage, intestinal bacteria | Lipid | Stable:  heat, oxygen, pH changes | 1. As a lipotropic agent, accelerate the breakdown of fats, prevent abnormal or excessive accumulation of fats, and increase the utilization of fats  2. Facilitate neural signal transduction  3. Control intracellular Ca^2+^ concentration  4. Treat psychological disorders such as depression, bipolar disorder and panic disorder |  |
|  | VB_9_/VM/VBc/VB_11_/Folic acid | Leafy vegetables, liver, kidney, egg yolk, fruits (citrus, kiwi), yeast | Water | Stable:  base  Unstable:  heat, acid, light, oxygen, metal ions | 1. Participate in genetic and proteins metabolism  2. Influence animal reproductive  3. Improve organism immunity |  |
|  | VB_10_/VR | a mixture of VB_9_ and other B vitamins |  |  |  |  |
|  | VB_12_/Cobalamin | Meat, fish, poultry, eggs, milk and dairy products | Water | Stable:  Weak acid  Unstable:  Strong acid, base, light, heat | 1. Promote proteins biosynthesis  2. Protect the growth and development of infants and children  3. Protect intracellular transfer and storage of folic acids |  |
| Minerals | Micro elements (Ca, Mg, K, etc.)  Trace elements (Fe, Zn, Si, etc.) | Widely distributed in nature (soil, air, water, foods, etc.) | Water | Relatively Stable | 1. Strengthen bones  2. Transmitting nerve impulse  3. Regulate the level of hormones and glucose  4. Control blood pressure  5. Involved in immune and brain systems  6. Maintain normal growth and development of body | [S25] |
| Alkaloids | Diterpenoid alkaloids  Steroidal alkaloids  Purine alkaloids  Pyridine alkaloids | Plants (dicotyledonous plants), some animals | Lipid | Weak stability | 1. Anti-inflammation, analgesia, anti-hepatic fibrosis, immunosuppression  2. Anti-tumor by inhibiting the formation of microtubule of tumor cell’s mitotic spindle, and thus inhibiting proliferation and inducing cytotoxicity to tumor cells  3. Treat cerebrovascular and cardiovascular diseases and rheumatic diseases | [S26] |
| Essential oils | Terpenes & terpenoids  Aromatic & aliphatic compounds | Spiced plants, perfumed animals | Lipid | Unstable:  heat, light, oxygen | Antibacterial, antioxidant, anti-inflammation, anti-cancer, insecticidal efficacy | [S27] |
| Saponins | Triterpenoid saponins  Steroidal saponins | Plants (Triterpenoid saponins come from legumes, tea leaves, etc; Steroidal saponins come from oats, ginseng roots, etc.)  Some marine animals (starfishes, sea cucumber) | Water | Relatively stable | 1. Antibacterial, antipyretic, sedative, anticancer, antivirus, anti-obesity  2. Hypocholesterolemia action  3. Inhibit digestive enzymes  4. Influence gut microbiota  5. Modulate immune system  6. Prevent cardiovascular diseases  7. Protect liver | [S28] |
| ***Probiotics*** | | | | | | |
| *Lactic acid bacteria* | *Lactobacillus acidophilus*  *Pedicoccus acidilactici*  *Streptococcus lactis* | Widely distributed in nature (soil, air, water, foods, etc.) and intestinal tract | - | Unstable:  pH changes, heat, salt | 1. Antifungal, antioxidant, anti-cancer, antiviral, anticoagulant  2. Enhance immunity  3. Improve gastrointestinal function  4. Increase resistance to obesity  5. Reduce blood glucose concentration and cholesterol | [S29] |
| *Lactobacillus* | *Lactobacillus plantarum*  *Lactobacillus bulgaricus*  *Lactobacillus rhamnosus* | Widely distributed in nature (soil, air, water, foods, etc.) and intestinal tract | - | Unstable:  pH changes, heat, salt | 1. Prevent some inflammatory diseases (rheumatoid arthritis) and allergic disorders  2. Decrease the incidence of diarrhea, lactose intolerance, irritable bowel syndrome  3. Protect against colon and bladder cancer  4. Treat helicobacter pylori infection  5. Improve mental/neurological illness (Alzheimer, Parkinson)  6. Improve obesity, diabetes | [S30] |
| *Bifidobacterium* | *Bifidobacterium adolescentis*  *Bifidobacterium breve*  *Bifidobacterium longum* | Widely distributed in nature (soil, air, water, foods, etc.), oral cavity and intestinal tract | - | Unstable:  pH changes, heat, salt |  |  |
| *Coccus* | *Enterococcus faecalis*  *Streptococcus thermophilus*  *Pediococcus pentosaceus* | Widely distributed in nature (soil, air, water, foods, etc.) and intestinal tract | - | Unstable:  pH changes, heat, salt |  |  |
| *Bacillus* | *Bacillus subtilis*  *Bacillus licheniformis*  *Bacillus coagulans* | Widely distributed in nature (soil, air, water, foods, etc.) and intestinal tract | - | Unstable:  pH changes, heat, salt |  |  |
| *Saccharomyces* | *Saccharomyces cerevisiae*  *Saccharomyces boulardii*  *Rhodotorula mucilaginosa* | Widely distributed in nature (soil, air, water, foods, etc.) | - | Unstable:  pH changes, heat, salt | 1. Antibacterial, antiviral, anti-carcinogenic, antioxidant, anti-inflammation, immune-modulation  2. Protect normal microbiota of human gut  3. Inhibit the pathogenicity of different diarrheal infections  4. Treat multiple gastrointestinal diseases | [S31] |
| Engineered probiotics | SYNB8802  i-ROBOT  EcNL4 | *Escherichia coli* Nissle 1917  *Lactobacillus reuteri*  *Lactobacillus casei*  *Lactococcus lactis* | - | Unstable:  pH changes, heat, salt | Treat chronic diseases (cancer, arthritis, inflammatory bowel disease, diabetes, cardiovascular disease, neurodegenerative disease, intestinal hyperoxaluria, phenylketonuria and chronic nephrolithiasis) | [S32]  [S33]  [S34]  [S35] |

Table S2 Main strategies and common delivery systems for overcoming in vitro barriers of food bioactive ingredients.

| **Targeted barriers** | **Type of strategies** | **Preparation methods** | **Wall materials** | **Core materials** | **Refs** |
| --- | --- | --- | --- | --- | --- |
| ***Inherent defects*** | | | | | |
| *Low solubility | O/W emulsions | High pressure homogenization | External phase: water  Emulsifier: CSC  Internal phase: MCT | Astaxanthin | [S36] |
|  | Cavity carriers | Freeze drying method | β-cyclodextrin | Cannabidiol | [S37] |
|  | Nanoparticles | Film casting method  Self-assembly method | HSA-PEG | Paclitaxel | [S38] |
|  | Dendrimers | PEG -OH modification  EDC/NHS conjugation | G4 PAMAM dendrimers-PEG | Piperlongumine | [S39] |
|  | Hydrogels | Single-step solid dispersion  High-pressure homogenization  Free radical polymerization  Swelling diffusion method | Pullulan-MAA | Crude curcumin  Curcumin nanocrystal  Curcumin micelle | [S40] |
|  | Micelles | OSA modification  Self-assembly method | Oat β-glucan-OSA | β-carotene | [S41] |
| *Low permeability | W/O emulsions | High shear homogenization | External phase:  corn oil/CBR/stearic acid  Emulsifier: PGPR  Internal phase:  water/LMP/GDL/CaCO_3_ | *Bifidobacterium lactis V9* | [S42] |
|  | Liposomes | Thin-lipid film hydration | L-α-phosphatidylcholine  β-sitosterol | Resveratrol | [S43] |
|  | Liquid crystals | Composition-tuning method  Self-assembly method | Phase 1: MO  Stabilizer: TPGS-PEG_1000_  Phase 2: water | Fish oil | [S44] |
|  | Organic nanotubes | Ion concentration adjustment  Self-assembly method | α-Lactalbumin | Capsaicin | [S45] |
|  | Inorganic nanoparticles | Co-precipitation method  Self-assembly method | Fe_3_O_4_-CLA | Paclitaxel | [S46] |
|  | Exosomes | Adsorption and Coating | Ginger-derived exosome  Large mesoporous silicon | Infliximab | [S47] |
|  | Cells | Separation and Coating | Red blood cells-RGD | Doxorubicin | [S48] |
|  | Virus-mimicking nanoparticles | Self-polymerization  Coating | BCA-FA(-)-CS(+)-HA | Insulin | [S49] |
|  | Microneedles | 3D stacking strategy | Tip: GelMA  Connection: GelMA-BSA  Substrate: PEGDA-NdFeB | Insulin | [S50] |
|  | Modified liposomes | Thin-film hydration | Phospholipid: soy lecithin  Stabilizer: tween 80  Modification: inulin | Cinnamaldehyde | [S51] |
|  | Modified SLNs | Homogenization-sonication | Phospholipid: lecithin  Solid lipid: dynasan 116  Stabilizer: tween 80  Modification: ApoE | Donepezil | [S52] |
|  | Modified NLCs | Emulsification method  Solvent evaporation method | Phospholipid: soy lecithin  Liquid lipid: ATO888, MCT812  Modification: cFA, dNP2 | Paclitaxel | [S53] |
| ***Negative interactions*** | | | | | |
| *Synergistic inhibition | Double emulsions | High shear homogenization | External phase:  water, CaCl_2_  Stabilizer: bacterial cellulose  Internal phase:  soy oil, PGPR | EGCG  Lycopene | [S54] |
|  | Hydrogels | Ethanol induction method | Propylene glycol alginate  β-lactoglobulin | *Lactobacillus rhamnosus*  Curcumin | [S55] |
|  | Liposomes | Thin-layer dispersion method  Microfluidization method | Phospholipid:  Soy L-α-Phosphatidylcholine | DHA  Anthocyanidin | [S56] |
|  | SLNs | Hot melt ultrasonication | Phospholipid: lecithin  Solid lipid: witepsol, carnauba  Stabilizer: tween 80 | Rosmarinic acid  Herbal extracts | [S57] |
|  | NLCs | Hot homogenization method | Phospholipid: lecithin  Liquid lipid: ATO, MCT812  Surfactant: poloxamer 470 | Erlotinib  Resveratrol | [S58] |
|  | Nanocages | Reversible assembly method | Red bean seeds deprived ferritin | EGCG  Quercetin | [S59] |
| ***Environmental limits*** | | | | | |
| *Light | Cavity complexes | Solvothermal method  Ultrasonic method | γ-cyclodextrin  MOF | Curcumin | [S60] |
| *Oxygen | Cells | Separation | Yeast cell | Antarctic krill oil | [S61] |
| *Moisture | Organic-inorganic complexes | Solution soaking method  Spray mixing method | Wheat dietary fiber  Soy protein | Iodine salts | [S62] |
| ***Processing limits*** | | | | | |
| *Physical processing |  |  |  |  |  |
| Heating | Nanobeads | External gelation method | Alginate  Starch | Iron | [S63] |
| Freezing | Nanoparticles | Nanoprecipitation method | Zein  Cryoprotectant: sucrose | Resveratrol | [S64] |
| Irradiation | Hydrogels | Electron beam irradiation  Sorption method | Carboxymethyl chitosan  Poly(vinylpyrrolidone) | Kanamycin | [S65] |
| Microwave | Emulsions | Microwave heating method | Phase 1: peanut oil  Stabilizer: WPI nanofibrils  Phase 2: water | *D*-limonene | [S66] |
| High pressure | Emulsions | High pressure homogenization | Phase 1:  Sichuan pepper essential oil  Emulsifier: Tween 80, ODO  Phase 2: water | Sichuan pepper essential oil | [S67] |
| Sonication | Liposomes | Homogenization  Sonication | Phospholipid: soy lecithin | High oleic palm oil | [S68] |
| Pulsed electric field | Nanocages | Pulsed electric field method | Horse spleen shell-like ferritin | - | [S69] |
| *Chemical processing |  |  |  |  |  |
| pH | Nanoparticles | Anti-solvent precipitation | Zein  Mushroom β-glucans | - | [S70] |
| Ion | Nanoparticles | Self-assembly method | Oleanolic acid | β-carotene | [S71] |
| Enzyme | Nanocomposites | Maillard glycosylation method  Self-assembly method | Arachin, Casein | EGCG | [S72] |
| *Biological processing |  |  |  |  |  |
| Fermentation | Foodstuffs | Solid-state fermentation | Whole-grain oats | Lactic acid bacteria | [S73] |

Notes:

CSC: casein-caffeic acid-glucose ternary conjugate; MCT: medium chain triglyceride; HSA: human serum albumin; PEG: poly (ethylene) glycol; -OH: hydroxyl group; EDC: 1-(3-Dimethylaminopropyl)-3-ethylcarbodiimide hydrochloride; NHS: N-hydroxy succinimide; G4 PAMAM: generation 4 poly (amidoamine); MAA: Methacrylic acid; OSA: octenylsuccinated; CBR: cocoa butter replacer; PGPR: polyglycerol polyricinoleate; LMP: low methoxy pectin; GDL: D-(+)-gluconic acid δ-lactone; MO: glycerol monooleate; TPGS-PEG_1000_: PEGylated D-α-tocopheryl-poly(ethylene glycol)_1000_; CLA: *trans-10*, *cis-12* conjugated linoleic acid; RGD: arginine-glycine-aspartic acid; BCA: butylcyanoacrylate; FA: folic acid; CS: chitosan; HA: hyaluronic acid; GelMA: Gelatin methacryloyl; BSA: bovine serum albumin; PEGDA: poly(ethylene glycol) diacrylate; NdFeB: magnetizable microparticles; DSPE: distearoyl phosphoethanolamine; PEG: polyethylene glycol; CD33L: glycan-binding protein ligand; ApoE: Apolipoprotein E; ATO: monobehenin; cFA: acid-cleavable folic acid; dNP2: human-derived cell penetrating peptide; EGCG: epigallocatechin gallate; DHA: docosahexaenoic acid; MOF: metal organic framework; WPI: whey protein isolate; ODO: a non-polar medium carbon chain fatty acid glyceride.

**References to the supplementary tables**

1. Muxika, A., Etxabide, J., Uranga, P., et al., 2017. Chitosan as a bioactive polymer: Processing, properties and applications. *International Journal of Biological Macromolecules*. 105(2), 1358-1368. <https://doi.org/10.1016/j.ijbiomac.2017.07.087>.
2. Xu, X. L., Li, S., Zhang, R., et al., 2022. Neuroprotective effects of naturally sourced bioactive polysaccharides: an update. *Neural Regeneration Research*. 17(9), 1907-1912. <https://doi.org/10.4103/1673-5374.335142>
3. Yildiz, H., Karatas, N., 2018. Microbial exopolysaccharides: Resources and bioactive properties. *Process Biochemistry*. 72, 41-46. <https://doi.org/10.1016/j.procbio.2018.06.009>.
4. Auestad, N., Layman, D. K., 2021. Dairy bioactive proteins and peptides: a narrative review. *Nutrition Reviews*. 79(2), 36-47. <https://doi.org/10.1093/nutrit/nuab09>
5. Chen, H., Tan, X. Y., Han, X. E., et al., 2022. Ferritin nanocage based delivery vehicles: From single-, co- to compartmentalized- encapsulation of bioactive or nutraceutical compounds. *Biotechnology Advances*. 61, 108037. <https://doi.org/10.1016/j.biotechadv.2022.108037>
6. Guan, T. W., Zhang, Z. H., Li, X. J., et al., 2022. Preparation, characteristics, and advantages of plant protein-based bioactive molecule delivery systems. *Foods*. 11(11), 1562. <https://doi.org/10.3390/foods11111562>
7. Ng, T. B., Cheung, R. C. F., Wong, J. H. et al., 2016. Fungal proteinaceous compounds with multiple biological activities. *Applied Microbiology and Biotechnology*. 100, 6601-6617. <https://doi.org/10.1007/s00253-016-7671-9>
8. Angelo, S. D., Motti, M. L., Meccariello, R., 2020. ω-3 and ω-6 polyunsaturated fatty acids, obesity and cancer. *Nutrients.* 12(9), 2751. <https://doi.org/10.3390/nu12092751>
9. Sun, N., Chen, J., Wang, D., 2018. Advance in food-derived phospholipids: Sources, molecular species and structure as well as their biological activities. *Trends in Food Science & Technology.* 80, 199-211. <https://doi.org/10.1016/j.tifs.2018.08.010>
10. Zhang, K., Li, T. Z., Shan, X. J., 2021. Cholesterol: Bioactivities, structural modification, mechanisms of action, and structure-activity relationships. *Mini-Reviews in Medicinal Chemistry.* 21(14), 1830-1848. <https://doi.org/10.2174/1389557521666210105123320>
11. Jia, L. T., Wang, L., Liu, C., 2021. Bioactive peptides from foods: production, function, and application. *Food & Function*. 12(16), 7108-7125. <https://doi.org/10.1039/D1FO01265G>
12. Hostetler, G. L., Ralston, R. A., Schwartz, S. J., 2017. Flavones: food sources, bioavailability, metabolism, and bioactivity. *Advances in Nutrition*. 8(3), 423-435. <https://doi.org/10.3945/an.116.012948>.
13. Heleno, S. A., Martins, A., Queiroz, M. J. R. P., et al., 2015. Bioactivity of phenolic acids: metabolites versus parent compounds: a review. *Food Chemistry.* 173, 501-513. <https://doi.org/10.1016/j.foodchem.2014.10.057>.
14. Fraga, C. G., Croft, K. D., Kennedy, D. O., et al., 2019. The effects of polyphenols and other bioactives on human health. *Food & Function.* 10, 514. <https://doi.org/10.1039/c8fo01997e>
15. Rodríguez-García, C., Sánchez-Quesada, C., Toledo, E., et al., 2019. Naturally lignan-rich foods: A dietary tool for health promotion? *Molecules*. 24(5), 917. <https://doi.org/10.3390/molecules24050917>
16. Jarosova, V., Vesely, O., Marsik, P., et al., 2019. Metabolism of stilbenoids by human faecal microbiota. *Molecules.* 24(6), 1155. <https://doi.org/10.3390/molecules24061155>
17. Rodríguez-Morató, J., Boronat, A., Kotronoulas, A., et al., 2016. Metabolic disposition and biological significance of simple phenols of dietary origin: hydroxytyrosol and tyrosol. *Drug Metabolism Reviews.* 48(2), 218-236. <https://doi.org/10.1080/03602532.2016.1179754>
18. Soto, E. R., Rus, F., Li, H. C., et al., 2021. Yeast particle encapsulation of scaffolded terpene compounds for controlled terpene release. *Foods.* 10(6), 1207. <https://doi.org/10.3390/foods10061207>
19. Maurya, V. K., Shakya, A., Bashir, K., et al., 2022. Vitamin A fortification: Recent advances in encapsulation technologies. *Comprehensive Reviews in Food Science and Food Safety.* 21(3), 2075-3029. <https://doi.org/10.1111/1541-4337.12941>
20. Kutner, A., Brown, G., 2018. Vitamins D: Relationship between structure and biological activity. *International Journal of Molecular Sciences.* 19(7), 2119. <https://doi.org/10.3390/ijms19072119>
21. Brigelius-Flohé, R., 2021. Vitamin E research: Past, now and future. *Free Radical Biology and Medicine.* 177, 381-390. <https://doi.org/10.1016/j.freeradbiomed.2021.10.029>.
22. Bus, K., Szterk, A., 2021. Relationship between structure and biological activity of various Vitamin K forms. *Foods.* 10(12), 3136. <https://doi.org/10.3390/foods10123136>
23. Macan, A. M., Kraljević, T. G., Raić-Malić, S., 2019. Therapeutic perspective of Vitamin C and its derivatives. *Antioxidants.* 8(8), 247. <https://doi.org/10.3390/antiox8080247>
24. Uebanso, T., Shimohata, T., Mawatari, K., et al., 2020. Functional roles of B-Vitamins in the gut and gut microbiome. *Molecular Nutrition & Food Research.* (64)18, 2000426. <https://doi.org/10.1002/mnfr.202000426>
25. Gharibzahedi, S. M. T., Jafari, S. M., 2017. The importance of minerals in human nutrition: Bioavailability, food fortification, processing effects and nanoencapsulation. *Trends in Food Science & Technology.* 62, 119-132. <https://doi.org/10.1016/j.tifs.2017.02.017>.
26. Zheng, X., Wu, F., Lin, X., et al., 2018. Developments in drug delivery of bioactive alkaloids derived from traditional Chinese medicine. *Drug Delivery.* 25(1), 398-416. <https://doi.org/10.1080/10717544.2018.1431980>
27. Sharifi-Rad, J., Sureda, A., Tenore, G. C., et al., 2017. Biological activities of essential oils: From plant chemoecology to traditional healing systems. *Molecules.* 22(1), 70. <https://doi.org/10.3390/molecules22010070>
28. Navarro del Hierro, J., Herrera, T., Fornari, T., et al., 2018. The gastrointestinal behavior of saponins and its significance for their bioavailability and bioactivities. *Journal of Functional Foods.* 40, 484-497. <https://doi.org/10.1016/j.jff.2017.11.032>.
29. Liu, C. R., Zheng, J. Q., Ou, X., et al., 2021. Anti-cancer substances and safety of lactic acid bacteria in clinical treatment. *Frontiers in Microbiology.* 12: 722052. <https://doi.org/10.3389/fmicb.2021.722052>
30. Reque, P. M., Brandelli, A., 2021. Encapsulation of probiotics and nutraceuticals: Applications in functional food industry. *Trends in Food Science & Technology.* 114, 1-10. <https://doi.org/10.1016/j.tifs.2021.05.022>
31. Abid, R., Waseem, H., Ali, J., et al., 2022. Probiotic yeast saccharomyces: Back to nature to improve human health. *Journal of Fungi*. 8(5), 444. <https://doi.org/10.3390/jof8050444>
32. Lubkowicz, D., Horvath, N. G., James, M. J., et al., 2022. An engineered bacterial therapeutic lowers urinary oxalate in preclinical models and in silico simulations of enteric hyperoxaluria. *Molecular Systems Biology.* 18: e10539. <https://doi.org/10.15252/msb.202110539>
33. Zou, Z. P., Du, Y., Fang, T. T., et al., 2023. Biomarker-responsive engineered probiotic diagnoses, records, and ameliorates inflammatory bowel disease in mice. *Cell Host & Microbe.* 31(2), 199-212.e5. <https://doi.org/10.1016/j.chom.2022.12.004>.
34. Yan, X., Liu, X. Y., Zhang, D., et al., 2021. Construction of a sustainable 3-hydroxybutyrate-producing probiotic Escherichia coli for treatment of colitis. *Cellular & Molecular Immunology.* 18, 2344-2357. <https://doi.org/10.1038/s41423-021-00760-2>
35. Barati, M., Jabbari, M., Ghavidel, A. A., et al., 2022. The engineered probiotics for the treatment of chronic diseases: A systematic review. *Journal of Food Biochemistry.* 46, e14343. <https://doi.org/10.1111/jfbc.14343>
36. Cheng, J. R., Shen, S. W., Yang, H. G., et al., 2023. Improved physicochemical stability and bioaccessibility of astaxanthin-loaded oil-in-water emulsions by a casein-caffeic acid-glucose ternary conjugate. *Food Research International.* 163, 112153. <https://doi.org/10.1016/j.foodres.2022.112153>
37. Li, H., Chang, S. L., Chang T. R., et al., 2021. Inclusion complexes of cannabidiol with β-cyclodextrin and its derivative: Physicochemical properties, water solubility, and antioxidant activity. *Journal of Molecular Liquids.* 334, 116070. <https://doi.org/10.1016/j.molliq.2021.116070>
38. Lee, J. E., Kim, M. G., Jang, Y. L., et al., 2018. Self-assembled PEGylated albumin nanoparticles (SPAN) as a platform for cancer chemotherapy and imaging. *Drug Delivery.* 25(1), 1570-1578. <https://doi.org/10.1080/10717544.2018.1489430>
39. Jangid, A. K., Patel, K., Joshi, U., et al., 2022. PEGylated G4 dendrimers as a promising nanocarrier for piperlongumine delivery: Synthesis, characterization, and anticancer activity. *European Polymer Journal.* 179, 111547. <https://doi.org/10.1016/j.eurpolymj.2022.111547>
40. Farooq, T., Sohail, M., Shah, S. A., et al., 2023. Colloidal curcumin-laden pH-responsive hydrogels: A promising approach to enhance solubility, dissolution, and permeation of hydrophobic drug. *Journal of Drug Delivery Science and Technology.* 84, 104471. <https://doi.org/10.1016/j.jddst.2023.104471>
41. Wu, Z., Gao, R. P., Zhou, G. J., et al., 2021. Effect of temperature and pH on the encapsulation and release of β-carotene from octenylsuccinated oat β-glucan micelles. *Carbohydrate Polymers.* 255, 117368. <https://doi.org/10.1016/j.carbpol.2020.117368>
42. Gao, H. X., Huang, X., Xie. Y. F., et al., 2022. Improving the gastrointestinal activity of probiotics through encapsulation with biphasic gel water-in-oil emulsions. *Food & Function.* 22(13), 11455-11466. <https://doi.org/10.1039/D2FO01939F>
43. Baek, Y. J., Jeong, E. W., Lee, H. G., 2023. Encapsulation of resveratrol within size-controlled nanoliposomes: Impact on solubility, stability, cellular permeability, and oral bioavailability. *Colloids and Surfaces B: Biointerfaces.* 224, 113205. <https://doi.org/10.1016/j.colsurfb.2023.113205>
44. Rakotoarisoa, M., Angelov, B., Espinoza, S., et al., 2021. Composition-switchable liquid crystalline nanostructures as green formulations of curcumin and fish oil. *ACS Sustainable Chemistry & Engineering.* 9(44), 14821-14835. <https://doi.org/10.1021/acssuschemeng.1c04706>
45. Yuan, Y., Liu, Y., He, Y., et al., 2022. Intestinal-targeted nanotubes-in-microgels composite carriers for capsaicin delivery and their effect for alleviation of *Salmonella* induced enteritis. *Biomaterials.* 287, 121613. <https://doi.org/10.1016/j.biomaterials.2022.121613>
46. Ngema, L. M., Adeyemi, S. A., Marimuthu, T., et al., 2022. Synthesis of novel conjugated linoleic acid (CLA)-coated superparamagnetic iron oxide nanoparticles (SPIONs) for the delivery of paclitaxel with enhanced *in vitro* anti-proliferative activity on A549 lung cancer cells. *Pharmaceutics.* 14(4), 829. <https://doi.org/10.3390/pharmaceutics14040829>.
47. Mao, Y. L., Han, M. Q., Chen, C. S., et al., 2021. A biomimetic nanocomposite made of a ginger-derived exosome and an inorganic framework for high-performance delivery of oral antibodies. *Nanoscale.* 13(47), 20157-20169. <https://doi.org/10.1039/D1NR06015E>
48. Wang, C., Wang, M., Zhang, Y., Cyclic arginine-glycine-aspartic acid-modified red blood cells for drug delivery: Synthesis and *in vitro* evaluation. *Journal of Pharmaceutical Analysis.* 12(2), 324-331. <https://doi.org/10.1016/j.jpha.2021.06.003>
49. Cheng, H. B., Guo, S., Cui, Z. X., et al., 2021. Design of folic acid decorated virus-mimicking nanoparticles for enhanced oral insulin delivery. *International Journal of Pharmaceutics.* 596, 120297. <https://doi.org/10.1016/j.ijpharm.2021.120297>
50. Zhang, X. X., Chen, G. P., Fu, X., et al., 2021. Magneto-responsive microneedle robots for intestinal macromolecule delivery. *Advanced Materials.* 33(44), 2104932. <https://doi.org/10.1002/adma.202104932>.
51. Xue, M. X., Wang, J., Huang, M. G., 2022. Inulin-modified liposomes as a novel delivery system for cinnamaldehyde. *Foods.* 11(10), 1467. <https://doi.org/10.3390/foods11101467>
52. Topal, G. R., Mészáros, M., Porkoláb, G., et al., 2021. ApoE-targeting increases the transfer of solid lipid nanoparticles with donepezil cargo across a culture model of the blood-brain barrier. *Pharmaceutics.* 13(1), 38. <https://doi.org/10.3390/pharmaceutics13010038>
53. Ma, Z., Pi, J. X., Zhang, Y., et al., 2021. Enhanced anticancer efficacy of dual drug-loaded self-assembled nanostructured lipid carriers mediated by pH-responsive folic acid and human-derived cell penetrating peptide dNP2. *Pharmaceutics.* 13(5), 600. <https://doi.org/10.3390/pharmaceutics13050600>
54. Wang, Q., Wang, L., Abdullah, et al., 2022. Co-delivery of EGCG and lycopene *via* a Pickering double emulsion induced synergistic hypolipidemic effect. *Food & Function.*13(6), 3419-3430. <https://doi.org/10.1039/D2FO00169A>
55. Su, J. Q., Cai, Y. J., Zhi, Z. J., et al., 2021. Assembly of propylene glycol alginate/β-lactoglobulin composite hydrogels induced by ethanol for co-delivery of probiotics and curcumin. *Carbohydrate Polymers.* 254, 117446. <https://doi.org/10.1016/j.carbpol.2020.117446>
56. Xu, X. K., Zhao, W. X., Ye, Y. R., et al., 2021. Novel nanoliposome codelivered DHA and anthocyanidin: Characterization, *in vitro* infant digestibility, and improved cell uptake. *Journal of Agricultural and Food Chemistry.* 69(32), 9395-9406. <https://doi.org/10.1021/acs.jafc.1c02817>
57. Madureira, A. R., Campos, D., Gullon, B., et al., 2016. Fermentation of bioactive solid lipid nanoparticles by human gut microflora. *Food & Function.* 7(1), 516-529. <https://doi.org/10.1039/C5FO01004G>
58. Asadollahi, L., Mahoutforoush, A., Dorreyatim, S. S., et al., 2022. Co-Delivery of erlotinib and resveratrol *via* nanostructured lipid Carriers: A synergistically promising approach for cell proliferation prevention and ROS-Mediated apoptosis activation. *International Journal of Pharmaceutics.* 624, 122027. <https://doi.org/10.1016/j.ijpharm.2022.122027>
59. Meng, D. M., Shi, L. N., Zhu, L., et al., 2020. Coencapsulation and stability evaluation of hydrophilic and hydrophobic bioactive compounds in a cagelike phytoferritin. *Journal of Agricultural and Food Chemistry.* 68(10), 3238-3249. <https://dx.doi.org/10.1021/acs.jafc.9b06904>
60. Chen, Y. L., Tai, K. D., Ma, P. H., et al., 2021. Novel γ-cyclodextrin-metal-organic frameworks for encapsulation of curcumin with improved loading capacity, physicochemical stability and controlled release properties. *Food Chemistry.* 347, 128978. <https://doi.org/10.1016/j.foodchem.2020.128978>
61. Fu, J. J., Song, L., Guan, J. J., et al., 2021. Encapsulation of Antarctic krill oil in yeast cell microcarriers: Evaluation of oxidative stability and *in vitro* release. *Food Chemistry.* 338, 128089. <https://doi.org/10.1016/j.foodchem.2020.128089>
62. Szymandera-Buszka, K., Waszkowiak, K., Kaczmarek, A., et al., 2021. Wheat dietary fibre and soy protein as new carriers of iodine compounds for food fortification-The effect of storage conditions on the stability of potassium iodide and potassium iodate. *LWT - Food Science and Technology.* 137, 110424. <https://doi.org/10.1016/j.lwt.2020.110424>
63. Cozmuta, A. M., Purbayanto, M. A. K., Jastrzębska, A., et al., 2023. Thermal stability and *in vitro* digestion of alginate-starch-iron beads for oral delivery of iron. *Food Hydrocolloids.* 142, 108808. <https://doi.org/10.1016/j.foodhyd.2023.108808>
64. Nunes, R., Baião, A., Monteiro, D., et al., 2020. Zein nanoparticles as low-cost, safe, and effective carriers to improve the oral bioavailability of resveratrol. *Drug Delivery and Translational Research.* 10, 826-837. <https://doi.org/10.1007/s13346-020-00738-z>
65. Raza, M. A., Lim, Y. M., Lee, S. W., et al., 2021. Synthesis and characterization of hydrogels based on carboxymethyl chitosan and poly(vinylpyrrolidone) blends prepared by electron beam irradiation having anticancer efficacy, and applications as drug carrier for controlled release of drug. *Carbohydrate Polymers.* 258, 117718. <https://doi.org/10.1016/j.carbpol.2021.117718>
66. Zhang, Y., Liang, S., Zhang, J. S., et al., 2020. Preparation of whey protein isolate nanofibrils by microwave heating and its application as carriers of lipophilic bioactive substances. *LWT - Food Science and Technology.* 125, 109213. <https://doi.org/10.1016/j.lwt.2020.109213>
67. Shi, Y. M., Zhang, M., Chen, K., et al., 2022. Nano-emulsion prepared by high pressure homogenization method as a good carrier for Sichuan pepper essential oil: Preparation, stability, and bioactivity. *LWT - Food Science and Technology.* 154, 112779. <https://doi.org/10.1016/j.lwt.2021.112779>
68. Beltrán, J. D., Ricaurte, L., Estrada, K. B., et al., 2020. Effect of homogenization methods on the physical stability of nutrition grade nanoliposomes used for encapsulating high oleic palm oil. *LWT - Food Science and Technology.* 118, 108801. <https://doi.org/10.1016/j.lwt.2019.108801>
69. Zhang, S. Y., Li, Y. L., Bao, Z. J., et al., 2021. Internal cavity amplification of shell-like ferritin regulated with the change of the secondary and tertiary structure induced by PEF technology. *International Journal of Biological Macromolecules.* 182, 849-857. <https://doi.org/10.1016/j.ijbiomac.2021.04.072>
70. Tang, L. Y., Sun, Y. H., Ge, P. P., et al., 2022. Biogenetic nanocarriers with enhanced pH stability formed by zein and selectively depolymerized mushroom hyperbranched β-glucans. *International Journal of Biological Macromolecules.* 209, 1771-1783. <https://doi.org/10.1016/j.ijbiomac.2022.04.147>
71. Liu, S. Q., Zhang, J., Fu, R., et al., 2022. Improved stability and aqueous solubility of β-carotene *via* encapsulation in self-assembled bioactive oleanolic acid nanoparticles. *Food Chemistry.* 373, 131498. <https://doi.org/10.1016/j.foodchem.2021.131498>
72. Zhang, J. Y., Cui, H. C., Qiu, J. H., et al., 2023. Stability of glycosylated complexes loaded with Epigallocatechin 3-gallate (EGCG). *Food Chemistry.* 410, 135364. <https://doi.org/10.1016/j.foodchem.2022.135364>
73. Wu, H., Rui, X., Li, W., et al., 2018. Whole-grain oats (*Avena sativa L.*) as a carrier of lactic acid bacteria and a supplement rich in angiotensin I-converting enzyme inhibitory peptides through solid-state fermentation. *Food & Function.* 9(4), 2270-2281. <https://doi.org/10.1039/C7FO01578J>
